# Development of Prediction Models for Drug-Induced Cholestasis, Cirrhosis, Hepatitis, and Steatosis Based on Drug and Drug Metabolite Structures

**DOI:** 10.3389/fphar.2020.00067

**Published:** 2020-02-14

**Authors:** Hyun Kil Shin, Myung-Gyun Kang, Daeui Park, Tamina Park, Seokjoo Yoon

**Affiliations:** ^1^ Toxicoinformatics Group, Department of Predictive Toxicology, Korea Institute of Toxicology, Daejeon, South Korea; ^2^ Department of Human and Environmental Toxicology, University of Science and Technology, Daejeon, South Korea

**Keywords:** drug-induced liver injury, structure-activity relationship, structural alerts, computational toxicology, drug metabolism

## Abstract

Drug-induced liver injury (DILI) is one of the major reasons for termination of drug development. Due to the importance of predicting DILI in early phases of drug development, diverse *in silico* models have been developed to filter out DILI-causing candidates before clinical study. However, no computational models have achieved sufficient prediction power for screening DILI in early phases because 1) drugs often cause liver injury through reactive metabolites, 2) different clinical outcomes of DILI have different mechanisms, and 3) the DILI label on drugs is not clearly defined. In this study, we developed binary classification models to predict drug-induced cholestasis, cirrhosis, hepatitis, and steatosis based on the structure of drugs and their metabolites. DILI-positive data was obtained from post-market reports of drugs and DILI-negative data from DILIrank, a database curated by the Food and Drug Administration (FDA). Support vector machine (SVM) and random forest (RF) were used in developing models with nine fingerprints and one 2D molecular descriptor calculated from drug (152 DILI-positives and 102 DILI-negatives) and drug metabolite (192 DILI-positives and 126 DILI-negatives) structures. Models were developed according to Organisation for Economic Co-operation and Development (OECD) guidelines for quantitative structure-activity relationship (QSAR) validation. Internal and external validation was performed with a randomization test in order to thoroughly examine model predictability and avoid random correlation between structural features and adverse outcomes. The applicability domain was defined with a leverage method for reliable prediction of new chemicals. The best models for each liver disease were selected based on external validation results from drugs (cholestasis: 70%, cirrhosis: 90%, hepatitis: 83%, and steatosis: 85%) and drug metabolites (cholestasis: 86%, cirrhosis: 88%, hepatitis: 86%, and steatosis: 83%) with applicability domain analysis. Compiled data sets were further exploited to derive privileged substructures that were more frequent in DILI-positive sets compared to DILI-negative sets and in drug metabolite structures compared to drug structures with a Morgan fingerprint level 2.

## Introduction

Drug-induced liver injury (DILI) is caused by almost all classes of drugs and covers diverse clinical manifestations such as hepatitis, cholestasis, cirrhosis, and steatosis depending on the duration of injury and the histological location of damage (David and Hamilton, 2010). DILI is typically derived from an initial hepatocellular injury; however, the mechanisms for each clinical outcome are distinct. Drug-induced cholestasis (DICH) is caused when drug disturbs bile acid homeostasis by inhibiting hepatic transporters that mediate biliary secretion of bile acids and other organic solutes at the sinusoid (Padda et al., 2011; Sagnik and Pieter, 2018). Drug-induced cirrhosis (DIC) is developed when drugs trigger fibrogenesis, through which excessive extracellular matrix molecules (ECM) are produced. Normally, ECM is removed by matrix metalloproteinases (MMPs); however, chronic liver damage upregulates tissue inhibitors of MMPs, and thus, ECM accumulates throughout the liver (Schuppan and Afdhal, 2008; Tsochatzis et al., 2014). Hepatitis is an immune-mediated liver injury, and drug-induced hepatitis (DIH) is caused by drugs or their metabolites binding to cellular proteins (e.g., cytochrome P450) which are recognized as antigens by the immune system (Björnsson et al., 2010) and triggering an adverse immune response that resembles viral infection (Hodgson and Levi, 2004). Hepatic steatosis is characterized by fatty acid accumulation in hepatocytes due to increases in lipogenesis or decreases in fatty acid secretion (Rabinowich and Shibolet, 2015). Since mitochondria play a significant role in lipid metabolism in hepatocytes, drugs accumulating in mitochondria cause drug-induced steatosis (DIS) by interfering with mitochondrial respiration or β-oxidation (Patel and Sanyal, 2013). As each clinical outcome of liver injury is derived from distinctive mechanisms, prediction models for each disease are needed to improve predictive power and interpretability of the results.

DILI is a challenging endpoint in predictive toxicology even though numerous studies have already been conducted (Hong et al., 2018). Particularly, DILI classification models based on drug structures faced limitations in improving their performance since these are based on the assumption that similar structures have similar properties, while certain hepatotoxicants damage hepatocytes through their reactive metabolites (Cherkasov et al., 2014). There are diverse data sets available in research articles or databases that provide information on whether drugs cause DILI, yet DILI induction by reactive metabolites is only partially reported. Even in the LiverTox (NIDDK, 2012), which listed DILI causing drugs with information on their reactive metabolites, drug metabolism information is mentioned for certain drugs while it is missing for others, and thus it is impossible to judge whether the drugs are not actively metabolized, metabolites of the drugs are not reactive, or simply reactivity of drug metabolites was not examined. Due to lack of DILI labels on drug metabolites, development of prediction models for drug metabolites has been a challenging task.

As DILI has become one of the leading causes for the termination of premarket studies or drug withdrawal from the market (Fisk et al., 2018), diverse hepatotoxicity prediction models have been developed to filter out DILI causing molecular structures in early phases of drug discovery (Yang et al., 2018). *In silico* models, which predict DILI causation based on the molecular structure of drugs, are developed either with knowledge of experts to give structural alerts (SAs) or with computation based on statistical modeling (Greene and Naven, 2009). SAs are also referred to as structure-activity relationships (SARs) in which patterns of the molecular substructure of DILI causing drugs are defined according to expert knowledge and experience. SARs have been developed based on reactive drug or metabolite structures, or pharmacological mechanisms such as redox cycling, mitochondrial dysfunction, and disruption of hepatic transporters (Fisk et al., 2018). Derek hepatotoxicity module is one example of a hepatotoxicity SAR (Greene et al., 2010). The statistical model is also termed quantitative structure-activity relationship (QSAR), in which machine learning algorithms were used to train the model with chemical structure as an input and their activity as an endpoint (Hong et al., 2018). Few *in silico* models were developed to predict specific DILI endpoints such as cholestasis and steatosis based on their molecular mechanism, while numerous *in silico* models were developed to predict DILI or hepatotoxicity overall (Hewitt and Przybylak, 2016; Hong et al., 2018; Yang et al., 2018), although the molecular mechanism of each liver disease is different. For cholestasis, the bile salt export pump (BSEP) inhibition model was developed since BSEP inhibition is one molecular mechanism of hepatic cholestasis (Sagnik and Pieter, 2018). For steatosis, QSAR models were developed for cellular targets of molecular initiating events in liver steatosis adverse outcome pathways such as the pregnane X receptor, the liver X receptor, the aryl hydrocarbon receptor, the nuclear factor (erythroid-derived 2)-like 2, and the peroxisome proliferator-activated receptors (Gadaleta et al., 2018).

In this work, binary classification models that predict four types of DILI (i.e., cholestasis, cirrhosis, hepatitis, and steatosis), were developed with 257 drugs. DILI caused by drug metabolites were considered by developing models with 318 drug metabolites of the compounds. Structural differences of the compounds were considered alone in the prediction models of this work, since structural motifs in each drugs and drug metabolites are assumed to be associated with clinical outcomes. Prediction models were developed with molecular fingerprints and 2d descriptors. Support vector machine (SVM) and random forest (RF) were applied in training, and the models achieved sound prediction performance. Models were developed, validated, and analyzed according to the Organisation for Economic Co-operation and Development (OECD) guidelines for QSAR validation (OECD, 2007). Compiled data sets for drugs and drug metabolites were further examined to find privileged substructures in DILI-positive compared to DILI-negative groups, and in drug metabolites compared to drugs.

## Methods

### Drug-Induced Liver Injury Label Assignment on Drugs

Two databases were combined in this work to prepare data sets for model development: PharmaPendium (Elsevier, 2008) and DILIrank (Chen et al., 2016). Drugs that have been reported to cause cholestasis, cirrhosis, hepatitis, and steatosis were collected from PharmaPendium, and drugs with no-evidence-of-DILI (noDILI) were obtained from DILIrank. Since four clinical outcomes were the target endpoints in this work, four data tables for DICH, DIC, DIH, and DIS were separately prepared. In each data set, drugs were labeled as DILI-positive when they had over 50 post-market reports for DICH, DIC, DIH, and DIS, while drugs were labeled as DILI-negative when they met two conditions: 1) drugs were defined as noDILI in DILIrank, and 2) no post-market reports were found for the drugs in PharmaPendium. Further description of DILI label assignment is illustrated in the DILI label assignment section of the *Results and Discussions*.

### Structure Curation and Data Preprocessing

In the structure curation process, salts were removed so that active ingredients were used alone in model development. Drugs were removed when they were metallo-organics, proteins, large peptides, or mixtures. Duplicated structures were also excluded from the data set. Drugs whose molecular weight (MW) was higher than 800 Da or lower than 100 Da were removed because 1) data points were sparse within the MW range, 2) molecules with MW higher than 800 Da were commonly found to be peptides, and 3) molecules with MW lower than 100 Da were often organic solvents rather than drugs. One hundred fifty-two DILI-positive drugs and 105 DILI-negative drugs were obtained after preprocessing.

### Drug Metabolite Set Preparation

**Figure 1:**
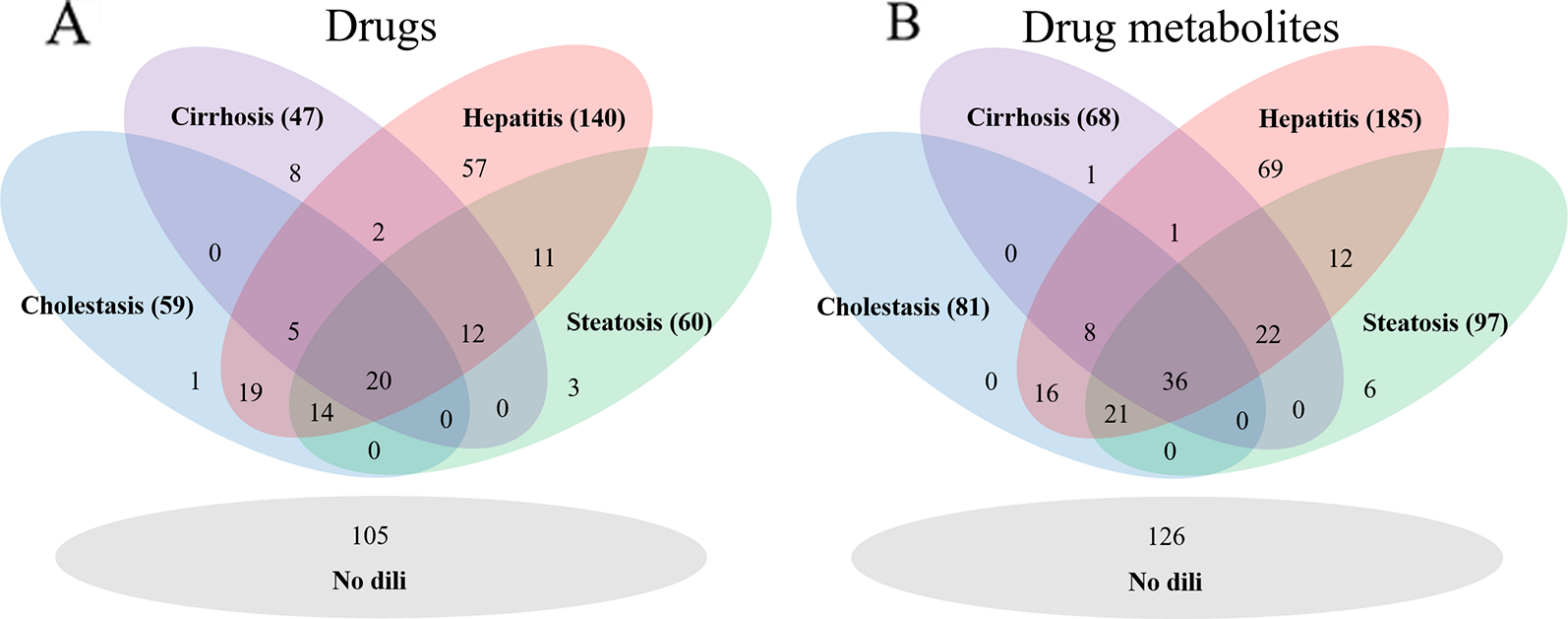
The number of drugs for each drug data set **(A)** and drug metabolite set **(B)**. The hepatitis data sets were the largest in each group.

**Figure 2:**
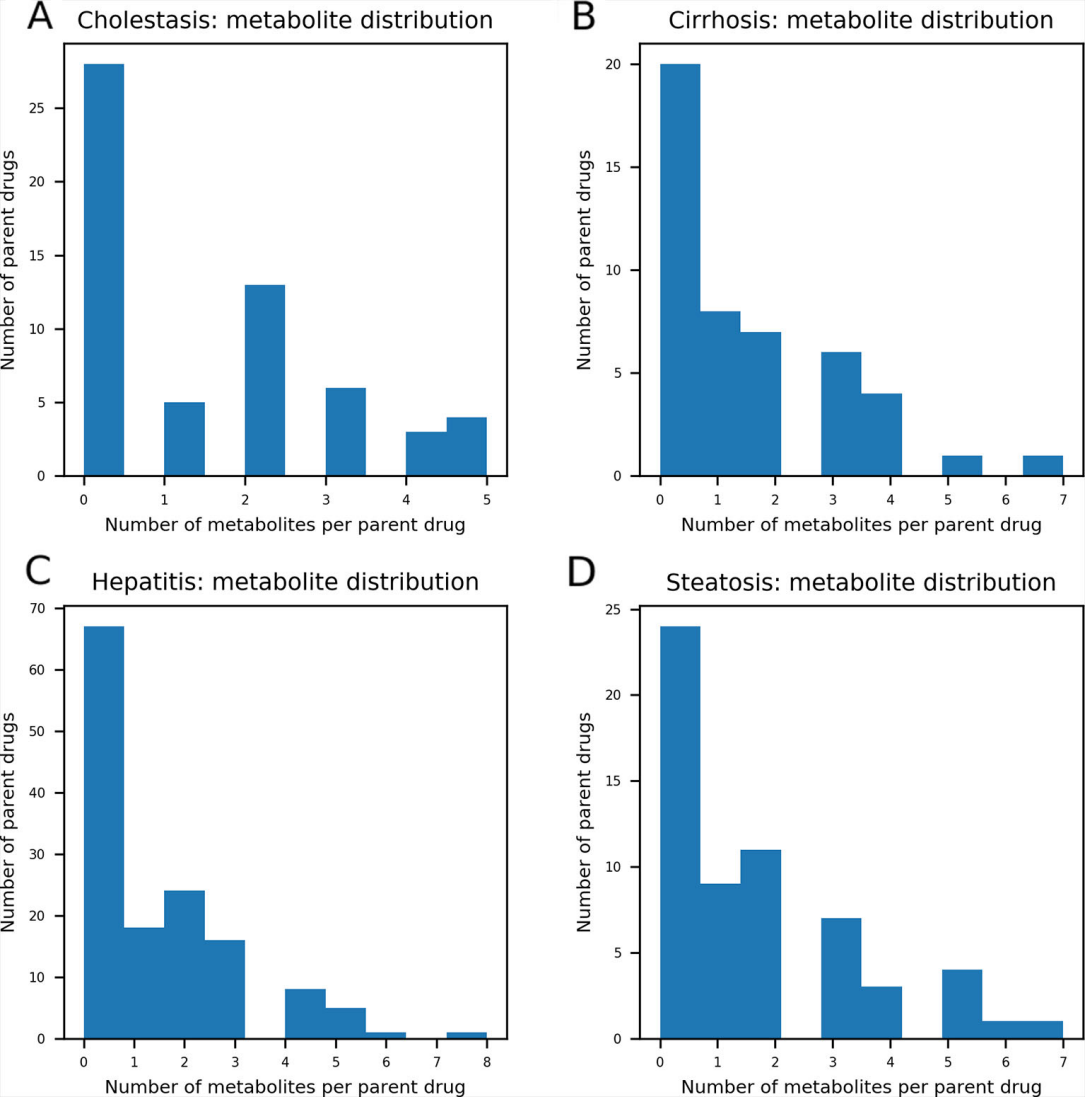
The distribution of the number of metabolites per parent compound was presented. In each data set, maximum number of drug metabolites per parent compound was five in drug-induced cholestasis [DICH, **(A)**], seven in drug-induced cirrhosis [DIC, **(B)**], eight in drug-induced hepatitis [DIH, **(C)**], and seven in drug-induced steatosis [DIS, **(D)**]. The number of parent compounds that doesn't have their metabolic products is 28 for DICH, 20 for DIC, 67 for DIH, and 24 for DIS.

As DILI labels for drug metabolites were not available, metabolites of drugs that cause DICH, DIC, DIH, and DIS were identically labeled as DILI-positive metabolites, and noDILI as DILI-negative, respectively. Metabolite structures for the DILI-positive set were obtained from the ADME database (Fujitsu, 1996), in which 78,703 entries of phase I drug metabolism and 17,692 entries of phase II drug metabolism were available (updated July 1, 2019); however, those for the DILI-negative set were relatively sparse. Therefore, DILI-negative drug metabolite sets were compiled with DILI-negative drug metabolite structures and DILI-negative parent drug structures based on the assumption that drugs whose metabolite structures were not found in the ADME database were not actively metabolized, since the ADME database covers drug metabolism of diverse compounds. Given that phase I drug metabolism produces majority of metabolic products, drug metabolites produced by human cytochrome P450 were used in this study. One hundred ninety-two DILI-positive drug metabolites were obtained from 77 DILI-positive drugs. The DILI-negative set for the drug metabolite-based prediction model was composed of 36 DILI-negative drug metabolites obtained from 20 DILI-negative drugs and 90 DILI-negative drugs whose metabolite structures were not available in the ADME database. Summary of data composition is in **Figure 1**, and the distribution of the number of metabolites per parent compound is presented in **Figure 2**.

### Feature Calculation and Curation

In this work, 2D molecular fingerprints (FP) and molecular descriptors were calculated during model development. RDKit version 2018.03.2 (Landrum, 2006) was used in FP calculation, and nine total FPs calculable in RDKit were prepared: MACCS FP (Durant et al., 2002), Avalon FP in a binary string based on presence of substructures and in an integer string based on the number of substructures (Gedeck et al., 2006), atom-pair FP (Carhart et al., 1985), topological-torsion FP (Nilakantan et al., 1987), RDKit layered FP, and Morgan FP (Rogers and Hahn, 2010) with three different depths (level 2, 3, and 4). 2D descriptors were introduced from Mordred (Moriwaki et al., 2018), which provides 1800 2D and 3D descriptors and is also implemented as a python package. 3D descriptors were not calculated in this study since an accurate 3D structure of drugs and drug metabolites were unavailable. Calculated features were removed from the data table when their standard deviation was lower than 0.01.

### Training and External Test Set

Eight data sets were compiled from DILI-positive drugs for DICH, DIC, DIH, and DIS, and DILI-negative for drug and drug metabolite structures. In the DICH, DIC, and DIS sets, the number of drugs in the DILI-positive class is almost half of that of DILI-negative, while the DIH set is imbalanced with 1.5 times more DILI-positive drugs than DILI-negative drugs. In the drug metabolite set, the number of DILI-positives for DICH, DIC, and DIS is also less than that of DILI-negatives with a smaller difference in data size between two classes, while that of DIH has a relatively larger data size difference compared to DILI-negatives. Models trained with imbalanced data tend to be improperly trained and give biased prediction outcomes on the majority class in the data. In order to prevent biased training on the model, under sampling was applied to reduce data size imbalance between positive and negative data in the training set (Zakharov et al., 2014; Klimenko et al., 2019). In this work, data imbalance was handled by designing the number of DILI-positives and DILI-negatives for each set, specifically. First, minor classes from the data set were separated with a ratio of 80 and 20% as a training set and external test set, respectively. Second, the major class was separated with a ratio that provided a similar amount of data to the minor class in the training set. This approach makes the ratio of DILI-positive to DILI-negative almost one to one in the training set of each data set. Data composition is summarized in **Table 1** with the number of positive and negative sets in training and external sets, with ratio. Even though data was randomly split with the ratio designed for each data set, it was confirmed that randomly split test set was evenly distributed within chemical space represented by randomly split training set. Chemical space similarity between the training set and the test set implied that the models trained with the training set can be successfully evaluated by the test set.

**Table 1:** Data set composition for model development.

Data set composition		Cholestasis		Cirrhosis		Hepatitis		Steatosis	
		Positive	Negative	Positive	Negative	Positive	Negative	Positive	Negative
Drug	Train	47	47	37	42	84	84	48	52
	Test	12	58	10	63	56	21	12	53
	Ratio	(8:2)	(4.5:5.5)	(8:2)	(4:6)	(6:4)	(8:2)	(8:2)	(5:5)
Drug metabolite	Train	64	69	54	56	101	100	77	81
	Test	17	57	14	70	84	26	20	45
	Ratio	(8:2)	(5.5:4.5)	(8:2)	(4.5:5.5)	(5.5:4.5)	(8:2)	(8:2)	(6.5:3.5)

### Model Development

Support vector classification (SVC) (Cortes and Vapnik, 1995), implemented in scikit-learn (Pedregosa et al., 2011), was applied during model development. The “SelectFromModel” function in the scikit-learn “feature_selection” package was applied in this work, which chooses significant features based on the weights of the model. Since SVC with linear kernel (linear SVC) defines hyperplane in the linear formula (i.e., w·x+b, where w is weight vector, x is feature vector, and b is bias vector), linear SVC was used first to subset significant features with “SelectFromModel,” and then SVC with radial basis function (RBF) kernel was applied to develop prediction models on the data set with selected features. RBF kernel projects inputted feature space into high-dimensional space in which linearly inseparable cases in the original feature space became separable with linear classifier.

Random forest (RF), implemented in scikit-learn, was also used in model building. RF model is an ensemble approach, which is composed of diverse number of decision tree (DT) models. In RF, unseen data is predicted based on majority vote from DT models. (Svetnik et al., 2003). In this study, the number of DT was set to 10 due to small size of data, and information gain was used.

### Model Performance Validation

The predictive power of the model was evaluated based on five metrics: accuracy (ACC), sensitivity (SEN), specificity (SPE), Matthew's correlation coefficients (MCC), which is regarded as a balanced binary classification performance measure on imbalanced data, and area under the curve (AUC) of recursive operating characteristic (ROC) curve.

(1) ACC = (TP+TN)/(TP+TN+FP+FN)

(2) SEN = TP/(TP+FN)

(3) SPE=TN/(TN+FP)

(4) $$MCC=\frac{TP\times TN-FP\times FN}{\sqrt{\left(TP+FP\right)\left(TP+FN\right)\left(TN+FP\right)\left(TN+FN\right)}}$$

where TP, TN, FP, FN are true positive, true negative, false positive, and false negative, respectively. Binary classification models are designed to calculate a probability score of the instances, and the instances are assigned into certain groups if the score is higher or lower than a threshold value. Better classification performance is expected when positive data obtains a higher score while negative data a lower score, which leads to higher AUC. The “Metrics” package implemented in scikit-learn was used to calculate AUC, which was measured from the ROC curve based on SEN and false positive rate (FPR), calculated by changing the threshold values.

(5) FPR=FP/(FP+TN)

For internal validation, five fold cross validation and bootstrapping were performed. In bootstrapping, data set was sampled 100 times since recommended sampling iteration was between 25 and 200. Sampled data is used to train the model, and the model is validated with un-sampled data. Statistically, 63.2% of data is sampled on average (Braga-Neto and Dougherty, 2004); therefore, a 0.632 estimator scheme is applied to each metric to evaluate model robustness and predictability as,

(6) $$metric_{0.632}\;=\;\left(1-0.632\right)metric_{sampled}+0.632metric_{unsampled}$$

where metric refers to ACC, SEN, SPE, MCC, and AUC on sampled and un-sampled data during bootstrapping.

Y-randomization tests examine the correlation between selected features and target endpoint in the model and whether it is achieved by mere chance. In the test, model development protocols were repeated after end point values were randomly shuffled. The models developed from Y-randomization are termed random models, and the model developed with correctly labeled data is an original model. Y-randomization testing was repeated 10 times to develop 10 random models, and Z-score was calculated based on MCC of random models and the original model as,

(7) $$Z=\left(MCC_{ori}-MCC_{random}^{mean}\right)/\sigma$$

where *MCC*_*ori*_ is MCC of the original model on external test set, $MCC_{random}^{mean}$ is averaged MCC of 10 random models on the external test set, and σ is standard deviation of MCC of 10 random models. Models were considered to achieve statistically valid correlation between selected features and the endpoint when Z-score exceeded 3.

### Applicability Domain Analysis

Since reliability of the model prediction outcome is dependent on the data used to train and select the model, applicability domain (AD) of the models were defined for giving reliable prediction results under the consideration of the chemical space. Leverage (*h_i_*) is a method to detect outliers or novel entities among data sets (Tropsha et al., 2003), which is calculated as,

(8) $$h_{i}=\;x_{i}^{T}{\left(X^{T}X\right)}^{-1}x_{i}$$

where *x_i_* is a feature vector of *i*-th compound, X is a feature matrix, and T is a transpose. When the leverage of certain compounds was lower than the warning leverage (h^*^), the prediction outcome was reliable since input data for the prediction is not an outlier based on the chemical space of the training data.

(9) $$h^{*}=3\left(p+1\right)/n$$

where p is the number of features selected in the model, and n is the number of training sets.

### Analysis of Privileged Substructures

In this study, substructures were called privileged substructures when their presence is higher in a certain class of data. Frequency of substructures (F) were calculated as,

(10) $$F=\left(N_{fragment,\;class}/N_{class}\right)/\left(N_{fragment,\;total}/N_{total}\right)$$

where class is the label (i.e., DILI-positive or DILI-negative), *N*_*fragment*,  *class*_ is the number of drugs that have the fragment belonging to one of the binary class, *N*_*class*_ is the total number of drugs labeled in the class, *N*_*fragment*, *total*_ is the number of drugs that have the fragment in total data set, and *N*_*total*_ is the total number of data set.

Frequency of substructures in each data set were examined based on Morgan FP in this analysis. The draw package implemented in RDKit was used to visualize substructures represented by each bit of Morgan FP level 2. In the first analysis, DILI-positive sets and DILI-negative sets in DICH, DIC, DIH, and DIS for drugs and drug metabolites were compared to find privileged substructures in DILI-positive data sets. Next, drugs and drug metabolites were compared in DICH, DIC, DIH, and DIS to find privileged substructures that were significantly influenced by drug metabolism. DILI-positive drug metabolites and drugs were compared to check which fragments were increased for bioactivation or decreased for detoxification. DILI-negative drug metabolites and drugs were also analyzed in the same way to find the detoxification effects of drug metabolism on certain molecular fragments.

## Results and Discussion

### Drug-Induced Liver Injury Label Assignment

Assigning DILI labels on drugs is a challenging task due to the wide spectrum of severity and differing injury mechanisms. Inconsistent DILI labels were often found among large DILI datasets since evidence for estimating DILI risk is differently weighted (Thakkar et al., 2018). Due to ambiguity of DILI labels, the noDILI drug list was examined by comparing it with the lists for DICH, DIC, DIH, and DIS. In DILIrank, 259 drugs were found to be noDILI; however, some noDILI drugs were found to have post-market reports on DICH (51 drugs), DIC (70 drugs), DIH (94 drugs), and DIS (73 drugs). In order to securely obtain DILI-negative drugs, drugs overlapping with DICH, DIC, DIH, and DIS were removed.

**Figure 3:**
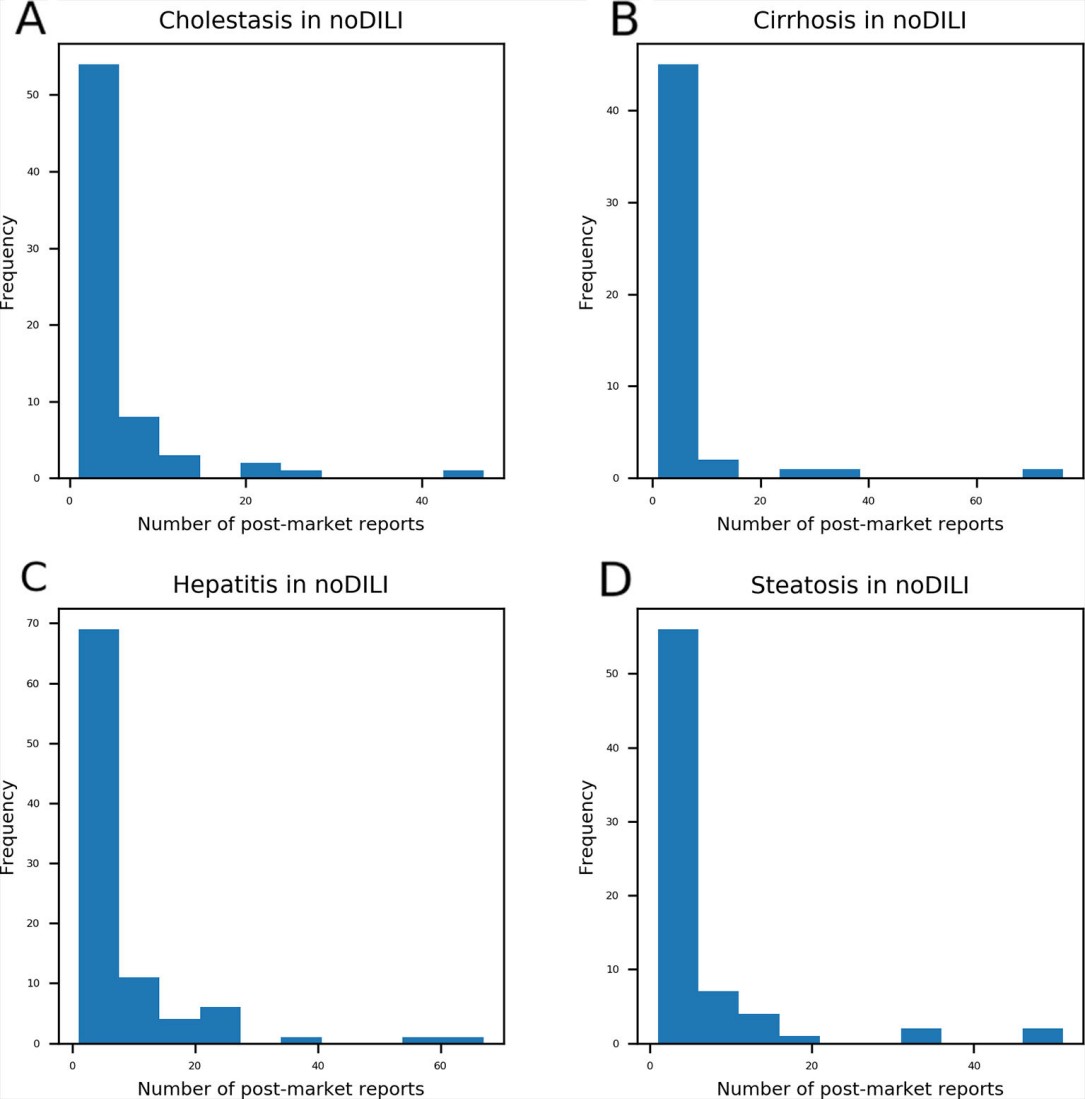
Drugs with post-market reports on cholestasis **(A)**, cirrhosis **(B)**, hepatitis **(C)**, and steatosis **(D)** were compared with drugs labeled as no-drug-induced liver injury (DILI) in the DILIrank database. Even though drugs were labeled as no-DILI in DILIrank, a number of drugs were reported to have adverse hepatic outcomes in the post-market phase. Most no-DILI drugs have less than 10 post-market reports while a few of them have a high number of post-market reports. The highest number of post-market cholestasis reports among no-DILI labeled drugs was 47 and the second highest was 26. The highest number of post-market cirrhosis reports among no-DILI labeled drugs was 76 and the second highest was 34. The highest number of post-market hepatitis reports among no-DILI labeled drugs was 67 and the second highest was 56. The highest number of post-market steatosis reports among no-DILI labeled drugs was 51 and the second highest was 47.

Distributions of post-market reports were examined from DICH, DIC, DIH, and DIS drugs overlapping with no DILI drugs (**Figure 3**). A majority of the overlapped drugs have less than 10 post-market reports; however, some of them were found to have a high number of post-market reports on DICH, DIC, DIH, and DIS. In order to define the number of post-market reports for assigning a DILI-positive label to drugs, the highest and second highest post-market reports on DICH (highest: 47 and second highest: 26), DIC (highest: 76 and second highest: 34), DIH (highest: 67 and second highest: 56), and DIS (highest: 51 and second highest: 47) of the overlapped drugs were averaged, and the mean value (50.5) became standard for DILI-positive assignment. Hence, drugs having over 50 post-market reports were defined as DILI-positive drugs in the DICH, DIC, DIH, and DIS datasets.

### Chemical Space Analysis

**Figure 4:**
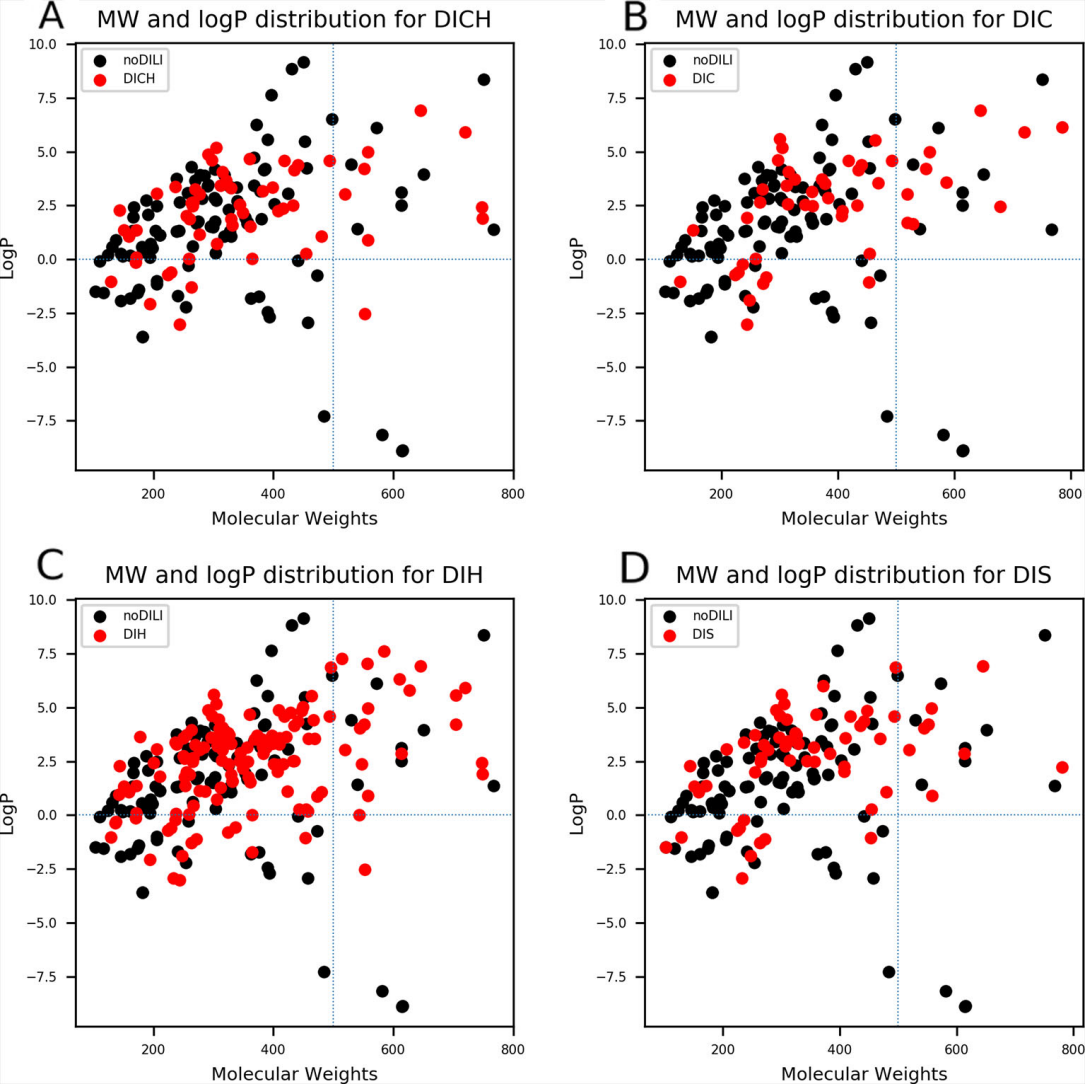
Molecular weights (MWs) and octanol/water partition coefficients (logP) were visualized for all drug data sets [**(A)** drug-induced cholestasis, **(B)**: drug-induced cirrhosis, **(C)** drug-induced hepatitis, and **(D)** drug-induced steatosis]. Dotted lines were drawn where logP was0 and molecular weight was 500. The majority of DILI-positive drugs had MWs less than 500 and logP values higher than 0.

**Figure 5:**
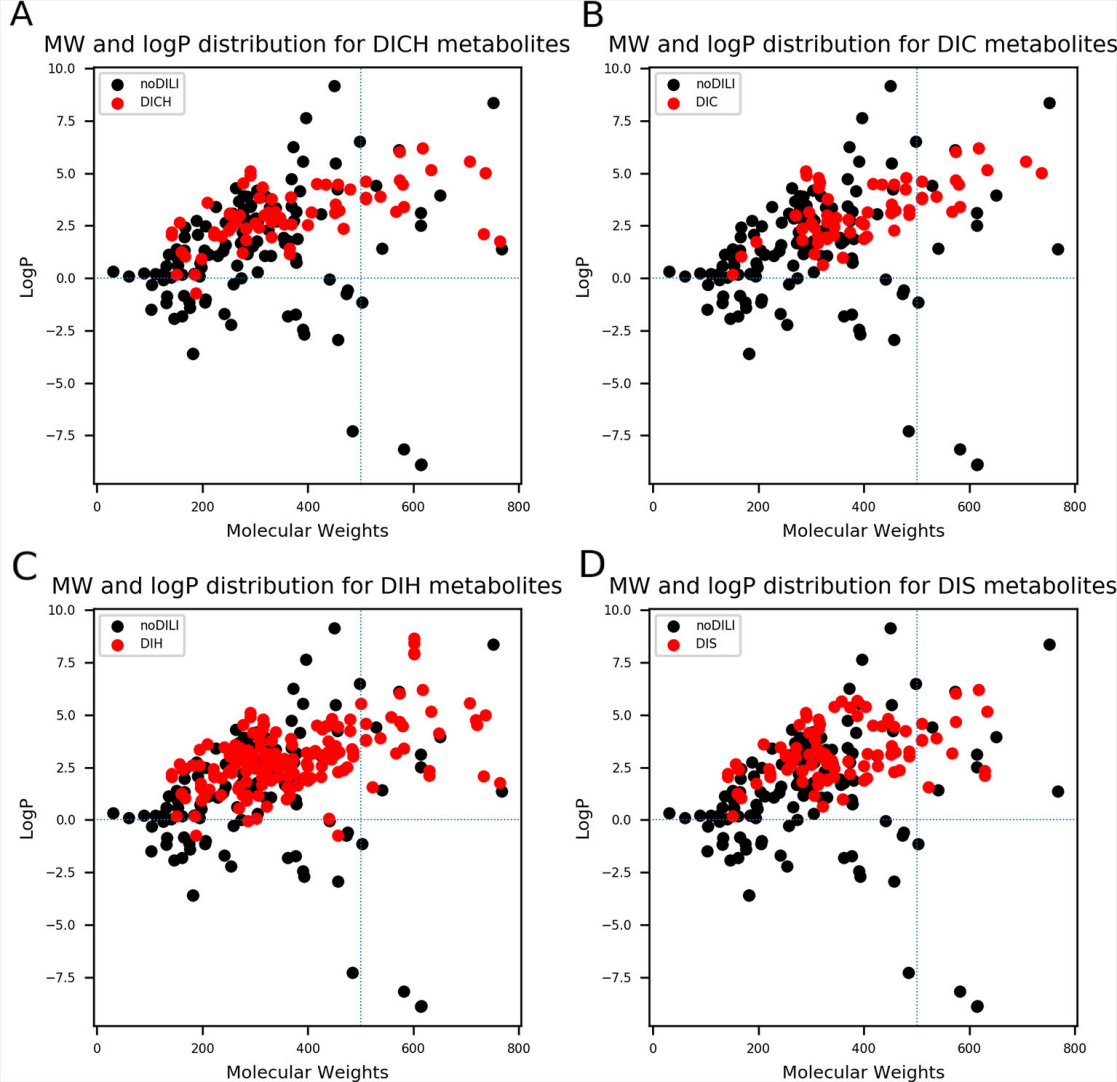
Molecular weights (MWs) and octanol/water partition coefficients (logP) were visualized for all drug metabolite data sets [**(A)** drug-induced cholestasis, **(B)**] drug-induced cirrhosis, **(C)** drug-induced hepatitis, and **(D)**: drug-induced steatosis]. Dotted lines were drawn where logP was 0 and molecular weight was 500. The majority of drug-induced liver injury (DILI)-positive drug metabolites had MWs less than 500. In particular, DILIpositive drug metabolites whose logP values were less than 0 had significantly decreased compared to DILI-positive parent drugs (**Figure 4**).

QSAR study for drug discovery normally focuses on certain classes of chemicals since it intends to predict activity variation due to moiety modification. Such approaches give high predictability to the model with relatively narrow AD. In this study, the data set is composed of drugs from diverse classes since it aims to develop a prediction model that covers heterogeneous chemical structures to secure a broader AD of the model. The chemical spaces of the data sets were visualized based on molecular weight (MW) and Wildman-Crippen octanol/water partition coefficient (logP) (Wildman and Crippen, 1999) of DILI-positive and DILI-negative sets for drugs (**Figure 4**) and drug metabolites (**Figure 5**). Chemical space analysis revealed that chemical spaces of DILI-positive drug metabolites are slightly changed compared to DILI-positive drugs. In **Figure 4**, several DILI-positive drugs were found to have logP values less than 0; however, the number of DILI-positive drug metabolites with logP values lower than 0 was decreased (**Figure 5**). Independent t-test indicated that change of mean logP value between drugs and drug metabolites were not statistically significant except cholestasis data set (**Table S1**); however, decrease of hydrophilic molecular structures in drug metabolites could potentially influence on feature selection and model training process. The shift in the chemical space of drug metabolite data sets was due to the fact that drug metabolites for drugs with low logP values were absent in the Fujitsu database. As DILI-positive drug metabolite data sets were composed of metabolite structures of DILI-positive drugs having a high logP, the mean of logP values for DILI-positive drug metabolite data sets is estimated to be higher than that for DILI-positive drug data sets even though the logP of individual drug metabolites was decreased compared to that of its parent compound. Given that drug metabolism converts lipophilic xenobiotics into hydrophilic products for rapid excretion, the chemical space shift in the drug metabolite data sets was reasonable as lipophilic drugs are more actively metabolized compared to relatively hydrophilic drugs. Moreover, the drug metabolite chemical space with increased logP was considered a more appropriate chemical space for modeling DILI causation through drug metabolites due to associations between drug lipophilicity and DILI (McEuen et al., 2017).

### Model Developments and Analysis

Binary classification models were developed with nine FPs and 2D molecular descriptors on drug and drug metabolite data sets for four types of DILI (cholestasis, cirrhosis, hepatitis, and steatosis); therefore, 80 models were trained with the SVC algorithm. Performance of the models are summarized in **Tables 2** and **3**.

**Table 2:** Drug structure-based model performance.

DILI subtype	Feature	Support vector machine																Random forest																Applicability domain analysis	
		Five fold cross validation					0.632 estimator (100 sampling)					External test set (Y-rand.)						Five fold cross validation					0.632 estimator (100 sampling)					External test set (Y-rand.)							
		SE	SP	ACC	MCC	AUC	SE	SP	ACC	MCC	AUC	SE	SP	ACC	MCC	AUC	Z	SE	SP	ACC	MCC	AUC	SE	SP	ACC	MCC	AUC	SE	SP	ACC	MCC	AUC	Z	OD/total	ratio
Cholestasis	2D descriptor	0.79 ± 0.07	0.69 ± 0.12	0.74 ± 0.08	0.48 ± 0.15	0.48 ± 0.15	0.82 ± 0.09	0.74 ± 0.08	0.78 ± 0.05	0.55 ± 0.09	0.55 ± 0.09	0.75	0.68	0.69	0.33	0.75	3.64	0.66 ± 0.19	0.8 ± 0.17	0.69 ± 0.08	0.46 ± 0.15	0.46 ± 0.15	0.71 ± 0.07	0.85 ± 0.08	0.78 ± 0.05	0.57 ± 0.1	0.57 ± 0.1	0.75	0.76	0.76	0.41	0.85	2.98	1/70	0.014
	Atompair FP	0.97 ± 0.08	0.94 ± 0.06	0.95 ± 0.06	0.89 ± 0.12	0.89 ± 0.12	0.93 ± 0.04	0.98 ± 0.04	0.95 ± 0.03	0.91 ± 0.05	0.91 ± 0.05	0.92	0.99	0.98	0.90	0.96	10.32	0.94 ± 0.08	0.96 ± 0.05	0.95 ± 0.05	0.89 ± 0.1	0.89 ± 0.1	0.95 ± 0.03	0.99 ± 0.02	0.97 ± 0.02	0.93 ± 0.03	0.93 ± 0.03	0.92	0.98	0.97	0.9	0.95	13.06	3/70	0.043
	Avalon count FP	0.8 ± 0.08	0.73 ± 0.11	0.78 ± 0.08	0.53 ± 0.15	0.53 ± 0.15	0.8 ± 0.07	0.78 ± 0.08	0.79 ± 0.04	0.57 ± 0.08	0.57 ± 0.08	0.67	0.78	0.76	0.37	0.79	2.91	0.69 ± 0.14	0.71 ± 0.14	0.67 ± 0.07	0.39 ± 0.14	0.39 ± 0.14	0.73 ± 0.08	0.85 ± 0.07	0.79 ± 0.04	0.58 ± 0.08	0.58 ± 0.08	0.75	0.74	0.74	0.39	0.75	4.76	2/70	0.029
	Avalon FP	0.75 ± 0.12	0.79 ± 0.17	0.77 ± 0.11	0.53 ± 0.22	0.53 ± 0.22	0.78 ± 0.06	0.8 ± 0.08	0.79 ± 0.04	0.58 ± 0.08	0.58 ± 0.08	0.67	0.71	0.70	0.30	0.74	3.45	0.65 ± 0.19	0.74 ± 0.15	0.68 ± 0.09	0.4 ± 0.2	0.4 ± 0.2	0.7 ± 0.08	0.8 ± 0.07	0.75 ± 0.04	0.51 ± 0.08	0.51 ± 0.08	0.58	0.74	0.71	0.26	0.73	3.82	0/70	0.000
	Morgan (lv2) FP	0.72 ± 0.17	0.79 ± 0.15	0.75 ± 0.13	0.5 ± 0.27	0.5 ± 0.27	0.68 ± 0.09	0.79 ± 0.1	0.73 ± 0.04	0.47 ± 0.08	0.47 ± 0.08	0.67	0.81	0.79	0.41	0.86	3.87	0.69 ± 0.18	0.74 ± 0.19	0.7 ± 0.14	0.43 ± 0.26	0.43 ± 0.26	0.75 ± 0.1	0.74 ± 0.08	0.74 ± 0.05	0.49 ± 0.09	0.49 ± 0.09	0.75	0.76	0.76	0.41	0.7	4.49	0/70	0.000
	Morgan (lv3) FP	0.74 ± 0.18	0.6 ± 0.21	0.64 ± 0.06	0.36 ± 0.16	0.36 ± 0.16	0.68 ± 0.15	0.71 ± 0.18	0.69 ± 0.05	0.4 ± 0.1	0.4 ± 0.1	0.59	0.78	0.75	0.30	0.81	1.58	0.65 ± 0.15	0.61 ± 0.08	0.63 ± 0.07	0.27 ± 0.16	0.27 ± 0.16	0.63 ± 0.15	0.72 ± 0.14	0.68 ± 0.04	0.37 ± 0.09	0.37 ± 0.09	0.67	0.78	0.76	0.36	0.78	4.11	2/70	0.029
	Morgan (lv4) FP	0.77 ± 0.17	0.72 ± 0.16	0.74 ± 0.15	0.48 ± 0.31	0.48 ± 0.31	0.77 ± 0.07	0.73 ± 0.08	0.75 ± 0.05	0.5 ± 0.09	0.5 ± 0.09	0.75	0.73	0.73	0.38	0.75	3.31	0.83 ± 0.1	0.73 ± 0.15	0.78 ± 0.11	0.56 ± 0.21	0.56 ± 0.21	0.81 ± 0.08	0.74 ± 0.07	0.77 ± 0.05	0.55 ± 0.1	0.55 ± 0.1	0.75	0.74	0.74	0.39	0.76	2.9	3/70	0.043
	Layer FP	0.64 ± 0.2	0.72 ± 0.12	0.67 ± 0.12	0.35 ± 0.21	0.35 ± 0.21	0.71 ± 0.1	0.76 ± 0.12	0.73 ± 0.05	0.48 ± 0.1	0.48 ± 0.1	0.75	0.68	0.69	0.33	0.77	2.12	0.78 ± 0.12	0.77 ± 0.15	0.77 ± 0.05	0.55 ± 0.1	0.55 ± 0.1	0.72 ± 0.1	0.76 ± 0.09	0.74 ± 0.04	0.48 ± 0.08	0.48 ± 0.08	0.67	0.72	0.71	0.31	0.74	3.55	0/70	0.000
	MACCS FP	0.79 ± 0.17	0.69 ± 0.17	0.72 ± 0.11	0.46 ± 0.21	0.46 ± 0.21	0.73 ± 0.1	0.78 ± 0.12	0.76 ± 0.04	0.52 ± 0.08	0.52 ± 0.08	0.67	0.78	0.76	0.37	0.83	2.61	***0.77 ± 0.11***	***0.65 ± 0.1***	***0.71 ± 0.06***	***0.42 ± 0.14***	***0.42 ± 0.14***	***0.75 ± 0.1***	***0.75 ± 0.07***	***0.75 ± 0.04***	***0.5 ± 0.09***	***0.5 ± 0.09***	***0.8***	***0.7***	***0.7***	***0.3***	***0.8***	***3.14***	***0/70***	***####***
	Torsion FP	0.84 ± 0.12	0.94 ± 0.06	0.9 ± 0.05	0.78 ± 0.11	0.78 ± 0.11	0.85 ± 0.06	0.95 ± 0.03	0.9 ± 0.03	0.8 ± 0.05	0.8 ± 0.05	0.92	0.97	0.96	0.86	0.94	6.75	0.81 ± 0.16	0.94 ± 0.05	0.88 ± 0.08	0.76 ± 0.16	0.76 ± 0.16	0.81 ± 0.07	0.96 ± 0.04	0.88 ± 0.04	0.78 ± 0.07	0.78 ± 0.07	0.92	0.93	0.93	0.78	0.95	6.89	4/70	0.057
Cirrhosis	2D descriptor	0.78 ± 0.14	0.71 ± 0.2	0.72 ± 0.12	0.46 ± 0.22	0.46 ± 0.22	0.72 ± 0.12	0.79 ± 0.07	0.76 ± 0.04	0.51 ± 0.08	0.51 ± 0.08	0.80	0.75	0.75	0.41	0.84	3.07	0.73 ± 0.06	0.82 ± 0.12	0.79 ± 0.08	0.57 ± 0.15	0.57 ± 0.15	0.79 ± 0.09	0.87 ± 0.06	0.83 ± 0.05	0.67 ± 0.09	0.67 ± 0.09	0.6	0.79	0.77	0.32	0.82	3.06	5/73	0.068
	Atompair FP	0.93 ± 0.1	0.99 ± 0.04	0.96 ± 0.05	0.92 ± 0.08	0.92 ± 0.08	0.96 ± 0.04	0.99 ± 0.03	0.98 ± 0.03	0.95 ± 0.05	0.95 ± 0.05	0.90	0.95	0.95	0.79	0.98	8.39	0.87 ± 0.09	0.98 ± 0.04	0.93 ± 0.02	0.86 ± 0.04	0.86 ± 0.04	0.94 ± 0.05	0.98 ± 0.03	0.96 ± 0.02	0.92 ± 0.05	0.92 ± 0.05	0.9	0.93	0.93	0.75	0.99	7.74	3/73	0.041
	Avalon count FP	0.72 ± 0.12	0.9 ± 0.09	0.82 ± 0.1	0.63 ± 0.19	0.63 ± 0.19	0.83 ± 0.09	0.86 ± 0.08	0.85 ± 0.04	0.7 ± 0.07	0.7 ± 0.07	0.60	0.78	0.75	0.30	0.71	3.20	0.84 ± 0.11	0.8 ± 0.18	0.81 ± 0.13	0.62 ± 0.25	0.62 ± 0.25	0.79 ± 0.09	0.89 ± 0.06	0.85 ± 0.05	0.69 ± 0.09	0.69 ± 0.09	0.7	0.88	0.85	0.51	0.81	5.19	1/73	0.014
	Avalon FP	0.71 ± 0.19	0.89 ± 0.14	0.8 ± 0.11	0.62 ± 0.21	0.62 ± 0.21	0.8 ± 0.09	0.85 ± 0.06	0.82 ± 0.04	0.65 ± 0.07	0.65 ± 0.07	0.70	0.90	0.87	0.54	0.86	4.83	0.67 ± 0.15	0.81 ± 0.08	0.74 ± 0.1	0.48 ± 0.18	0.48 ± 0.18	0.74 ± 0.09	0.87 ± 0.07	0.81 ± 0.04	0.63 ± 0.08	0.63 ± 0.08	0.7	0.83	0.81	0.43	0.87	5.81	0/73	0.000
	Morgan (lv2) FP	0.72 ± 0.17	0.84 ± 0.15	0.78 ± 0.1	0.57 ± 0.22	0.57 ± 0.22	0.76 ± 0.08	0.89 ± 0.07	0.83 ± 0.04	0.66 ± 0.07	0.66 ± 0.07	0.70	0.73	0.73	0.32	0.78	2.39	0.74 ± 0.19	0.87 ± 0.1	0.82 ± 0.04	0.64 ± 0.07	0.64 ± 0.07	0.74 ± 0.08	0.89 ± 0.08	0.82 ± 0.04	0.65 ± 0.07	0.65 ± 0.07	0.9	0.91	0.91	0.71	0.91	7.21	3/73	0.041
	Morgan (lv3) FP	0.75 ± 0.19	0.82 ± 0.07	0.78 ± 0.1	0.55 ± 0.23	0.55 ± 0.23	0.69 ± 0.09	0.84 ± 0.07	0.78 ± 0.04	0.54 ± 0.08	0.54 ± 0.08	0.80	0.85	0.84	0.53	0.89	3.99	0.72 ± 0.15	0.87 ± 0.08	0.8 ± 0.1	0.6 ± 0.19	0.6 ± 0.19	0.67 ± 0.1	0.85 ± 0.09	0.77 ± 0.03	0.53 ± 0.07	0.53 ± 0.07	0.7	0.9	0.87	0.54	0.86	5.61	3/73	0.041
	Morgan (lv4) FP	0.76 ± 0.07	0.82 ± 0.14	0.79 ± 0.06	0.58 ± 0.11	0.58 ± 0.11	0.81 ± 0.09	0.87 ± 0.05	0.85 ± 0.04	0.69 ± 0.08	0.69 ± 0.08	0.80	0.80	0.80	0.47	0.82	4.70	0.72 ± 0.14	0.84 ± 0.12	0.79 ± 0.07	0.55 ± 0.12	0.55 ± 0.12	0.74 ± 0.08	0.89 ± 0.07	0.83 ± 0.03	0.65 ± 0.06	0.65 ± 0.06	0.8	0.91	0.9	0.64	0.88	7.06	0/73	0.000
	Layer FP	0.84 ± 0.1	0.89 ± 0.12	0.86 ± 0.1	0.72 ± 0.2	0.72 ± 0.2	0.93 ± 0.06	0.88 ± 0.07	0.9 ± 0.04	0.81 ± 0.08	0.81 ± 0.08	0.70	0.88	0.86	0.51	0.91	3.90	***0.68 ± 0.1***	***0.74 ± 0.09***	***0.7 ± 0.07***	***0.41 ± 0.11***	***0.41 ± 0.11***	***0.82 ± 0.08***	***0.92 ± 0.06***	***0.87 ± 0.04***	***0.75 ± 0.08***	***0.75 ± 0.08***	***0.8***	***0.9***	***0.9***	***0.6***	***0.9***	***4.23***	***0/73***	***####***
	MACCS FP	0.78 ± 0.22	0.88 ± 0.14	0.84 ± 0.14	0.64 ± 0.31	0.64 ± 0.31	0.79 ± 0.1	0.84 ± 0.08	0.82 ± 0.05	0.63 ± 0.09	0.63 ± 0.09	0.70	0.88	0.86	0.51	0.85	4.28	0.66 ± 0.17	0.8 ± 0.25	0.73 ± 0.2	0.47 ± 0.38	0.47 ± 0.38	0.79 ± 0.08	0.87 ± 0.09	0.83 ± 0.04	0.67 ± 0.09	0.67 ± 0.09	0.7	0.85	0.82	0.46	0.83	4.83	0/73	0.000
	Torsion FP	0.85 ± 0.15	0.98 ± 0.06	0.93 ± 0.05	0.86 ± 0.1	0.86 ± 0.1	0.88 ± 0.05	0.99 ± 0.02	0.94 ± 0.03	0.88 ± 0.05	0.88 ± 0.05	0.90	0.97	0.96	0.84	1.00	5.26	0.83 ± 0.05	0.97 ± 0.07	0.92 ± 0.03	0.83 ± 0.06	0.83 ± 0.06	0.86 ± 0.07	0.96 ± 0.03	0.91 ± 0.03	0.83 ± 0.06	0.83 ± 0.06	0.9	0.98	0.97	0.88	0.9	7.81	4/73	0.055
Hepatitis	2D descriptor	***0.8 ± 0.11***	***0.7 ± 0.16***	***0.75 ± 0.05***	***0.52 ± 0.1***	***0.52 ± 0.1***	***0.74 ± 0.07***	***0.75 ± 0.05***	***0.75 ± 0.03***	***0.49 ± 0.06***	***0.49 ± 0.06***	***###***	***###***	***0.83***	***0.61***	***0.85***	***5.06***	0.62 ± 0.08	0.71 ± 0.09	0.67 ± 0.06	0.33 ± 0.12	0.33 ± 0.12	0.76 ± 0.06	0.84 ± 0.05	0.8 ± 0.03	0.61 ± 0.07	0.61 ± 0.07	0.67	0.76	0.69	0.37	0.77	9.17	***0/77***	***####***
	Atompair FP	0.99 ± 0.04	0.98 ± 0.04	0.99 ± 0.03	0.97 ± 0.06	0.97 ± 0.06	1 ± 0.02	0.98 ± 0.02	0.99 ± 0.02	0.98 ± 0.03	0.98 ± 0.03	0.99	0.96	0.98	0.94	1.00	10.85	0.99 ± 0.02	0.99 ± 0.03	0.99 ± 0.02	0.98 ± 0.03	0.98 ± 0.03	0.98 ± 0.02	0.99 ± 0.01	0.99 ± 0.01	0.97 ± 0.02	0.97 ± 0.02	0.95	0.95	0.95	0.88	0.98	8.95	7/77	0.091
	Avalon count FP	0.67 ± 0.11	0.85 ± 0.04	0.76 ± 0.07	0.52 ± 0.12	0.52 ± 0.12	0.74 ± 0.07	0.83 ± 0.06	0.79 ± 0.04	0.57 ± 0.08	0.57 ± 0.08	0.66	0.67	0.66	0.28	0.77	4.06	0.6 ± 0.04	0.73 ± 0.15	0.67 ± 0.07	0.34 ± 0.16	0.34 ± 0.16	0.76 ± 0.06	0.82 ± 0.06	0.79 ± 0.04	0.58 ± 0.07	0.58 ± 0.07	0.71	0.62	0.69	0.3	0.68	3.58	5/77	0.065
	Avalon FP	0.71 ± 0.11	0.83 ± 0.12	0.77 ± 0.12	0.53 ± 0.21	0.53 ± 0.21	0.79 ± 0.07	0.8 ± 0.07	0.79 ± 0.03	0.58 ± 0.05	0.58 ± 0.05	0.80	0.81	0.80	0.55	0.81	5.24	0.64 ± 0.13	0.8 ± 0.07	0.73 ± 0.05	0.45 ± 0.11	0.45 ± 0.11	0.75 ± 0.06	0.82 ± 0.06	0.79 ± 0.03	0.58 ± 0.07	0.58 ± 0.07	0.62	0.76	0.66	0.33	0.72	6.21	0/77	0.000
	Morgan (lv2) FP	0.61 ± 0.15	0.8 ± 0.09	0.71 ± 0.11	0.41 ± 0.22	0.41 ± 0.22	0.73 ± 0.09	0.77 ± 0.06	0.75 ± 0.04	0.5 ± 0.07	0.5 ± 0.07	0.70	0.77	0.72	0.41	0.77	4.91	0.71 ± 0.12	0.81 ± 0.09	0.76 ± 0.09	0.51 ± 0.19	0.51 ± 0.19	0.71 ± 0.06	0.81 ± 0.06	0.76 ± 0.03	0.53 ± 0.06	0.53 ± 0.06	0.67	0.81	0.7	0.41	0.82	6.12	8/77	0.104
	Morgan (lv3) FP	0.66 ± 0.14	0.71 ± 0.07	0.69 ± 0.08	0.37 ± 0.15	0.37 ± 0.15	0.74 ± 0.07	0.74 ± 0.09	0.74 ± 0.04	0.48 ± 0.07	0.48 ± 0.07	0.72	0.81	0.74	0.46	0.81	5.03	0.69 ± 0.13	0.76 ± 0.05	0.73 ± 0.08	0.46 ± 0.17	0.46 ± 0.17	0.72 ± 0.07	0.75 ± 0.06	0.73 ± 0.03	0.47 ± 0.07	0.47 ± 0.07	0.76	0.81	0.77	0.51	0.79	4.69	6/77	0.078
	Morgan (lv4) FP	0.7 ± 0.24	0.85 ± 0.07	0.75 ± 0.13	0.54 ± 0.21	0.54 ± 0.21	0.69 ± 0.1	0.78 ± 0.09	0.73 ± 0.04	0.47 ± 0.07	0.47 ± 0.07	0.73	0.72	0.73	0.40	0.73	4.51	0.68 ± 0.13	0.81 ± 0.05	0.75 ± 0.08	0.5 ± 0.15	0.5 ± 0.15	0.76 ± 0.06	0.8 ± 0.06	0.78 ± 0.03	0.55 ± 0.06	0.55 ± 0.06	0.84	0.67	0.8	0.49	0.8	6.33	2/77	0.026
	Layer FP	0.72 ± 0.1	0.86 ± 0.08	0.79 ± 0.06	0.58 ± 0.1	0.58 ± 0.1	0.72 ± 0.06	0.84 ± 0.08	0.78 ± 0.04	0.56 ± 0.06	0.56 ± 0.06	0.73	0.86	0.77	0.52	0.83	3.63	0.69 ± 0.09	0.86 ± 0.06	0.78 ± 0.04	0.56 ± 0.07	0.56 ± 0.07	0.7 ± 0.06	0.86 ± 0.07	0.78 ± 0.03	0.58 ± 0.06	0.58 ± 0.06	0.68	0.67	0.68	0.31	0.67	2.97	0/77	0.000
	MACCS FP	0.67 ± 0.14	0.82 ± 0.06	0.74 ± 0.05	0.49 ± 0.08	0.49 ± 0.08	0.69 ± 0.08	0.8 ± 0.07	0.75 ± 0.04	0.49 ± 0.06	0.49 ± 0.06	0.66	0.77	0.68	0.36	0.77	2.71	0.66 ± 0.16	0.78 ± 0.13	0.73 ± 0.06	0.47 ± 0.08	0.47 ± 0.08	0.71 ± 0.06	0.84 ± 0.06	0.78 ± 0.03	0.56 ± 0.07	0.56 ± 0.07	0.68	0.71	0.69	0.35	0.78	4.13	0/77	0.000
	Torsion FP	0.85 ± 0.07	0.97 ± 0.03	0.91 ± 0.03	0.82 ± 0.05	0.82 ± 0.05	0.89 ± 0.03	0.98 ± 0.02	0.94 ± 0.02	0.87 ± 0.03	0.87 ± 0.03	0.85	0.96	0.87	0.72	0.94	6.21	0.85 ± 0.11	0.98 ± 0.04	0.91 ± 0.06	0.84 ± 0.11	0.84 ± 0.11	0.89 ± 0.03	0.97 ± 0.02	0.93 ± 0.02	0.86 ± 0.04	0.86 ± 0.04	0.86	0.95	0.88	0.74	0.96	6.82	6/77	0.078
Steatosis	2D descriptor	0.71 ± 0.14	0.72 ± 0.22	0.71 ± 0.1	0.43 ± 0.21	0.43 ± 0.21	0.74 ± 0.07	0.8 ± 0.06	0.77 ± 0.04	0.55 ± 0.08	0.55 ± 0.08	0.59	0.78	0.74	0.31	0.72	2.40	0.64 ± 0.16	0.74 ± 0.1	0.7 ± 0.08	0.38 ± 0.17	0.38 ± 0.17	0.65 ± 0.09	0.83 ± 0.06	0.76 ± 0.04	0.49 ± 0.08	0.49 ± 0.08	0.75	0.66	0.68	0.32	0.7	3.01	1/65	0.015
	Atompair FP	0.99 ± 0.04	0.95 ± 0.08	0.96 ± 0.04	0.93 ± 0.07	0.93 ± 0.07	0.93 ± 0.04	0.95 ± 0.04	0.94 ± 0.03	0.88 ± 0.05	0.88 ± 0.05	0.84	0.95	0.93	0.76	0.94	6.86	0.96 ± 0.07	0.97 ± 0.04	0.96 ± 0.04	0.92 ± 0.07	0.92 ± 0.07	0.9 ± 0.04	0.99 ± 0.02	0.95 ± 0.02	0.91 ± 0.04	0.91 ± 0.04	0.92	0.98	0.97	0.9	0.98	8.64	4/65	0.062
	Avalon count FP	0.63 ± 0.13	0.77 ± 0.08	0.69 ± 0.08	0.38 ± 0.15	0.38 ± 0.15	0.69 ± 0.08	0.79 ± 0.08	0.74 ± 0.04	0.48 ± 0.08	0.48 ± 0.08	0.67	0.72	0.71	0.32	0.73	2.66	0.65 ± 0.23	0.73 ± 0.12	0.68 ± 0.12	0.39 ± 0.16	0.39 ± 0.16	0.72 ± 0.08	0.82 ± 0.07	0.77 ± 0.05	0.54 ± 0.09	0.54 ± 0.09	0.67	0.7	0.69	0.29	0.68	2.3	3/65	0.046
	Avalon FP	0.66 ± 0.22	0.8 ± 0.1	0.73 ± 0.08	0.45 ± 0.19	0.45 ± 0.19	0.69 ± 0.09	0.76 ± 0.09	0.73 ± 0.05	0.45 ± 0.09	0.45 ± 0.09	0.75	0.85	0.84	0.53	0.72	3.51	0.68 ± 0.16	0.72 ± 0.17	0.71 ± 0.1	0.41 ± 0.22	0.41 ± 0.22	0.69 ± 0.09	0.83 ± 0.09	0.76 ± 0.05	0.54 ± 0.1	0.54 ± 0.1	0.83	0.74	0.75	0.46	0.81	5.24	0/65	0.000
	Morgan (lv2) FP	0.69 ± 0.1	0.67 ± 0.26	0.67 ± 0.11	0.37 ± 0.21	0.37 ± 0.21	0.65 ± 0.07	0.82 ± 0.08	0.74 ± 0.04	0.48 ± 0.07	0.48 ± 0.07	0.84	0.76	0.77	0.48	0.82	6.00	0.63 ± 0.17	0.71 ± 0.15	0.69 ± 0.09	0.36 ± 0.15	0.36 ± 0.15	0.72 ± 0.07	0.7 ± 0.08	0.71 ± 0.04	0.42 ± 0.08	0.42 ± 0.08	0.67	0.79	0.77	0.39	0.69	2.39	1/65	0.015
	Morgan (lv3) FP	0.78 ± 0.17	0.81 ± 0.16	0.77 ± 0.13	0.57 ± 0.24	0.57 ± 0.24	0.73 ± 0.12	0.79 ± 0.1	0.76 ± 0.06	0.53 ± 0.1	0.53 ± 0.1	0.75	0.78	0.77	0.44	0.76	5.25	0.81 ± 0.06	0.68 ± 0.1	0.75 ± 0.06	0.49 ± 0.13	0.49 ± 0.13	0.78 ± 0.08	0.75 ± 0.07	0.76 ± 0.04	0.53 ± 0.08	0.53 ± 0.08	0.75	0.74	0.74	0.39	0.78	4.59	0/65	0.000
	Morgan (lv4) FP	0.7 ± 0.22	0.87 ± 0.06	0.77 ± 0.1	0.57 ± 0.17	0.57 ± 0.17	0.66 ± 0.11	0.86 ± 0.08	0.76 ± 0.05	0.53 ± 0.09	0.53 ± 0.09	0.92	0.80	0.82	0.59	0.87	5.28	0.75 ± 0.18	0.66 ± 0.12	0.71 ± 0.07	0.42 ± 0.17	0.42 ± 0.17	0.81 ± 0.08	0.8 ± 0.08	0.8 ± 0.04	0.61 ± 0.08	0.61 ± 0.08	0.67	0.79	0.77	0.39	0.78	6.92	3/65	0.046
	Layer FP	***0.77 ± 0.15***	***0.85 ± 0.06***	***0.8 ± 0.09***	***0.62 ± 0.16***	***0.62 ± 0.16***	***0.75 ± 0.08***	***0.83 ± 0.06***	***0.79 ± 0.04***	***0.58 ± 0.08***	***0.58 ± 0.08***	***###***	***###***	***0.85***	***0.60***	***0.92***	***5.10***	0.74 ± 0.15	0.8 ± 0.1	0.77 ± 0.09	0.55 ± 0.18	0.55 ± 0.18	0.77 ± 0.06	0.85 ± 0.06	0.81 ± 0.04	0.63 ± 0.07	0.63 ± 0.07	0.67	0.79	0.77	0.39	0.73	3.27	***0/65***	***####***
	MACCS FP	0.75 ± 0.18	0.72 ± 0.13	0.72 ± 0.13	0.46 ± 0.26	0.46 ± 0.26	0.76 ± 0.09	0.76 ± 0.09	0.76 ± 0.04	0.52 ± 0.08	0.52 ± 0.08	0.75	0.78	0.77	0.44	0.80	3.89	0.65 ± 0.12	0.78 ± 0.11	0.72 ± 0.08	0.44 ± 0.16	0.44 ± 0.16	0.7 ± 0.09	0.79 ± 0.07	0.75 ± 0.04	0.5 ± 0.08	0.5 ± 0.08	0.83	0.68	0.71	0.4	0.81	2.95	0/65	0.000
	Torsion FP	0.84 ± 0.1	0.99 ± 0.04	0.91 ± 0.06	0.83 ± 0.11	0.83 ± 0.11	0.82 ± 0.06	0.98 ± 0.03	0.9 ± 0.03	0.8 ± 0.06	0.8 ± 0.06	0.84	0.99	0.96	0.85	0.96	7.38	0.83 ± 0.09	0.97 ± 0.04	0.9 ± 0.05	0.8 ± 0.09	0.8 ± 0.09	0.84 ± 0.06	0.99 ± 0.02	0.92 ± 0.03	0.84 ± 0.05	0.84 ± 0.05	0.92	0.98	0.97	0.9	0.95	8.8	3/65	0.046

*SE, sensitivity; SP, specificity; ACC, accuracy; MCC, Matthew's correlation coefficient; AUC, area under the ROC curve; OD, out-of-domain. Bolded and italic texts highlighted best performance among models.

**Table 3:** Drug metabolite structure-based model performance.

DILI subtype	Feature	Support vector machine																Random forest																Applicability domain analysis	
		Five fold cross validation					0.632 estimator (100 sampling)					External test set (Y-rand.)						Five fold cross validation					0.632 estimator (100 sampling)					External test set (Y-rand.)							
		SE	SP	ACC	MCC	AUC	SE	SP	ACC	MCC	AUC	SE	SP	ACC	MCC	AUC	Z	SE	SP	ACC	MCC	AUC	SE	SP	ACC	MCC	AUC	SE	SP	ACC	MCC	AUC	Z	OD/total	ratio
Cholestasis	2D descriptor	0.88 ± 0.08	0.79 ± 0.1	0.83 ± 0.05	0.67 ± 0.09	0.67 ± 0.09	0.9 ± 0.05	0.84 ± 0.07	0.87 ± 0.03	0.74 ± 0.07	0.74 ± 0.07	0.88	0.83	0.84	0.61	0.91	4.28	0.77 ± 0.15	0.77 ± 0.14	0.78 ± 0.07	0.55 ± 0.14	0.55 ± 0.14	0.8 ± 0.07	0.88 ± 0.05	0.84 ± 0.04	0.69 ± 0.08	0.69 ± 0.08	0.82	0.87	0.86	0.63	0.88	7.28	2/74	0.027
	Atompair FP	0.87 ± 0.09	0.77 ± 0.13	0.81 ± 0.08	0.64 ± 0.13	0.64 ± 0.13	0.88 ± 0.07	0.78 ± 0.07	0.84 ± 0.03	0.67 ± 0.06	0.67 ± 0.06	0.88	0.79	0.81	0.56	0.92	8.04	0.83 ± 0.06	0.74 ± 0.08	0.78 ± 0.02	0.57 ± 0.04	0.57 ± 0.04	0.85 ± 0.07	0.88 ± 0.06	0.86 ± 0.04	0.73 ± 0.08	0.73 ± 0.08	0.94	0.81	0.84	0.64	0.93	10.19	5/74	0.068
	Avalon count FP	0.75 ± 0.12	0.75 ± 0.11	0.75 ± 0.03	0.51 ± 0.05	0.51 ± 0.05	0.82 ± 0.06	0.79 ± 0.08	0.8 ± 0.04	0.61 ± 0.08	0.61 ± 0.08	0.82	0.77	0.78	0.5	0.84	2.8	0.72 ± 0.07	0.77 ± 0.1	0.74 ± 0.07	0.49 ± 0.15	0.49 ± 0.15	0.79 ± 0.08	0.85 ± 0.06	0.82 ± 0.04	0.64 ± 0.09	0.64 ± 0.09	0.71	0.86	0.83	0.51	0.84	4.7	4/74	0.054
	Avalon FP	0.87 ± 0.1	0.72 ± 0.17	0.78 ± 0.1	0.59 ± 0.19	0.59 ± 0.19	0.89 ± 0.05	0.77 ± 0.06	0.83 ± 0.03	0.67 ± 0.06	0.67 ± 0.06	0.71	0.77	0.76	0.41	0.81	4.3	0.83 ± 0.06	0.75 ± 0.06	0.79 ± 0.04	0.58 ± 0.08	0.58 ± 0.08	0.84 ± 0.06	0.87 ± 0.06	0.86 ± 0.03	0.72 ± 0.07	0.72 ± 0.07	0.77	0.91	0.89	0.65	0.87	7.73	0/74	0.000
	Morgan (lv2) FP	0.79 ± 0.07	0.77 ± 0.1	0.78 ± 0.08	0.56 ± 0.18	0.56 ± 0.18	0.75 ± 0.06	0.84 ± 0.04	0.79 ± 0.03	0.59 ± 0.06	0.59 ± 0.06	0.94	0.79	0.82	0.6	0.9	4.46	0.76 ± 0.13	0.88 ± 0.08	0.82 ± 0.07	0.64 ± 0.15	0.64 ± 0.15	0.72 ± 0.06	0.88 ± 0.05	0.79 ± 0.03	0.6 ± 0.05	0.6 ± 0.05	0.94	0.84	0.86	0.67	0.96	6.71	3/74	0.041
	Morgan (lv3) FP	0.75 ± 0.14	0.9 ± 0.06	0.81 ± 0.09	0.64 ± 0.14	0.64 ± 0.14	0.71 ± 0.07	0.91 ± 0.07	0.8 ± 0.04	0.63 ± 0.07	0.63 ± 0.07	0.88	0.9	0.9	0.71	0.92	6.78	0.73 ± 0.1	0.92 ± 0.08	0.83 ± 0.03	0.67 ± 0.05	0.67 ± 0.05	0.74 ± 0.05	0.93 ± 0.04	0.83 ± 0.03	0.67 ± 0.06	0.67 ± 0.06	0.77	0.83	0.82	0.52	0.83	5.67	2/74	0.027
	Morgan (lv4) FP	0.76 ± 0.11	0.88 ± 0.08	0.82 ± 0.03	0.65 ± 0.06	0.65 ± 0.06	0.77 ± 0.06	0.85 ± 0.05	0.81 ± 0.03	0.62 ± 0.05	0.62 ± 0.05	0.71	0.84	0.82	0.49	0.82	6.17	0.74 ± 0.09	0.82 ± 0.08	0.78 ± 0.05	0.55 ± 0.1	0.55 ± 0.1	0.78 ± 0.05	0.85 ± 0.05	0.81 ± 0.03	0.63 ± 0.06	0.63 ± 0.06	0.82	0.86	0.85	0.6	0.83	7.69	4/74	0.054
	Layer FP	0.84 ± 0.1	0.74 ± 0.09	0.79 ± 0.08	0.59 ± 0.15	0.59 ± 0.15	0.83 ± 0.06	0.84 ± 0.08	0.83 ± 0.03	0.67 ± 0.06	0.67 ± 0.06	0.88	0.79	0.81	0.56	0.89	7.09	***0.89 ± 0.03***	***0.76 ± 0.17***	***0.83 ± 0.08***	***0.66 ± 0.14***	***0.66 ± 0.14***	***0.85 ± 0.06***	***0.88 ± 0.04***	***0.86 ± 0.03***	***0.73 ± 0.06***	***0.73 ± 0.06***	***0.9***	***0.9***	***0.9***	***0.7***	***0.9***	***5.64***	***0/74***	***####***
	MACCS FP	0.91 ± 0.05	0.76 ± 0.07	0.84 ± 0.04	0.68 ± 0.1	0.68 ± 0.1	0.85 ± 0.08	0.83 ± 0.06	0.84 ± 0.04	0.68 ± 0.07	0.68 ± 0.07	0.65	0.84	0.81	0.45	0.89	3.55	0.84 ± 0.16	0.87 ± 0.11	0.85 ± 0.06	0.72 ± 0.08	0.72 ± 0.08	0.82 ± 0.06	0.87 ± 0.05	0.85 ± 0.03	0.69 ± 0.07	0.69 ± 0.07	0.94	0.84	0.86	0.67	0.9	7.08	0/74	0.000
	Torsion FP	0.84 ± 0.12	0.86 ± 0.05	0.84 ± 0.07	0.7 ± 0.12	0.7 ± 0.12	0.78 ± 0.07	0.79 ± 0.05	0.79 ± 0.04	0.57 ± 0.07	0.57 ± 0.07	0.82	0.83	0.83	0.57	0.86	5.24	0.84 ± 0.1	0.8 ± 0.07	0.82 ± 0.06	0.63 ± 0.13	0.63 ± 0.13	0.81 ± 0.06	0.76 ± 0.06	0.78 ± 0.04	0.57 ± 0.07	0.57 ± 0.07	0.71	0.87	0.84	0.54	0.83	4.94	3/74	0.041
Cirrhosis	2D descriptor	0.82 ± 0.12	0.82 ± 0.05	0.82 ± 0.05	0.64 ± 0.1	0.64 ± 0.1	0.87 ± 0.05	0.87 ± 0.05	0.87 ± 0.03	0.75 ± 0.06	0.75 ± 0.06	0.79	0.91	0.89	0.65	0.89	7.76	0.75 ± 0.17	0.75 ± 0.12	0.76 ± 0.08	0.52 ± 0.16	0.52 ± 0.16	0.85 ± 0.08	0.89 ± 0.06	0.87 ± 0.04	0.75 ± 0.09	0.75 ± 0.09	0.79	0.84	0.83	0.53	0.81	4.12	2/84	0.024
	Atompair FP	0.79 ± 0.15	0.83 ± 0.06	0.81 ± 0.08	0.62 ± 0.14	0.62 ± 0.14	0.83 ± 0.06	0.84 ± 0.06	0.83 ± 0.03	0.67 ± 0.06	0.67 ± 0.06	0.79	0.86	0.85	0.55	0.82	6.25	0.86 ± 0.15	0.86 ± 0.05	0.86 ± 0.08	0.72 ± 0.16	0.72 ± 0.16	0.88 ± 0.06	0.89 ± 0.06	0.88 ± 0.04	0.77 ± 0.08	0.77 ± 0.08	0.86	0.86	0.86	0.61	0.9	5.99	1/84	0.012
	Avalon count FP	0.74 ± 0.12	0.8 ± 0.12	0.77 ± 0.1	0.54 ± 0.19	0.54 ± 0.19	0.9 ± 0.06	0.87 ± 0.07	0.89 ± 0.04	0.78 ± 0.07	0.78 ± 0.07	0.93	0.86	0.87	0.66	0.93	5.85	0.83 ± 0.08	0.84 ± 0.1	0.84 ± 0.06	0.68 ± 0.12	0.68 ± 0.12	0.85 ± 0.07	0.86 ± 0.06	0.85 ± 0.04	0.71 ± 0.08	0.71 ± 0.08	0.71	0.9	0.87	0.57	0.9	6.98	0/84	0.000
	Avalon FP	***0.87 ± 0.05***	***0.87 ± 0.09***	***0.87 ± 0.03***	***0.75 ± 0.07***	***0.75 ± 0.07***	***0.86 ± 0.05***	***0.91 ± 0.05***	***0.89 ± 0.03***	***0.78 ± 0.06***	***0.78 ± 0.06***	***0.9***	***0.9***	***0.9***	***0.7***	***1***	***7.98***	0.89 ± 0.1	0.83 ± 0.12	0.86 ± 0.06	0.72 ± 0.12	0.72 ± 0.12	0.89 ± 0.06	0.9 ± 0.05	0.9 ± 0.04	0.8 ± 0.07	0.8 ± 0.07	0.86	0.89	0.88	0.65	0.94	6.26	***0/84***	***####***
	Morgan (lv2) FP	0.83 ± 0.09	0.86 ± 0.12	0.85 ± 0.06	0.68 ± 0.13	0.68 ± 0.13	0.89 ± 0.05	0.82 ± 0.06	0.85 ± 0.03	0.71 ± 0.06	0.71 ± 0.06	0.93	0.87	0.88	0.68	0.95	9.26	0.84 ± 0.11	0.83 ± 0.08	0.85 ± 0.04	0.68 ± 0.08	0.68 ± 0.08	0.85 ± 0.06	0.88 ± 0.06	0.87 ± 0.03	0.74 ± 0.06	0.74 ± 0.06	0.71	0.91	0.88	0.6	0.81	5.6	0/84	0.000
	Morgan (lv3) FP	0.83 ± 0.06	0.85 ± 0.1	0.85 ± 0.06	0.69 ± 0.11	0.69 ± 0.11	0.87 ± 0.08	0.84 ± 0.06	0.85 ± 0.04	0.71 ± 0.08	0.71 ± 0.08	0.71	0.84	0.82	0.48	0.87	5.06	0.75 ± 0.19	0.93 ± 0.06	0.84 ± 0.07	0.7 ± 0.13	0.7 ± 0.13	0.85 ± 0.07	0.89 ± 0.06	0.87 ± 0.04	0.75 ± 0.07	0.75 ± 0.07	0.86	0.87	0.87	0.63	0.93	7.02	0/84	0.000
	Morgan (lv4) FP	0.92 ± 0.12	0.81 ± 0.15	0.86 ± 0.06	0.74 ± 0.1	0.74 ± 0.1	0.84 ± 0.06	0.88 ± 0.05	0.86 ± 0.03	0.73 ± 0.06	0.73 ± 0.06	0.86	0.86	0.86	0.61	0.86	8.82	0.89 ± 0.07	0.86 ± 0.07	0.87 ± 0.03	0.75 ± 0.07	0.75 ± 0.07	0.88 ± 0.06	0.9 ± 0.06	0.89 ± 0.04	0.78 ± 0.07	0.78 ± 0.07	0.86	0.94	0.93	0.76	0.96	6.14	0/84	0.000
	Layer FP	0.9 ± 0.02	0.88 ± 0.09	0.89 ± 0.05	0.78 ± 0.09	0.78 ± 0.09	0.91 ± 0.06	0.86 ± 0.05	0.89 ± 0.03	0.78 ± 0.07	0.78 ± 0.07	0.79	0.83	0.82	0.51	0.92	4.85	0.89 ± 0.04	0.86 ± 0.1	0.87 ± 0.05	0.74 ± 0.11	0.74 ± 0.11	0.91 ± 0.06	0.9 ± 0.06	0.91 ± 0.04	0.81 ± 0.08	0.81 ± 0.08	0.79	0.94	0.92	0.71	0.95	6.84	0/84	0.000
	MACCS FP	0.8 ± 0.08	0.94 ± 0.05	0.87 ± 0.06	0.75 ± 0.11	0.75 ± 0.11	0.86 ± 0.08	0.85 ± 0.08	0.86 ± 0.03	0.72 ± 0.06	0.72 ± 0.06	0.86	0.87	0.87	0.63	0.91	5.74	0.84 ± 0.09	0.88 ± 0.12	0.85 ± 0.07	0.7 ± 0.14	0.7 ± 0.14	0.87 ± 0.07	0.88 ± 0.08	0.88 ± 0.03	0.76 ± 0.05	0.76 ± 0.05	0.86	0.86	0.86	0.61	0.95	5.18	0/84	0.000
	Torsion FP	0.89 ± 0.06	0.82 ± 0.12	0.86 ± 0.08	0.72 ± 0.16	0.72 ± 0.16	0.86 ± 0.05	0.87 ± 0.05	0.86 ± 0.03	0.73 ± 0.05	0.73 ± 0.05	0.79	0.89	0.87	0.6	0.91	8.24	0.82 ± 0.09	0.83 ± 0.07	0.84 ± 0.07	0.66 ± 0.14	0.66 ± 0.14	0.86 ± 0.04	0.88 ± 0.05	0.87 ± 0.03	0.74 ± 0.06	0.74 ± 0.06	0.79	0.83	0.82	0.51	0.87	3.99	-	-
Hepatitis	2D descriptor	0.75 ± 0.11	0.84 ± 0.08	0.79 ± 0.07	0.59 ± 0.13	0.59 ± 0.13	0.79 ± 0.05	0.81 ± 0.04	0.8 ± 0.03	0.61 ± 0.05	0.61 ± 0.05	0.81	0.77	0.8	0.52	0.84	5.47	0.8 ± 0.11	0.78 ± 0.05	0.79 ± 0.07	0.58 ± 0.13	0.58 ± 0.13	0.85 ± 0.05	0.83 ± 0.05	0.84 ± 0.03	0.68 ± 0.06	0.68 ± 0.06	0.75	0.89	0.78	0.55	0.85	7.89	2/110	0.018
	Atompair FP	0.87 ± 0.03	0.7 ± 0.08	0.79 ± 0.05	0.58 ± 0.08	0.58 ± 0.08	0.82 ± 0.04	0.82 ± 0.04	0.82 ± 0.03	0.64 ± 0.05	0.64 ± 0.05	0.82	0.89	0.84	0.63	0.88	6.57	0.8 ± 0.07	0.76 ± 0.12	0.77 ± 0.03	0.56 ± 0.06	0.56 ± 0.06	0.83 ± 0.06	0.84 ± 0.05	0.84 ± 0.03	0.67 ± 0.07	0.67 ± 0.07	0.87	0.73	0.84	0.57	0.83	6.5	4/110	0.036
	Avalon count FP	0.76 ± 0.06	0.84 ± 0.07	0.79 ± 0.04	0.59 ± 0.08	0.59 ± 0.08	0.79 ± 0.05	0.84 ± 0.05	0.82 ± 0.03	0.63 ± 0.05	0.63 ± 0.05	0.92	0.81	0.89	0.71	0.92	9.64	0.75 ± 0.12	0.84 ± 0.08	0.8 ± 0.04	0.6 ± 0.08	0.6 ± 0.08	0.82 ± 0.04	0.85 ± 0.05	0.84 ± 0.03	0.67 ± 0.06	0.67 ± 0.06	0.83	0.69	0.8	0.49	0.82	4.68	3/110	0.027
	Avalon FP	0.82 ± 0.04	0.74 ± 0.17	0.79 ± 0.08	0.57 ± 0.18	0.57 ± 0.18	0.78 ± 0.06	0.77 ± 0.06	0.78 ± 0.03	0.56 ± 0.06	0.56 ± 0.06	0.81	0.77	0.8	0.52	0.84	6.24	0.71 ± 0.07	0.76 ± 0.07	0.73 ± 0.03	0.47 ± 0.05	0.47 ± 0.05	0.82 ± 0.06	0.81 ± 0.05	0.81 ± 0.03	0.63 ± 0.06	0.63 ± 0.06	0.81	0.62	0.76	0.4	0.8	6.45	0/110	0.000
	Morgan (lv2) FP	0.82 ± 0.1	0.8 ± 0.07	0.8 ± 0.04	0.61 ± 0.07	0.61 ± 0.07	0.76 ± 0.07	0.82 ± 0.08	0.79 ± 0.03	0.58 ± 0.06	0.58 ± 0.06	0.85	0.92	0.86	0.69	0.92	6.45	0.76 ± 0.08	0.86 ± 0.07	0.81 ± 0.03	0.63 ± 0.07	0.63 ± 0.07	0.75 ± 0.05	0.86 ± 0.04	0.8 ± 0.02	0.61 ± 0.05	0.61 ± 0.05	0.77	0.81	0.78	0.51	0.87	11.91	0/110	0.000
	Morgan (lv3) FP	0.71 ± 0.15	0.73 ± 0.18	0.71 ± 0.08	0.46 ± 0.17	0.46 ± 0.17	0.71 ± 0.08	0.75 ± 0.07	0.73 ± 0.03	0.47 ± 0.06	0.47 ± 0.06	0.74	0.73	0.74	0.41	0.76	4.93	0.73 ± 0.13	0.73 ± 0.1	0.73 ± 0.08	0.47 ± 0.16	0.47 ± 0.16	0.7 ± 0.06	0.82 ± 0.05	0.76 ± 0.03	0.52 ± 0.05	0.52 ± 0.05	0.67	0.73	0.68	0.34	0.76	3.88	8/110	0.073
	Morgan (lv4) FP	0.74 ± 0.07	0.78 ± 0.04	0.76 ± 0.04	0.52 ± 0.07	0.52 ± 0.07	0.71 ± 0.04	0.84 ± 0.05	0.78 ± 0.02	0.56 ± 0.05	0.56 ± 0.05	0.81	0.73	0.79	0.49	0.83	5.52	0.76 ± 0.06	0.78 ± 0.07	0.77 ± 0.04	0.54 ± 0.08	0.54 ± 0.08	0.85 ± 0.05	0.84 ± 0.04	0.84 ± 0.03	0.68 ± 0.05	0.68 ± 0.05	0.81	0.73	0.79	0.49	0.86	7.49	1/110	0.009
	Layer FP	0.79 ± 0.04	0.69 ± 0.13	0.74 ± 0.07	0.48 ± 0.14	0.48 ± 0.14	0.79 ± 0.06	0.78 ± 0.06	0.79 ± 0.03	0.58 ± 0.05	0.58 ± 0.05	0.82	0.73	0.8	0.51	0.77	8.05	0.78 ± 0.03	0.73 ± 0.13	0.76 ± 0.06	0.52 ± 0.12	0.52 ± 0.12	0.79 ± 0.05	0.78 ± 0.05	0.79 ± 0.03	0.58 ± 0.05	0.58 ± 0.05	0.81	0.69	0.78	0.46	0.8	5.93	0/110	0.000
	MACCS FP	0.76 ± 0.08	0.81 ± 0.1	0.79 ± 0.08	0.58 ± 0.15	0.58 ± 0.15	0.79 ± 0.06	0.83 ± 0.05	0.81 ± 0.03	0.63 ± 0.06	0.63 ± 0.06	0.82	0.73	0.8	0.51	0.82	7.42	***0.79 ± 0.06***	***0.85 ± 0.05***	***0.82 ± 0.04***	***0.64 ± 0.08***	***0.64 ± 0.08***	***0.8 ± 0.05***	***0.87 ± 0.05***	***0.84 ± 0.03***	***0.68 ± 0.06***	***0.68 ± 0.06***	***0.9***	***0.9***	***0.9***	***0.7***	***0.9***	***10.9***	***0/110***	***####***
	Torsion FP	0.72 ± 0.06	0.77 ± 0.08	0.75 ± 0.07	0.49 ± 0.14	0.49 ± 0.14	0.72 ± 0.05	0.82 ± 0.05	0.77 ± 0.03	0.55 ± 0.05	0.55 ± 0.05	0.75	0.85	0.77	0.52	0.85	4.4	0.72 ± 0.06	0.78 ± 0.05	0.75 ± 0.05	0.5 ± 0.09	0.5 ± 0.09	0.77 ± 0.05	0.82 ± 0.05	0.8 ± 0.02	0.6 ± 0.04	0.6 ± 0.04	0.74	0.81	0.76	0.48	0.77	7.54	11/110	0.100
Steatosis	2D descriptor	0.91 ± 0.09	0.8 ± 0.07	0.85 ± 0.04	0.71 ± 0.08	0.71 ± 0.08	0.89 ± 0.06	0.82 ± 0.05	0.86 ± 0.03	0.72 ± 0.07	0.72 ± 0.07	0.9	0.84	0.86	0.67	0.88	6.01	0.86 ± 0.1	0.82 ± 0.08	0.83 ± 0.04	0.67 ± 0.08	0.67 ± 0.08	0.84 ± 0.06	0.87 ± 0.05	0.86 ± 0.03	0.72 ± 0.06	0.72 ± 0.06	0.8	0.84	0.83	0.59	0.87	5.9	1/65	0.015
	Atompair FP	0.82 ± 0.05	0.72 ± 0.03	0.77 ± 0.03	0.54 ± 0.05	0.54 ± 0.05	0.87 ± 0.06	0.79 ± 0.06	0.84 ± 0.03	0.68 ± 0.07	0.68 ± 0.07	0.85	0.75	0.77	0.52	0.86	3.91	0.83 ± 0.08	0.82 ± 0.11	0.84 ± 0.07	0.65 ± 0.14	0.65 ± 0.14	0.85 ± 0.06	0.88 ± 0.05	0.87 ± 0.03	0.74 ± 0.06	0.74 ± 0.06	0.75	0.84	0.82	0.55	0.87	6.68	4/65	0.062
	Avalon count FP	0.78 ± 0.05	0.84 ± 0.11	0.81 ± 0.08	0.61 ± 0.16	0.61 ± 0.16	0.85 ± 0.06	0.85 ± 0.08	0.85 ± 0.04	0.7 ± 0.07	0.7 ± 0.07	0.9	0.84	0.86	0.67	0.91	8.13	0.83 ± 0.14	0.78 ± 0.14	0.79 ± 0.05	0.62 ± 0.07	0.62 ± 0.07	0.86 ± 0.06	0.78 ± 0.06	0.82 ± 0.04	0.64 ± 0.08	0.64 ± 0.08	0.85	0.83	0.83	0.61	0.85	4.42	3/65	0.046
	Avalon FP	0.92 ± 0.06	0.72 ± 0.17	0.83 ± 0.1	0.66 ± 0.21	0.66 ± 0.21	0.92 ± 0.06	0.74 ± 0.06	0.84 ± 0.03	0.68 ± 0.06	0.68 ± 0.06	0.95	0.78	0.82	0.64	0.9	4.23	***0.86 ± 0.06***	***0.83 ± 0.05***	***0.84 ± 0.04***	***0.69 ± 0.07***	***0.69 ± 0.07***	***0.86 ± 0.06***	***0.82 ± 0.07***	***0.84 ± 0.04***	***0.69 ± 0.07***	***0.69 ± 0.07***	***0.9***	***0.8***	***0.8***	***0.6***	***0.9***	***7.42***	***0/65***	***####***
	Morgan (lv2) FP	0.71 ± 0.15	0.9 ± 0.11	0.79 ± 0.13	0.6 ± 0.25	0.6 ± 0.25	0.73 ± 0.06	0.83 ± 0.08	0.78 ± 0.04	0.57 ± 0.07	0.57 ± 0.07	0.75	0.87	0.84	0.6	0.82	10.84	0.77 ± 0.09	0.83 ± 0.07	0.79 ± 0.04	0.6 ± 0.07	0.6 ± 0.07	0.76 ± 0.05	0.82 ± 0.07	0.79 ± 0.03	0.58 ± 0.06	0.58 ± 0.06	0.8	0.86	0.84	0.61	0.8	4.74	4/65	0.062
	Morgan (lv3) FP	0.76 ± 0.05	0.8 ± 0.11	0.78 ± 0.03	0.56 ± 0.07	0.56 ± 0.07	0.8 ± 0.06	0.73 ± 0.07	0.77 ± 0.03	0.53 ± 0.06	0.53 ± 0.06	0.85	0.67	0.71	0.44	0.87	5.74	0.75 ± 0.13	0.74 ± 0.08	0.76 ± 0.09	0.5 ± 0.17	0.5 ± 0.17	0.82 ± 0.05	0.73 ± 0.09	0.78 ± 0.03	0.56 ± 0.07	0.56 ± 0.07	0.85	0.76	0.78	0.54	0.89	6.8	1/65	0.015
	Morgan (lv4) FP	0.76 ± 0.11	0.76 ± 0.12	0.76 ± 0.08	0.52 ± 0.17	0.52 ± 0.17	0.79 ± 0.07	0.77 ± 0.08	0.78 ± 0.04	0.56 ± 0.08	0.56 ± 0.08	0.85	0.84	0.84	0.63	0.84	5.46	0.74 ± 0.09	0.87 ± 0.07	0.79 ± 0.05	0.6 ± 0.07	0.6 ± 0.07	0.8 ± 0.04	0.82 ± 0.05	0.81 ± 0.03	0.62 ± 0.05	0.62 ± 0.05	0.65	0.81	0.77	0.43	0.75	2.89	1/65	0.015
	Layer FP	0.88 ± 0.1	0.78 ± 0.15	0.83 ± 0.09	0.67 ± 0.16	0.67 ± 0.16	0.92 ± 0.04	0.72 ± 0.07	0.83 ± 0.04	0.66 ± 0.07	0.66 ± 0.07	0.8	0.79	0.8	0.53	0.73	4.11	0.85 ± 0.07	0.82 ± 0.1	0.83 ± 0.07	0.66 ± 0.14	0.66 ± 0.14	0.91 ± 0.05	0.83 ± 0.06	0.88 ± 0.04	0.75 ± 0.07	0.75 ± 0.07	0.75	0.84	0.82	0.55	0.87	4.92	0/65	0.000
	MACCS FP	0.81 ± 0.14	0.82 ± 0.12	0.82 ± 0.09	0.64 ± 0.17	0.64 ± 0.17	0.88 ± 0.05	0.81 ± 0.06	0.85 ± 0.04	0.7 ± 0.08	0.7 ± 0.08	0.85	0.78	0.8	0.56	0.85	5.92	0.81 ± 0.1	0.83 ± 0.13	0.83 ± 0.09	0.64 ± 0.2	0.64 ± 0.2	0.89 ± 0.05	0.88 ± 0.04	0.88 ± 0.03	0.77 ± 0.05	0.77 ± 0.05	0.75	0.86	0.83	0.57	0.87	3.89	0/65	0.000
	Torsion FP	0.8 ± 0.08	0.83 ± 0.11	0.82 ± 0.04	0.64 ± 0.08	0.64 ± 0.08	0.81 ± 0.07	0.82 ± 0.07	0.81 ± 0.03	0.63 ± 0.05	0.63 ± 0.05	0.95	0.81	0.84	0.67	0.91	6.99	0.8 ± 0.14	0.89 ± 0.09	0.84 ± 0.05	0.7 ± 0.08	0.7 ± 0.08	0.86 ± 0.05	0.9 ± 0.05	0.88 ± 0.03	0.76 ± 0.05	0.76 ± 0.05	0.9	0.91	0.9	0.76	0.87	9.56	7/65	0.108

*AD analysis was failed in cirrhosis data set with torsion FP since the descriptor matrix was a singular matrix. Bolded and italic texts highlighted best performance among models.

#### Drug Based Models

In every model development process, randomization tests were applied to avoid a situation where correlation between endpoint and structural features was merely the result of coincidence. In DICH data set, most number of models failed to pass randomization test [SVM: Avalon count FP, Morgan (lv3) FP, Layer FP, and MACCS FP and RF: 2D descriptor and Morgan (lv4) FP]. Second most number of models failed in randomization test for DIS data set (SVM: 2D descriptor and Avalon count FP, and RF: Avalon count FP, Morgan (lv2) FP, and MACCS FP). In DIH data set, only two models failed in randomization test (SVM: MACCS FP, and RF: layer FP). In DIC data set, only one model failed to pass randomization test [SVM: Morgan (lv2) FP]. In terms of ACC and MCC from the external test set, models with atom-pair FP achieved the best performance in all drug data sets with a relatively high z score, and the second best models were those developed with topological-torsion FP.

Feature spaces represented by each FP and 2D descriptor were examined by principal component analysis (PCA) to project the N-dimension of the feature space into 2D space. This analysis showed that uneven distribution of data points was found throughout the space when drugs were represented by atom-pair FP (**Figure S2**) and topological torsion FP (**Figure S10**). The ADs of the models were visualized based on the leverage and molecular weight range of the drugs. According to the leverage of atom-pair FP and topological-torsion FP data sets, several data points were out of the domain in every data set (**Figures S22** and **S30**) while Avalon FP (**Figure S24**), layer FP (**Figure S28**), and MACCS FP (**Figure S29**) models didn't have any data points that exceeded the warning leverage.

The prediction results of the models were reliable when the inputted data point was included in the AD of the model; therefore, the AD of the models should be considered together with their accuracy when the best model is selected. In this study, performance in the external test set, randomization test results, and AD analysis were considered in selection of the best models. In DICH, MACCS FP model (RF) was considered the best model (external test ACC: 70%, Z-score: 3.14, AD: **Figure S29A**). In DIC, layer FP model (RF) was best (external test ACC: 90%, Z-score: 4.23, AD: **Figure S28B**). In DIH, 2D descriptor model (SVM) was best (external test ACC: 83%, Z-score: 5.06, AD: **Figure S21C**). In DIS, layer FP model (SVM) was best (external test ACC: 85%, Z-score: 5.10, AD: **Figure S28D**).

#### Drug Metabolite Based Models

In terms of ACC and MCC from the external test set, the best models were developed with Morgan (lv3) FP in DICH (SVM), Morgan (lv4) FP in DIC (RF), Morgan (lv2) FP in DIH (SVM), and topological-torsion FP in DIS (RF). In accuracy comparison, it was found that 63.8% of the models developed with drug metabolite data sets achieved higher accuracy compared to that developed with drug data sets (51 models out of 80 models). Moreover, only one model was failed in randomization test in all metabolite data sets (SVM: Avalon count FP). Due to the lack of DILI-labels on drug metabolites, the metabolites were labeled according to their parent drugs' DILI label based on the assumption that metabolites from DILI-positive drugs cause DILI and those from DILI-negatives do not. Improvement of the general performance of the model and randomization test results showed that assumption for the DILI labeling of metabolites was reasonable; however, it is still possible that models for drug metabolites contain a certain degree of structural noise introduced by the ambiguous DILI labeling of metabolites, particularly for DILI-positive metabolites since all metabolites were prepared according to the assumption. DILI-negative metabolites were composed of DILI-negative drugs and metabolites of DILI-negative drugs; hence, the correlation between structural features and endpoint is potentially not solely based on drug metabolite chemical space.

Feature space visualization was performed for drug metabolite data sets. In general, data distribution of the feature space was similar between drug data sets and drug metabolite data sets, except Morgan FPs where more outliers were found in feature space generated from drug metabolite sets (**Figures S15–S17**). In AD analysis of the drug metabolite set, models developed with Avalon FP (**Figure S34**), layer FP (**Figure S38**, and MACCS FP (**Figure S39**) showed that no data points exceeded the warning leverage.

Based on the prediction performance in the external test set, randomization test results, and AD analysis, the best models for drug metabolites were selected. AD of the models was considered more important than accuracy in model selection since models with narrow AD cannot be applied for prediction of heterogeneous structures. In cases where accuracy in the external test was equal, MCC was used to evaluate model performance. In DICH, the layer FP model (RF) was best (external test ACC: 86%, Z-score: 5.64, AD: **Figure S38A**). In DIC, the Avalon FP model (SVM) was best (external test ACC: 88%, Z-score: 7.98, AD: **Figure S34B**). In DIH, the MACCS FP model (RF) was best (external test ACC: 86%, Z-score: 10.85, AD: **Figure S39C**). In DIS, the Avalon FP model (RF) was best (external test ACC: 83%, Z-score: 7.42, AD: **Figure S34D**).

### Privileged Structure Analysis for Drug-Induced Liver Injury

Privileged substructures, which were more frequent in DILI-positive sets, were found using Morgan FP in level 2 based on the number of substructures and their frequency as calculated with equation 10. Privileged substructures for drugs are listed in **Tables S2–S5** and for drug metabolites in **Tables S6–S9**. Throughout every data set, including drugs and drug metabolites, the most commonly selected privileged substructure's SMARTS pattern was [#6]-[#6](-[#6])-[#6]-[#6](-[#6])-[#6] whose frequency (F) was relatively high for DILI-positive in all data sets except DIC of the drug data set. Privileged substructures that are sensitive to drug metabolism were also analyzed by comparing variations of the number of substructures between DILI-positive and DILI-negative drugs and drug metabolites (**Tables S10–S13**). Two privileged structures, [#6]-[#6]-1=[#6]-[#6]=[#6]-[#6]=[#6]-1 and [#8]-c(:c): in simplified molecular-input line-entry system arbitrary target specification (SMARTS) pattern, were increased in DILI-positive drug metabolite sets compared to that of drug sets while the number of substructures, [#6]-[#7] in SMARTS pattern, was relatively decreased in DILI-positive drug metabolite sets compared to drug sets and slightly increased in DILI-negative drug metabolites sets compared drug sets in all data sets. Substructures of the SMARTS patterns are available in **Table 4**. In DILI, drugs often damage hepatocytes through their reactive metabolites; therefore, privileged substructures were derived by comparing DILI-positive and DILI-negative sets and also comparing drug and drug metabolite data sets to consider metabolic activation of compounds. Even though privileged substructures were suggested based on cholestasis, cirrhosis, hepatitis, and steatosis data for drugs and drug metabolites, SAs based on the privileged substructures suggested in this work may have limited predictability for DILI since the substructures were obtained from small data sets, and uncertainty in drug metabolite labeling may introduce a certain degree of structural noise in the analysis. These substructures in **Tables S1–S12** may still need further exploration to confirm their predictability on DILI in SAs.

**Table 4:** Commonly increased and decreased substructures due to drug metabolism in all data sets.

Structure	V_N,__DICH_	V_F,__DICH_	V_N,__DIC_	V_F,__DIC_	V_N,__DIH_	V_F,__DIH_	V_N,__DIS_	V_F,__DIS_	Daylight SMARTS
	Increased in DILI-positive drug metabolites compared to DILI-positive drugs								
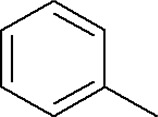	16	0.392	17	0.347	39	0.203	29	0.309	[#6]-[#6]-1=[#6]-[#6]=[#6]-[#6]=[#6]-1
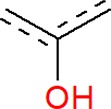	20	0.626	17	0.585	35	0.371	26	0.781	[#8]-c(:c):c
Decreased in DILI-positive drug metabolites compared to DILI-positive drugs								
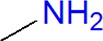	-11	-0.653	-10	-0.83	-22	-0.431	-7	-0.514	[#6]-[#7]

*V_N_, variation of the number of fragments in DILI-positive. It is calculated by substracting the number of fragment in DILI-positive drug sets from that in DILI-positive drug metabolite data sets.

*V_F_, variation of the frequency of fragments in DILI-positive. It is calculated by substracting the frequency of fragment in DILI-positive drug sets from that in DILI-positive drug metabolite data sets.

*DICH, drug-induced cholestasis; DIC, drug-induced cirrhosis; DIH, drug-induced hepatitis; DIS, drug-induced steatosis.

## Conclusion

In the current study, binary classification models were developed to predict four types of DILI; cholestasis, cirrhosis, hepatitis, and steatosis. 2D fingerprints and molecular descriptors were calculated from drugs and their metabolite structures to find significant structural features from both chemical spaces since DILI is caused by drugs or their reactive metabolites. Due to uncertainty in DILI labeling, we curated the DILI label on drugs by integrating DILIrank labels and post-market reports on drugs. Drug metabolites were labeled according to the label of their parent drugs based on the assumption that drug metabolites produced from DILI-positive drugs also caused DILI and drug metabolites from DILI-negative drugs did not. Models were developed and analyzed according to OECD guidelines for QSAR validation. Sufficient performances were observed from the models in internal and external validation results. In particular, improved performance in models built with drug metabolite sets implied that consideration of drug metabolism was significant in DILI prediction. Privileged substructure analysis identified frequent substructures in DILI-positive sets and drug metabolite sets that could be responsible for DILI induction or metabolic activation of drugs.

## Data Availability Statement

The datasets analyzed in this article are not publicly available. Requests to access the datasets should be directed to HS, hyunkil.shin@kitox.re.kr.

## Author Contributions

HS designed the study, which was validated by SY. TP collected drug metabolite structure and preprocessed data sets. DP analyzed chemical space, feature space, and privileged structures. Model development and validation, and applicability domain analysis were conducted by HS and M-GK. Manuscript was prepared and edited by HS, M-GK, DP, TP, and SY. All the authors approved the final version of the manuscript.

## Conflict of Interest

The authors declare that the research was conducted in the absence of any commercial or financial relationships that could be construed as a potential conflict of interest.
