# Supplementary material for: Development of Prediction Models for Drug-Induced Cholestasis, Cirrhosis, Hepatitis, and Steatosis Based on Drug and Drug Metabolite Structures
Source: Front Pharmacol. 2020 Feb 14;11:67. doi: 10.3389/fphar.2020.00067 (PMC7034408; doi:10.3389/fphar.2020.00067)
Supplement: Supplementary file 1 [file DataSheet_1.docx]

Supplementary Figures

# Contents

Figure S1~10. Feature space projection with PCA for drugs

Figure S11~20. Feature space projection with PCA for drug metabolites

Figure S21~30. Leverage analysis to define AD of drug structure-based models

Figure S31~40. Leverage analysis to define AD of drug metabolite structure-based models

*PCA: principal component analysis

*AD: Applicability domain

# Feature space projection with PCA for drugs


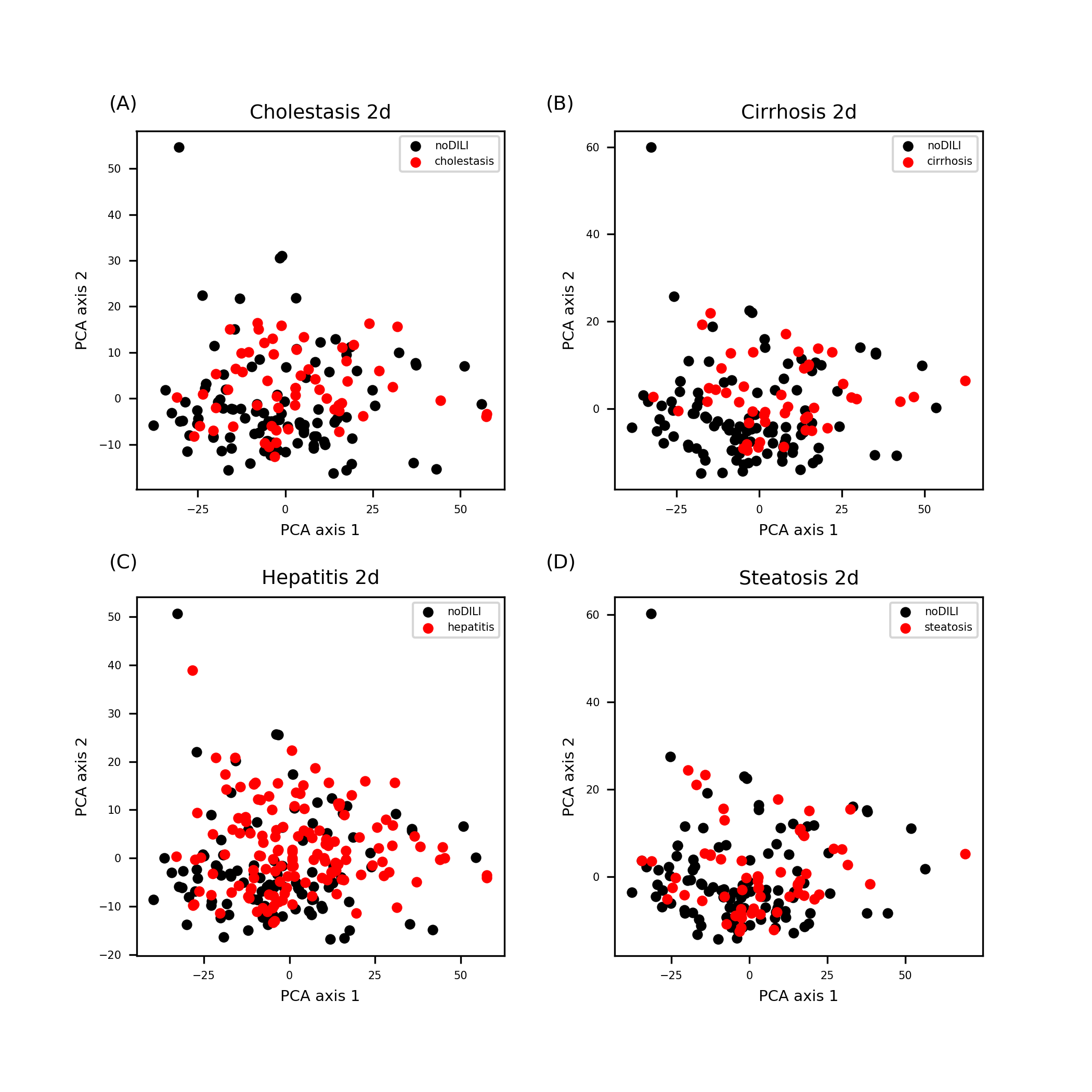


Figure S1. 2D descriptor feature space for drug data sets. Data points were evenly distributed throughout the feature space except one or two data points on upper left in the space.


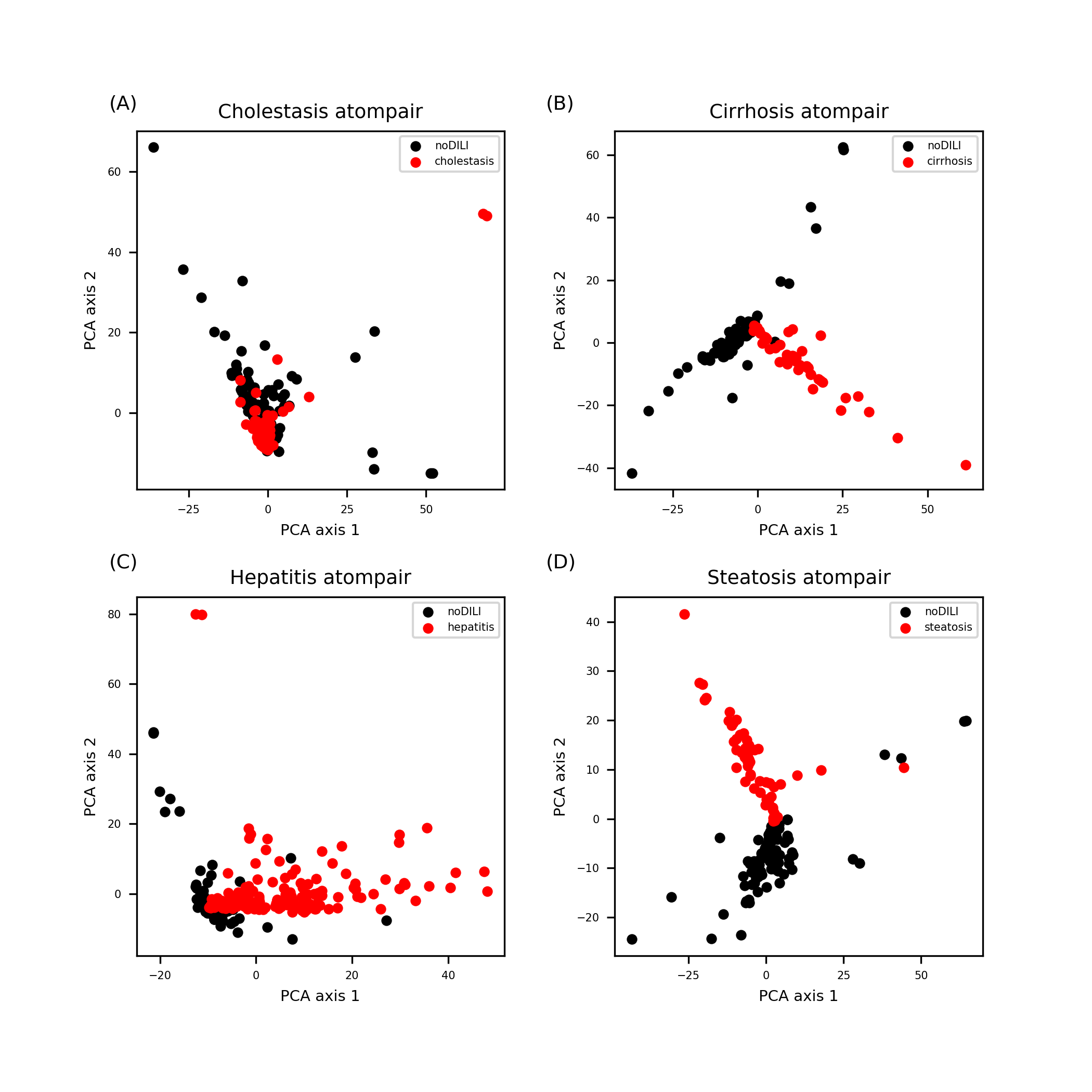


Figure S2. Atom-pair FP feature space for drug data sets. Highly uneven distributions were found for all feature spaces.


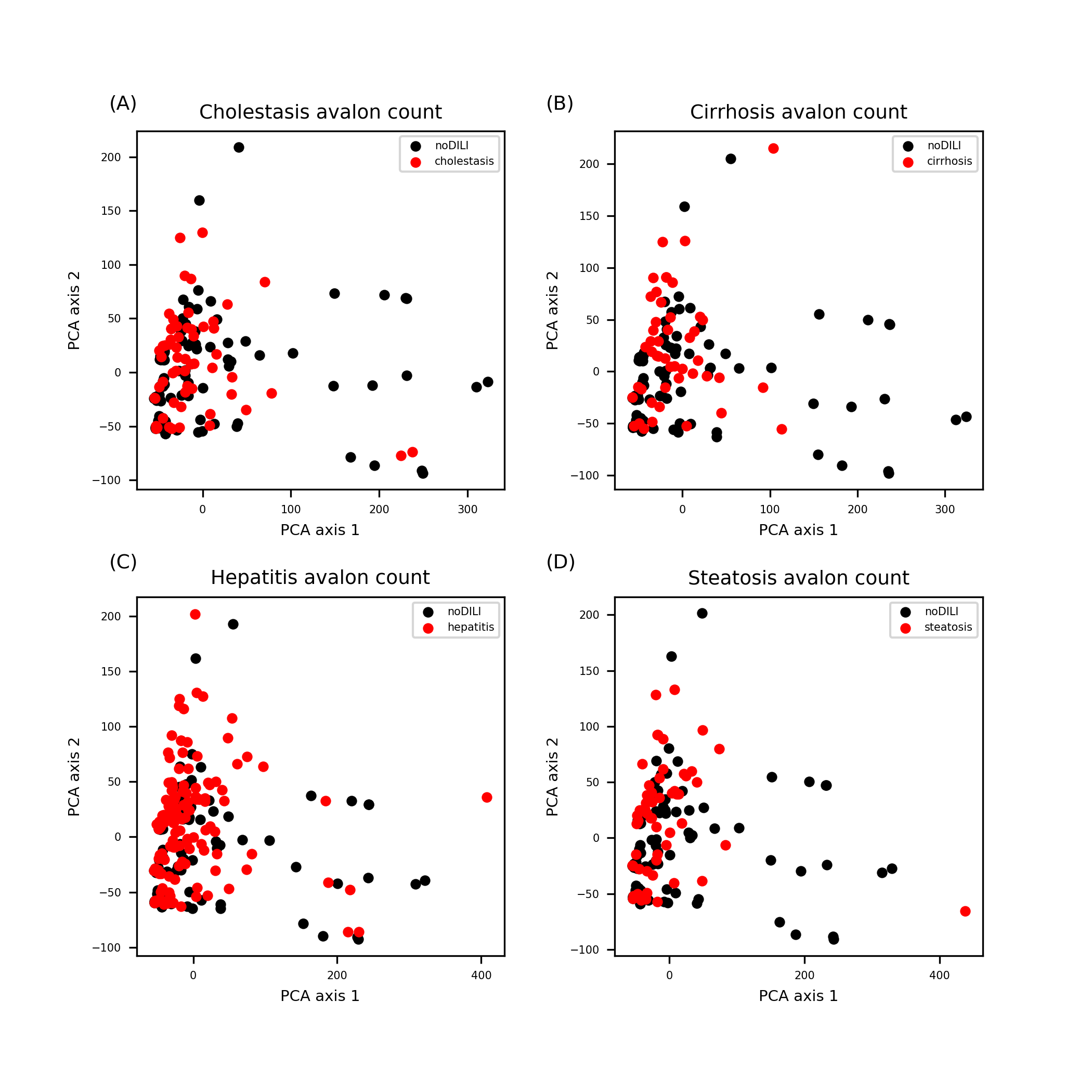


Figure S3. Avalon count FP feature space for drug data sets. Data points were clustered around a certain point (coordinate: 0,0) in the feature space with several data points scattered away from the cluster.


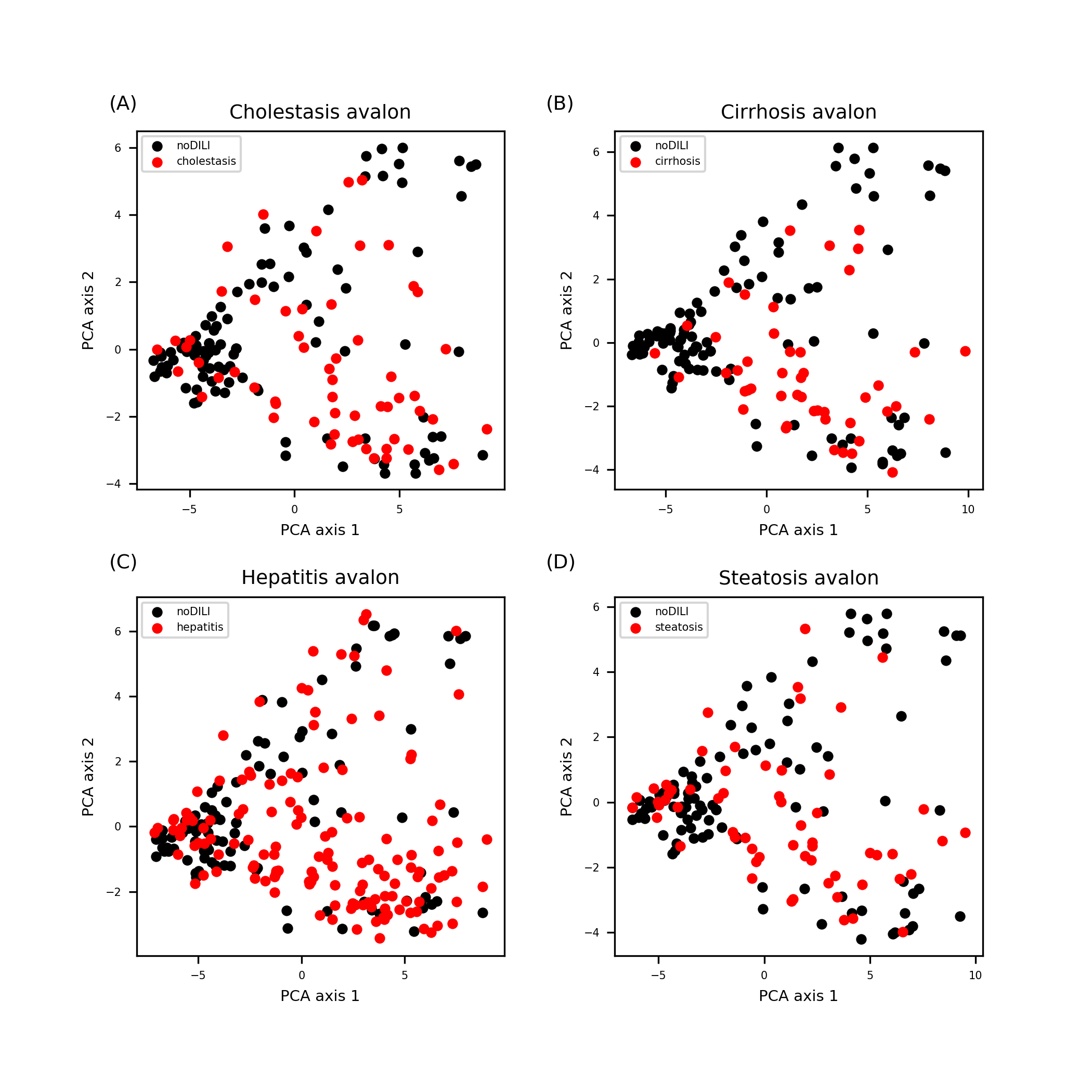


Figure S4. Avalon FP feature space for drug data sets. Although data cluster was formed around a certain point (coordinate: -5,0), most of data points were evenly distributed throughout the feature space.


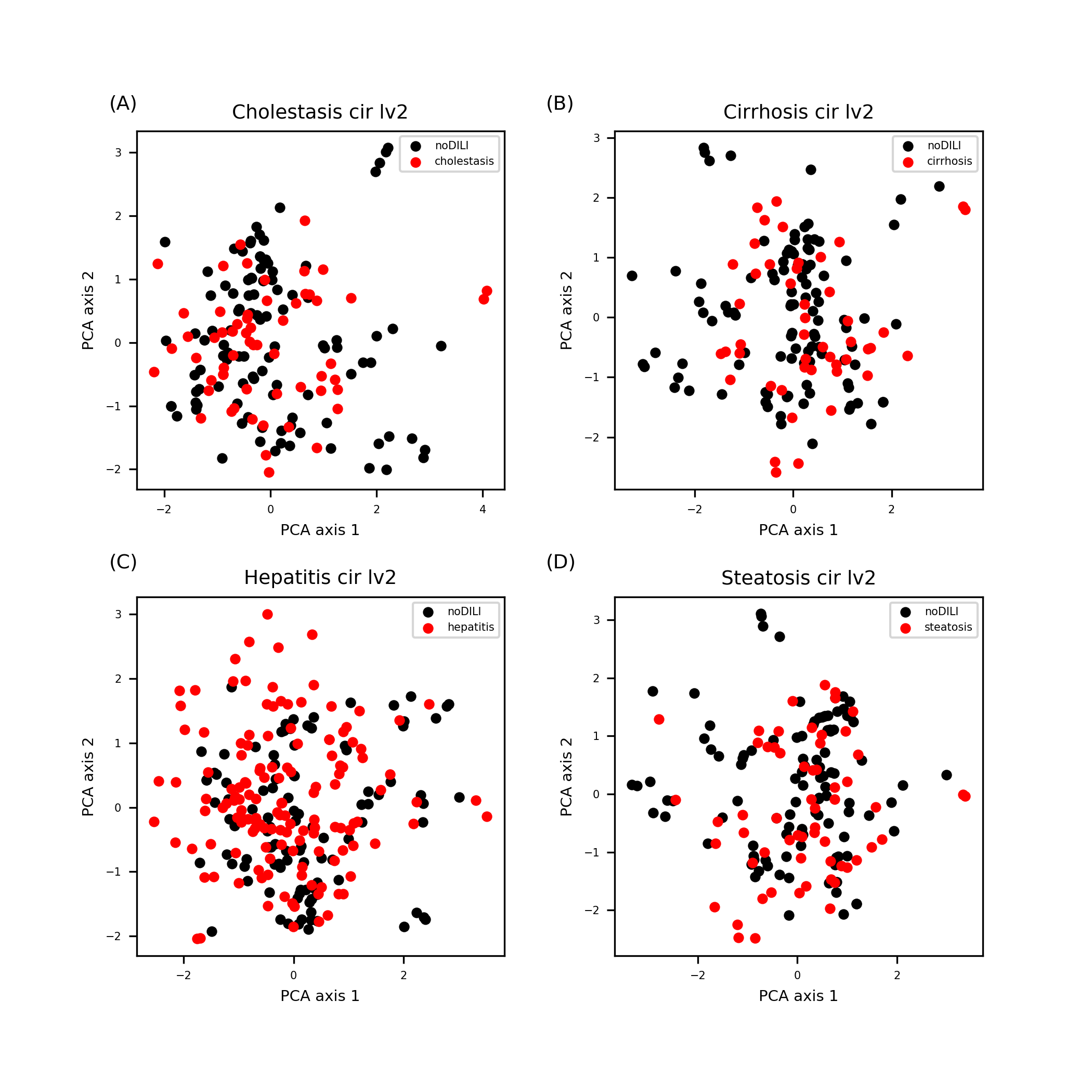


Figure S5. Morgan FP (level 2) feature space for drug data sets. Data points were evenly distributed throughout the feature space.


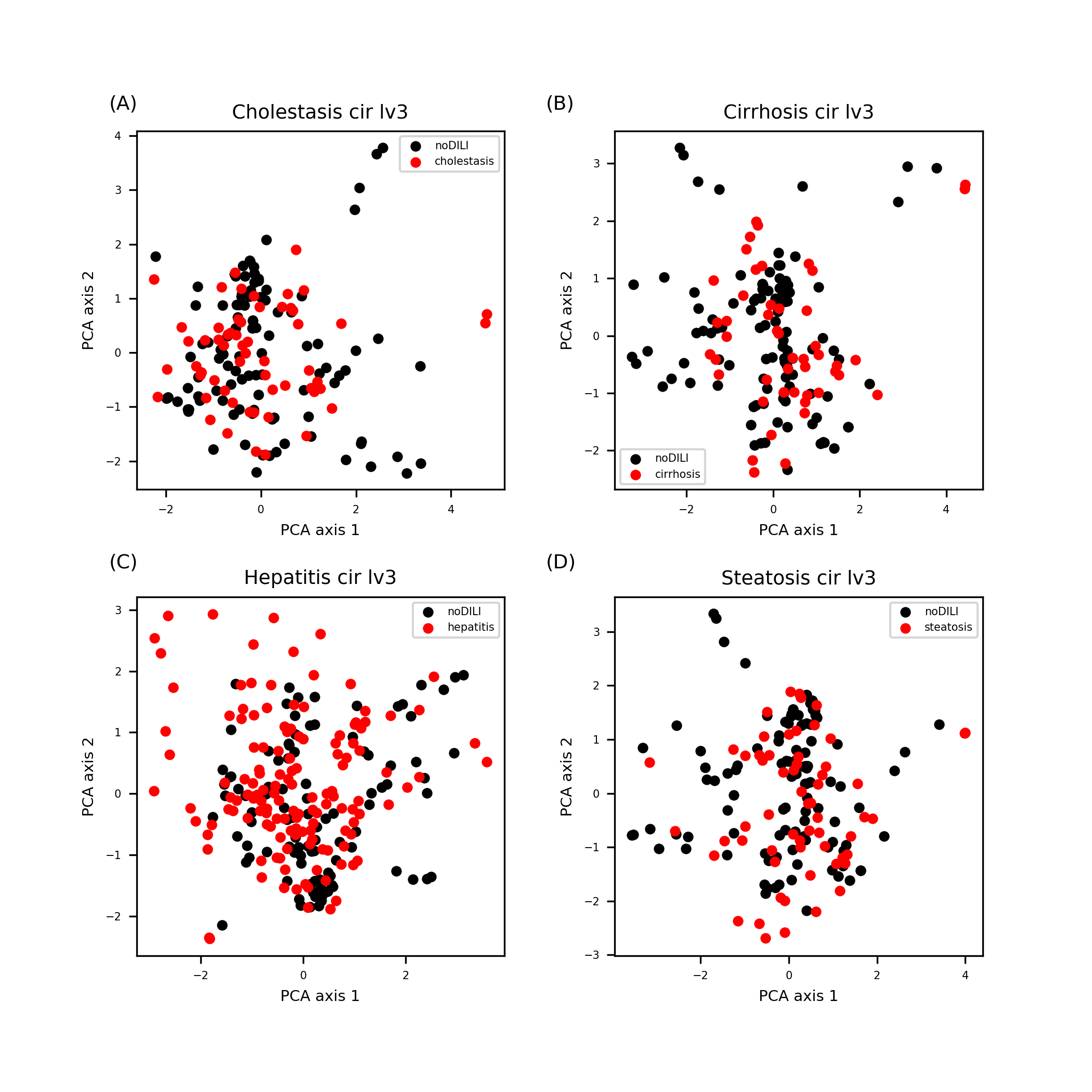


Figure S6. Morgan FP (level 3) feature space for drug data sets. Data points were evenly distributed throughout the feature space.


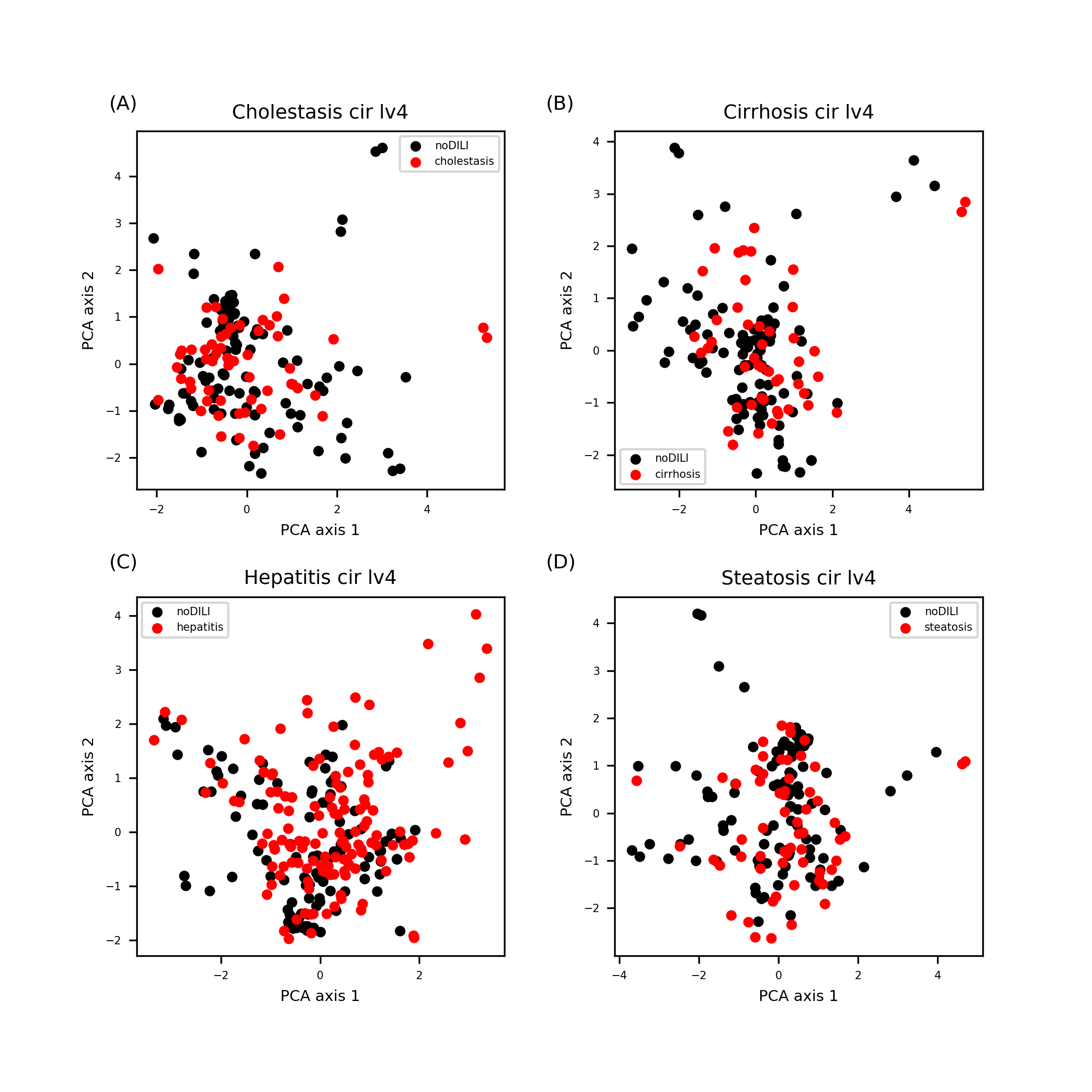


Figure S7. Morgan FP (level 4) feature space for drug data sets. Data points were evenly distributed throughout the feature space.


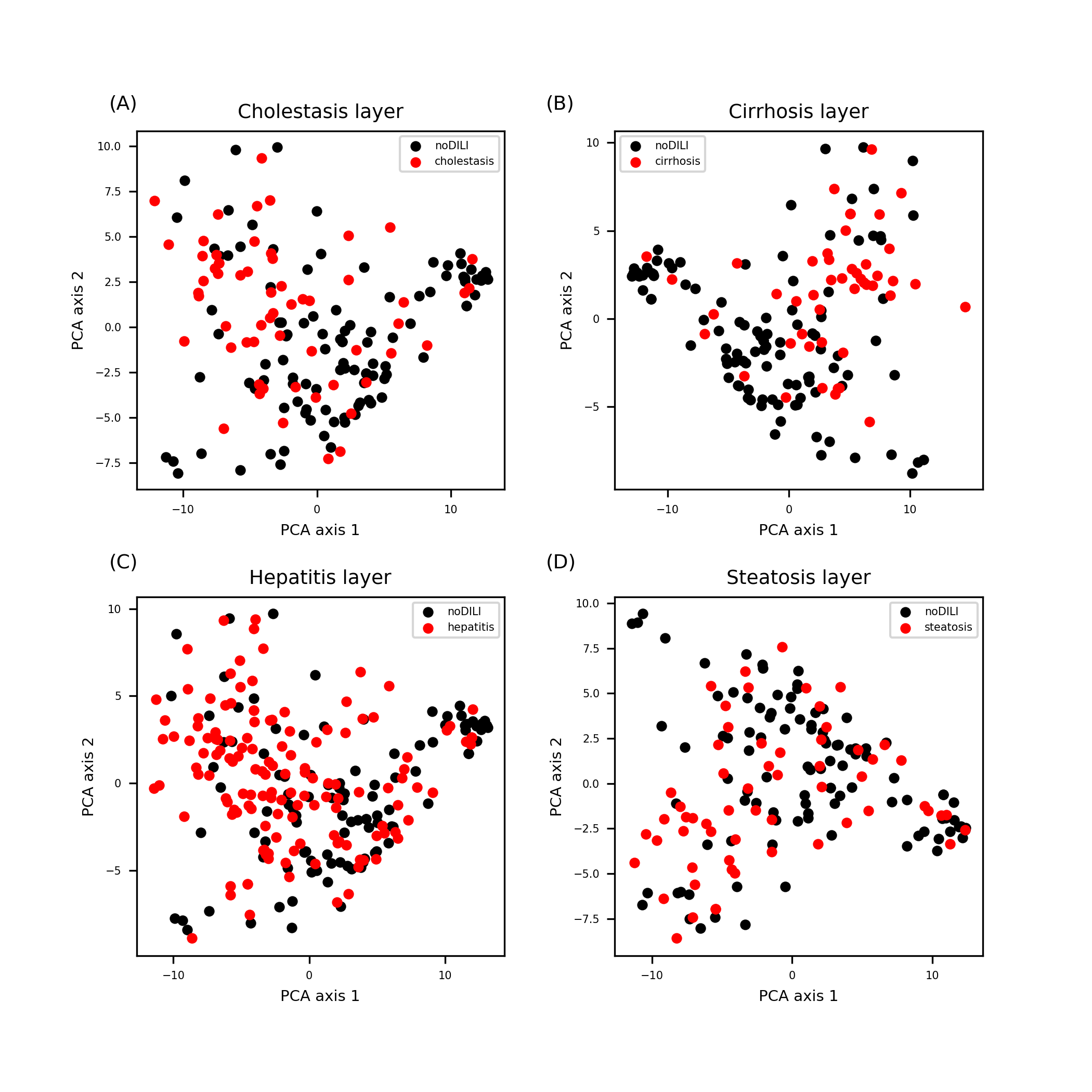


Figure S8. Layer FP feature space for drug data sets. Data points were evenly distributed throughout the feature space.


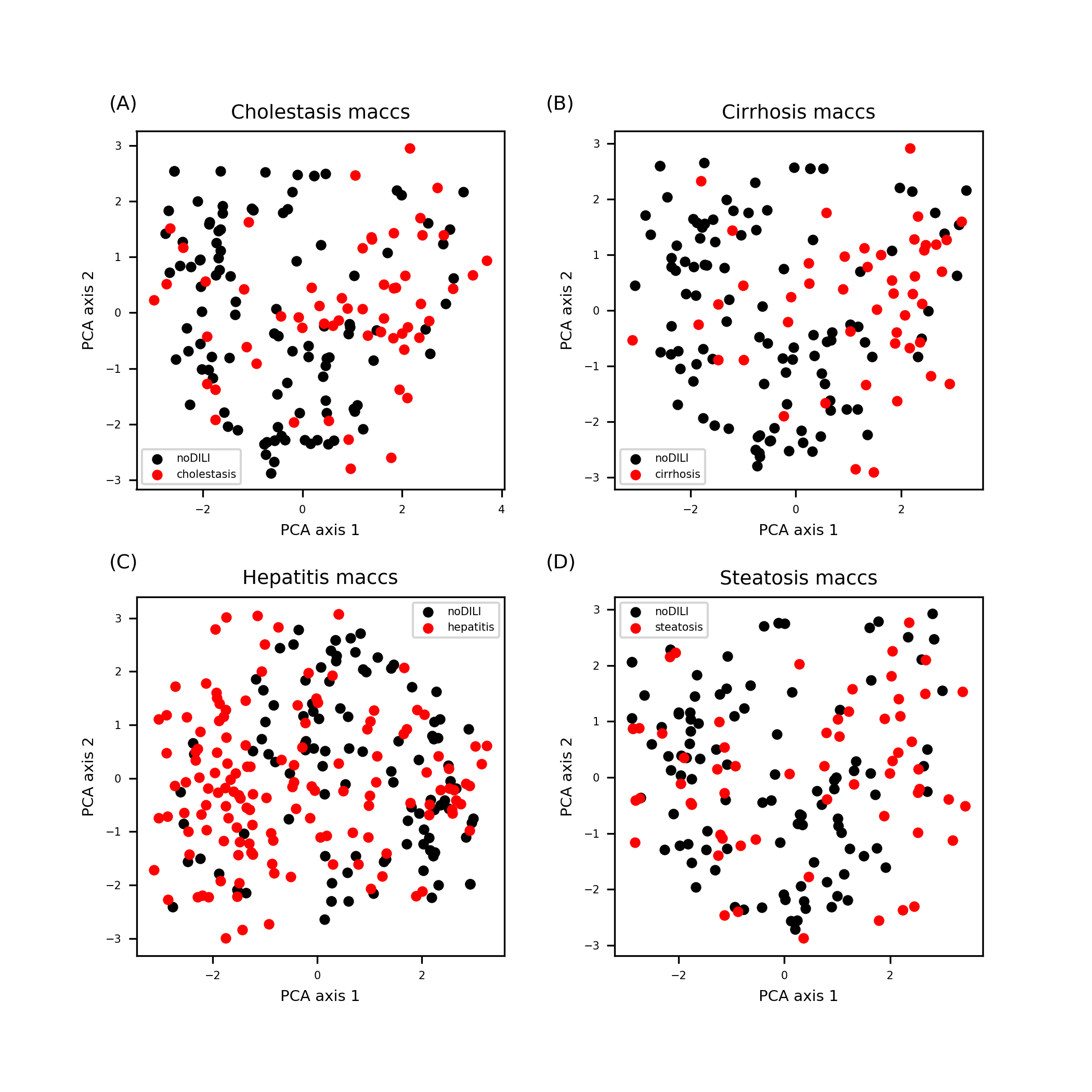


Figure S9. MACCS FP feature space for drug data sets. Data points were evenly distributed throughout the feature space.


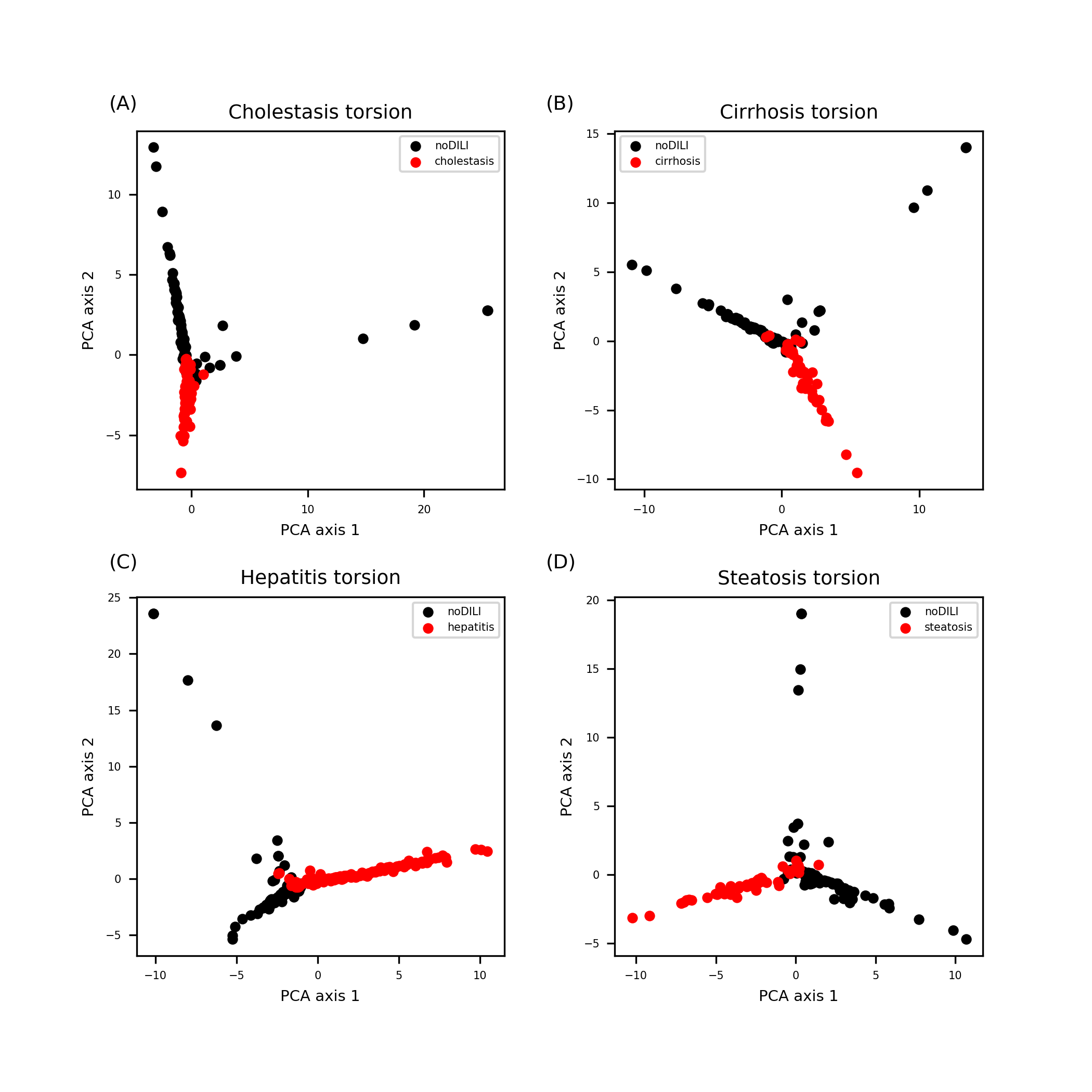


Figure S10. Topological-torsion FP feature space for drug data sets. Highly uneven distributions were found for all feature spaces.

# Feature space projection with PCA for drug metabolites


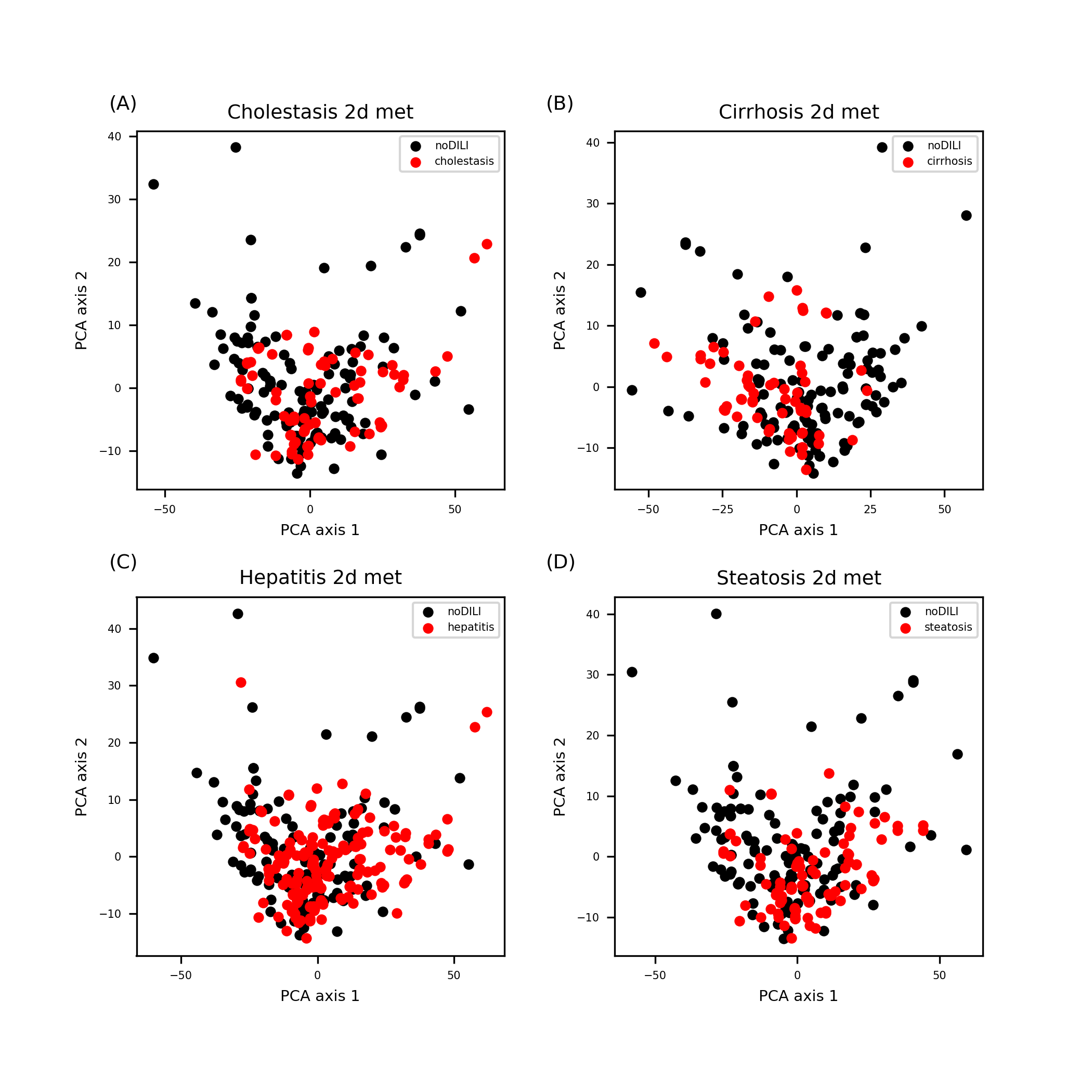


Figure S11. 2D descriptor feature space for drug metabolite data sets. Data points were evenly distributed except several data points found in upside of the feature space.


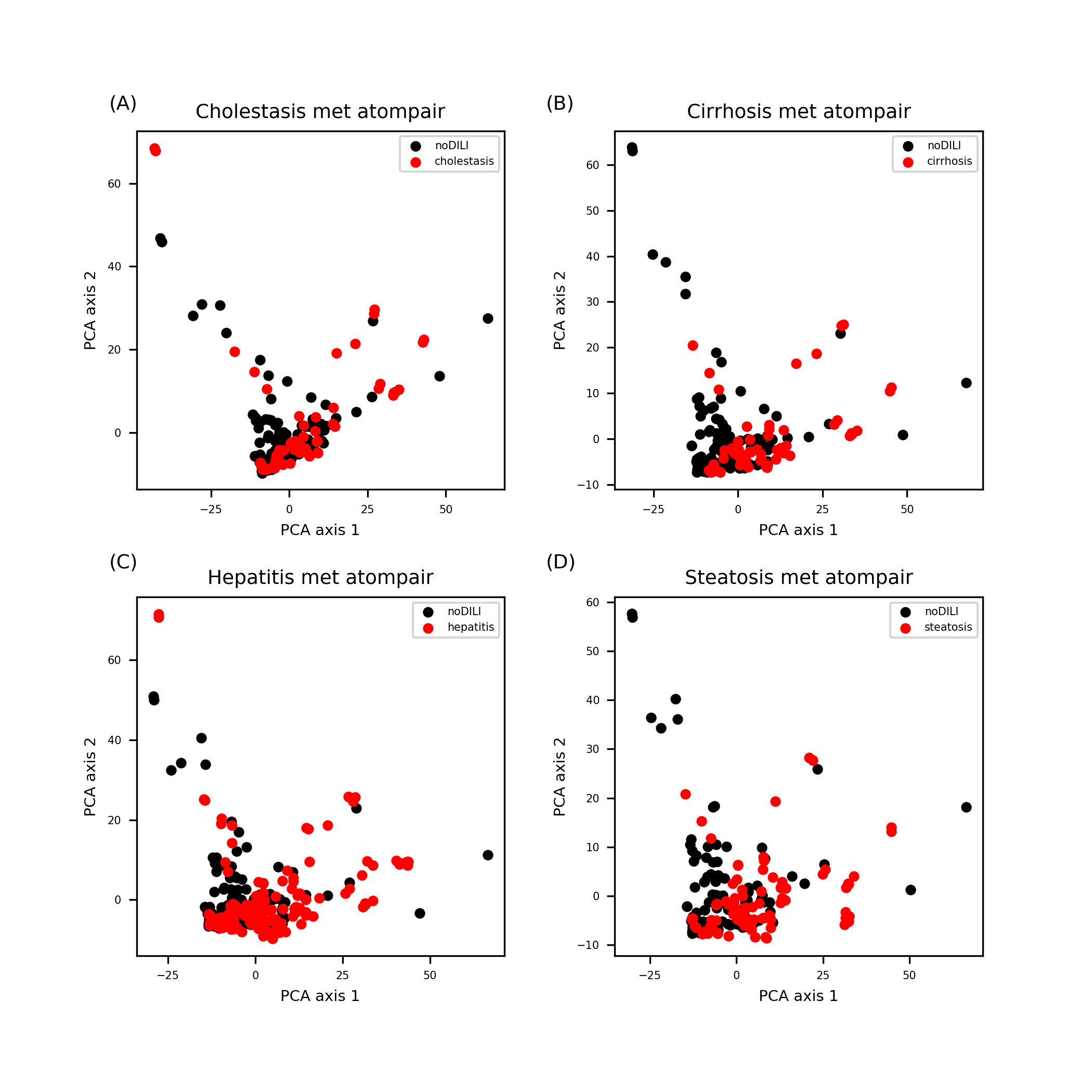


Figure S12. Atom-pair FP feature space for drug metabolite sets. Patterns are similar to distribution of atom-pair FP drug data sets (Figure S2).


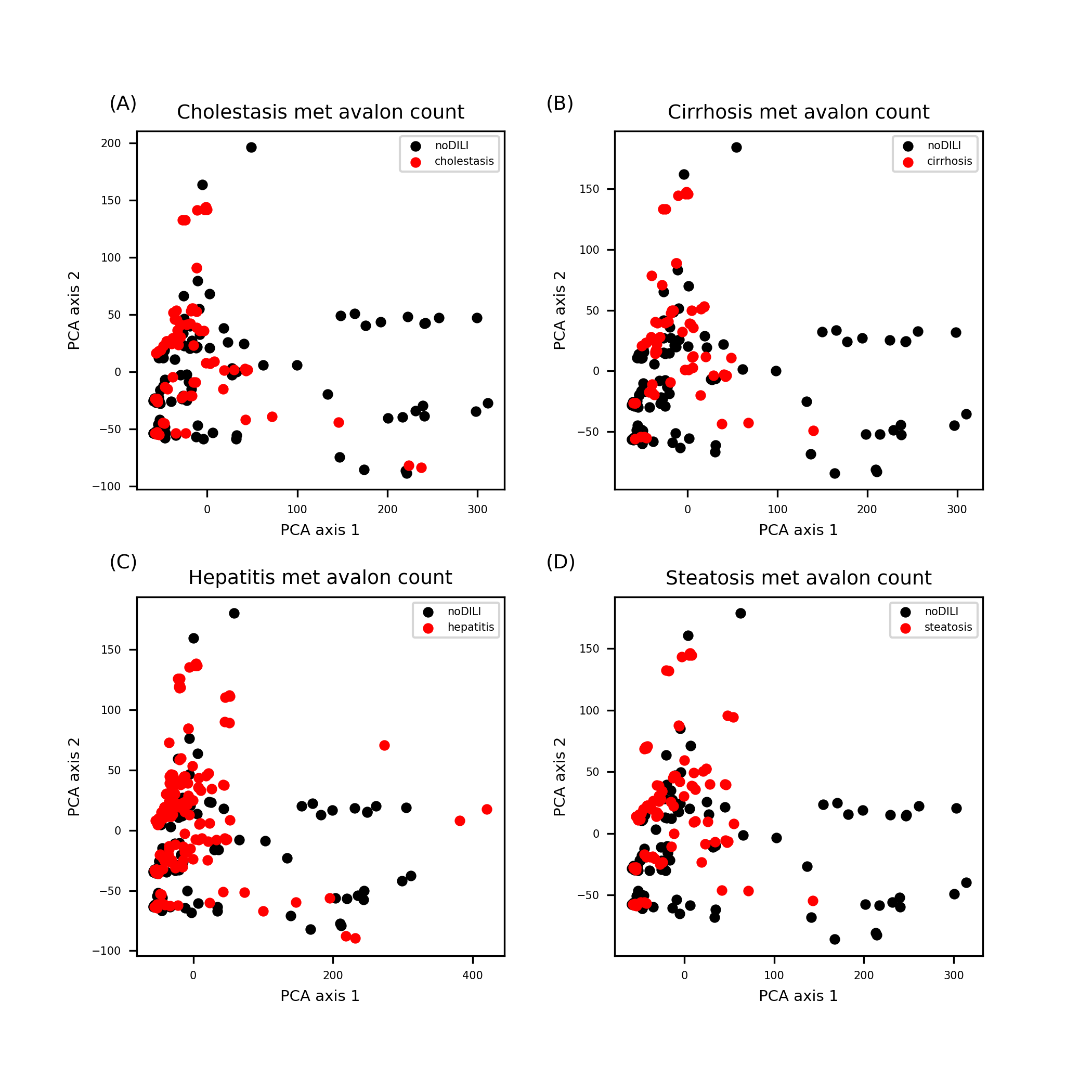


Figure S13. Avalon count FP feature space for drug metabolite sets. Patterns are similar to the distribution of Avalon count FP drug data sets (Figure S3).


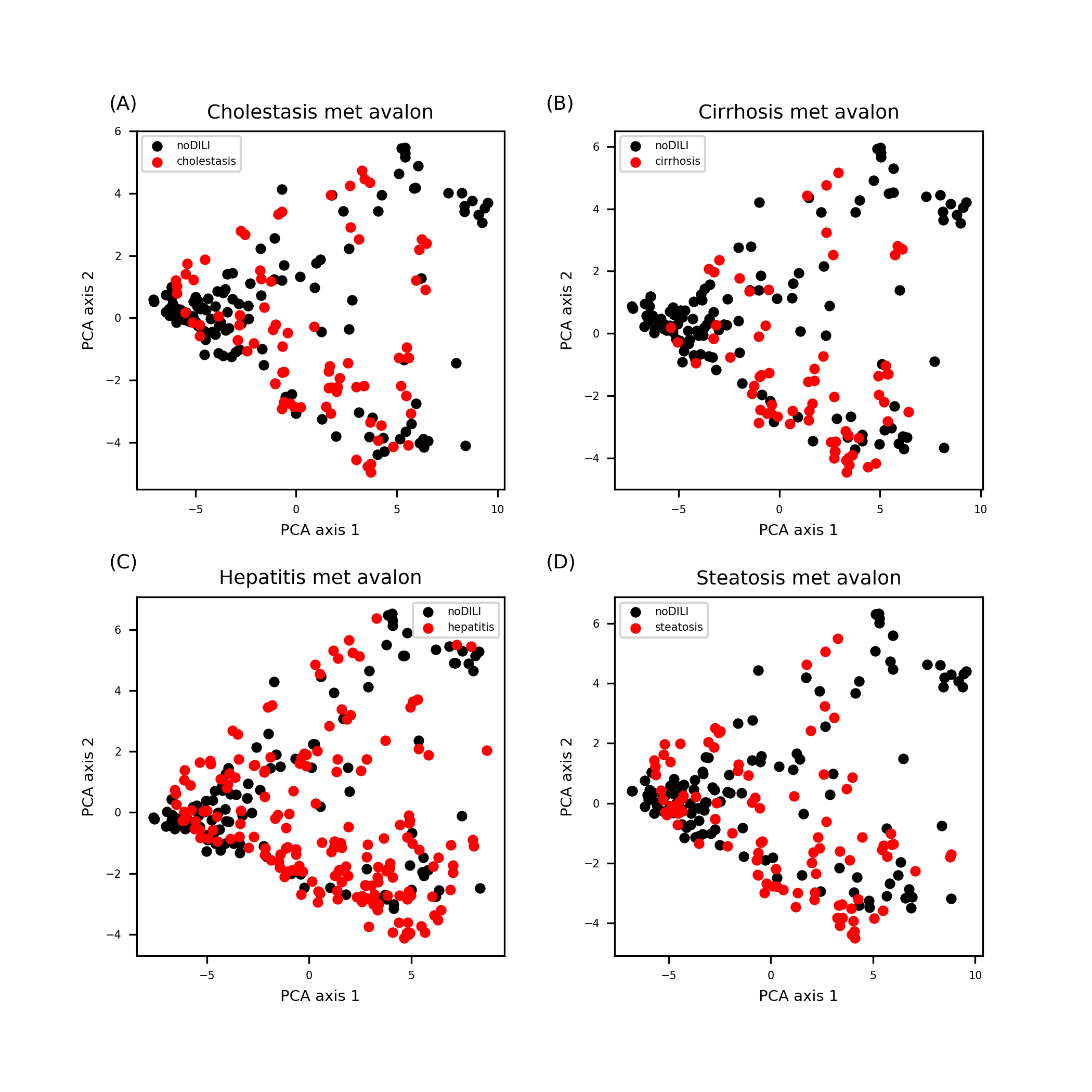


Figure S14. Avalon FP feature space for drug metabolite data sets. Patterns are similar to the distribution of Avalon FP drug data sets (Figure S4).


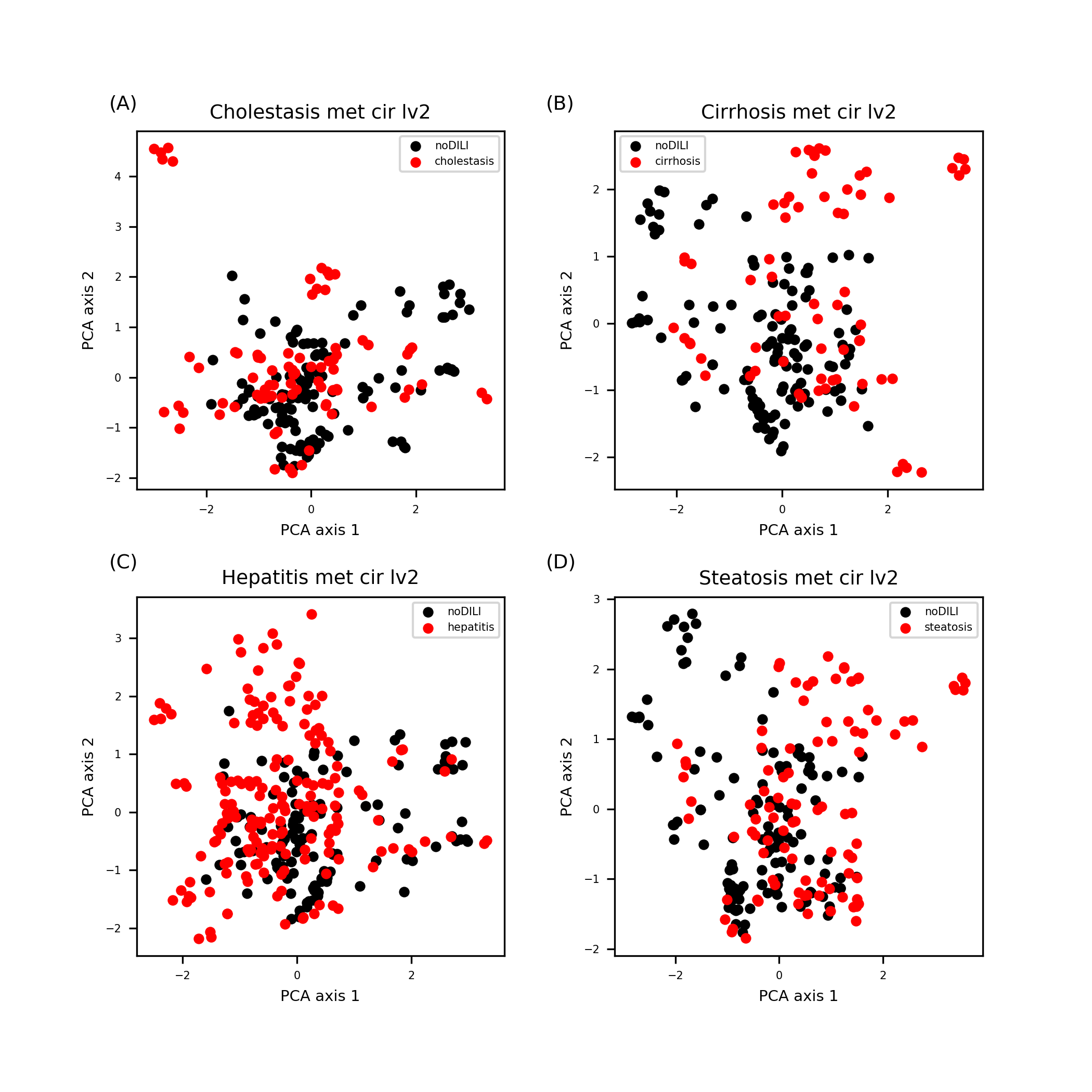


Figure S15. Morgan FP (level 2) feature space for drug metabolite data sets. Distributions in cholestasis and cirrhosis were slightly different compared to that of Morgan FP (level 2) drug data sets (Figure S5 A and B) as several DILI-positive sets were found in upside of the feature space while distributions in hepatitis and steatosis were similar to that of drug data sets (Figure S5 C and D).


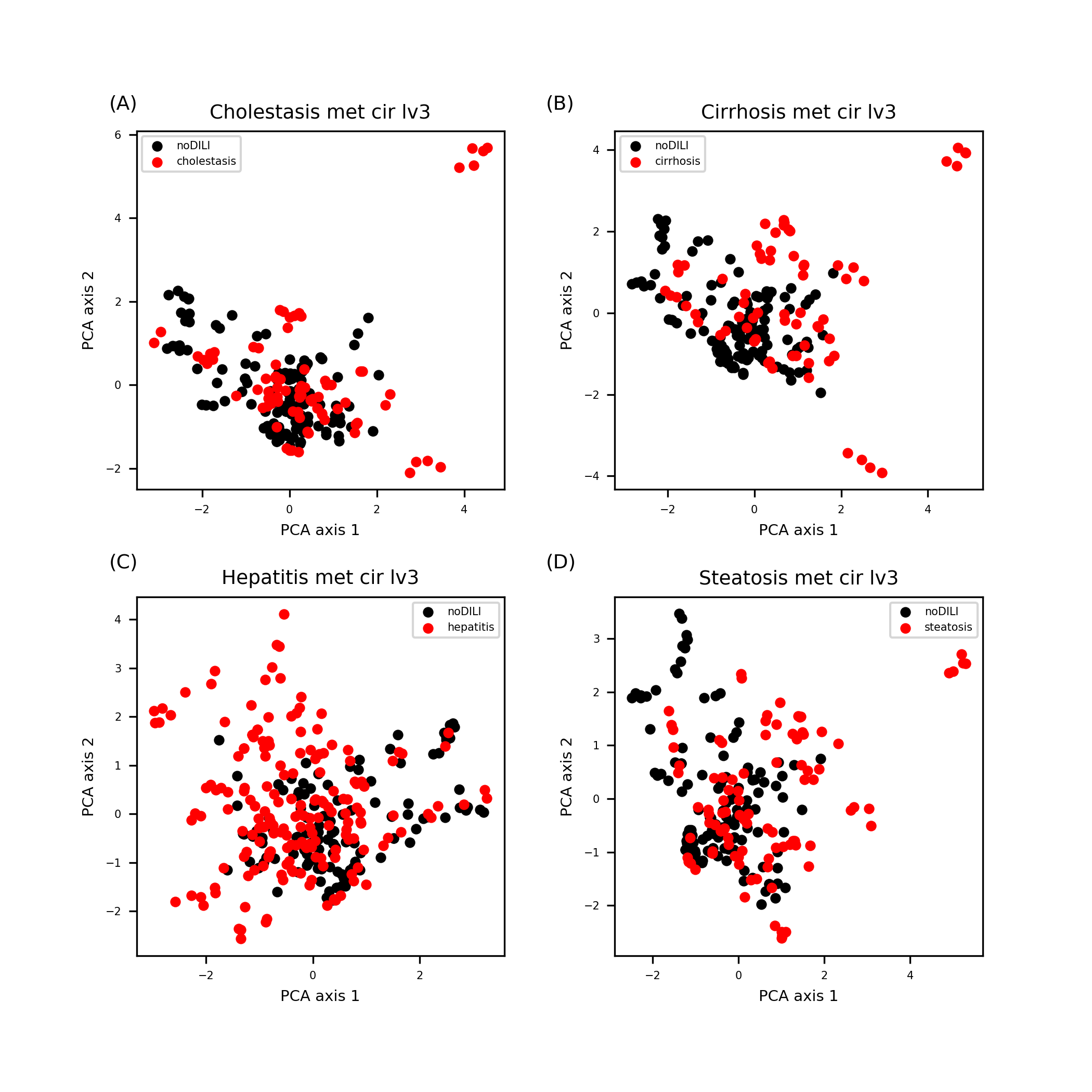


Figure S16. Morgan FP (level 3) feature space for drug metabolite data sets. Distributions in cholestasis, cirrhosis, and steatosis were slightly different compared to that of Morgan FP (level 3) drug data sets (Figure S6 A, B, and D) as several DILI-positive sets were found away from the most of data sets while distributions in hepatitis was similar to that of drug data sets (Figure S6 C).


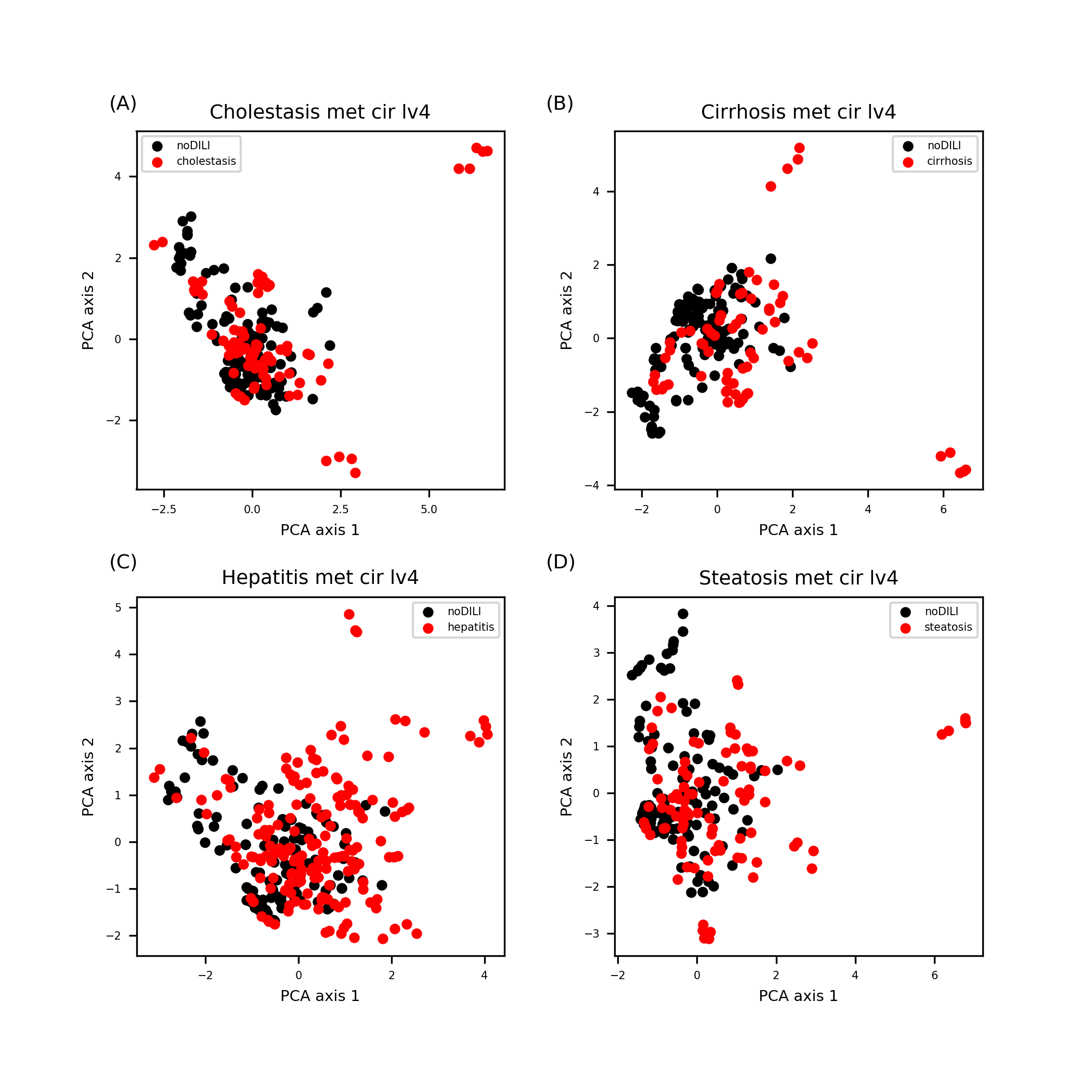


Figure S17. Morgan FP (level 4) feature space for drug metabolite data sets. Patters are similar to Figure S16 except data clusters were more clearly visible as distance from the major data points and outliers were increased.


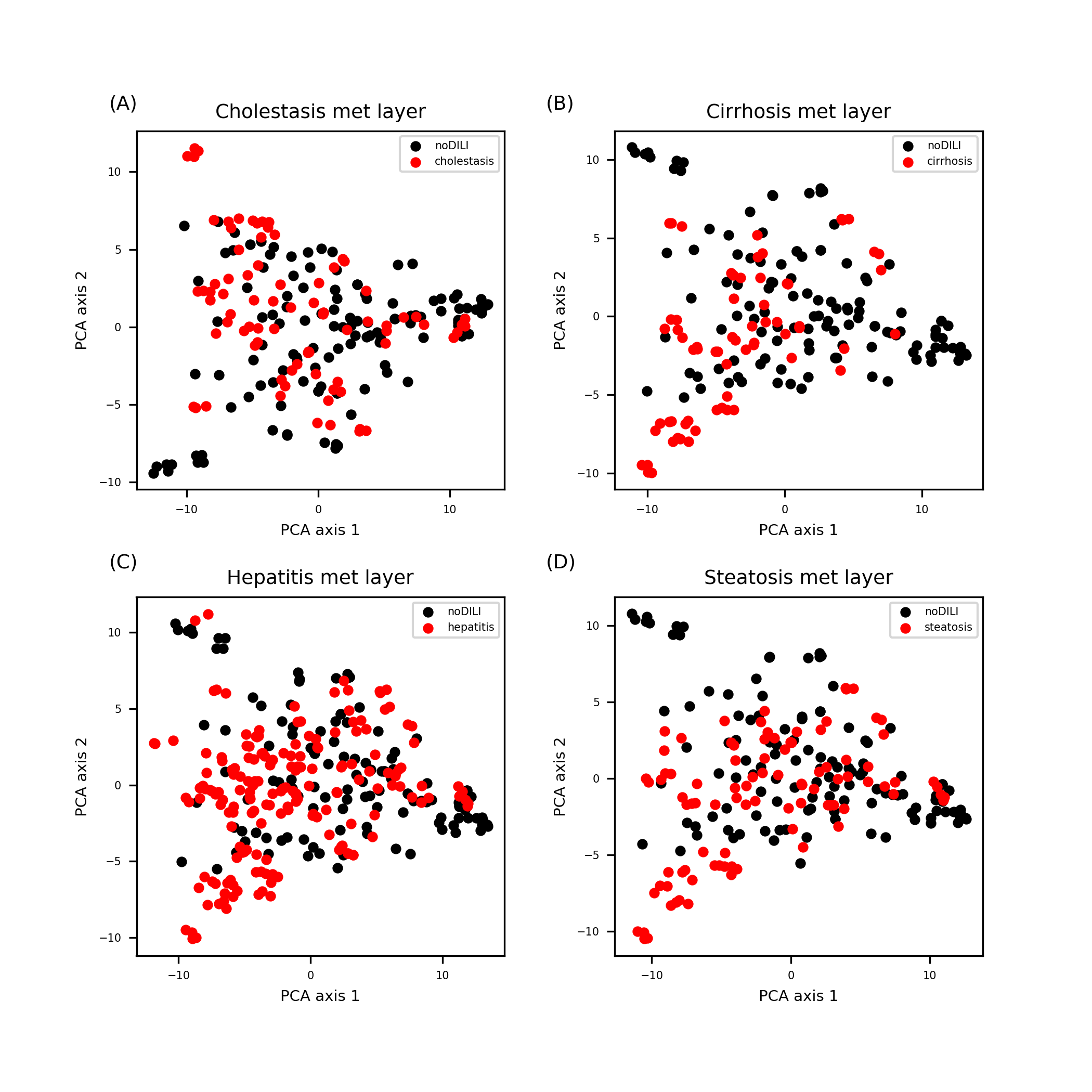


Figure S18. Layer FP feature space for drug metabolite data sets. Data points were evenly distributed throughout the feature space.


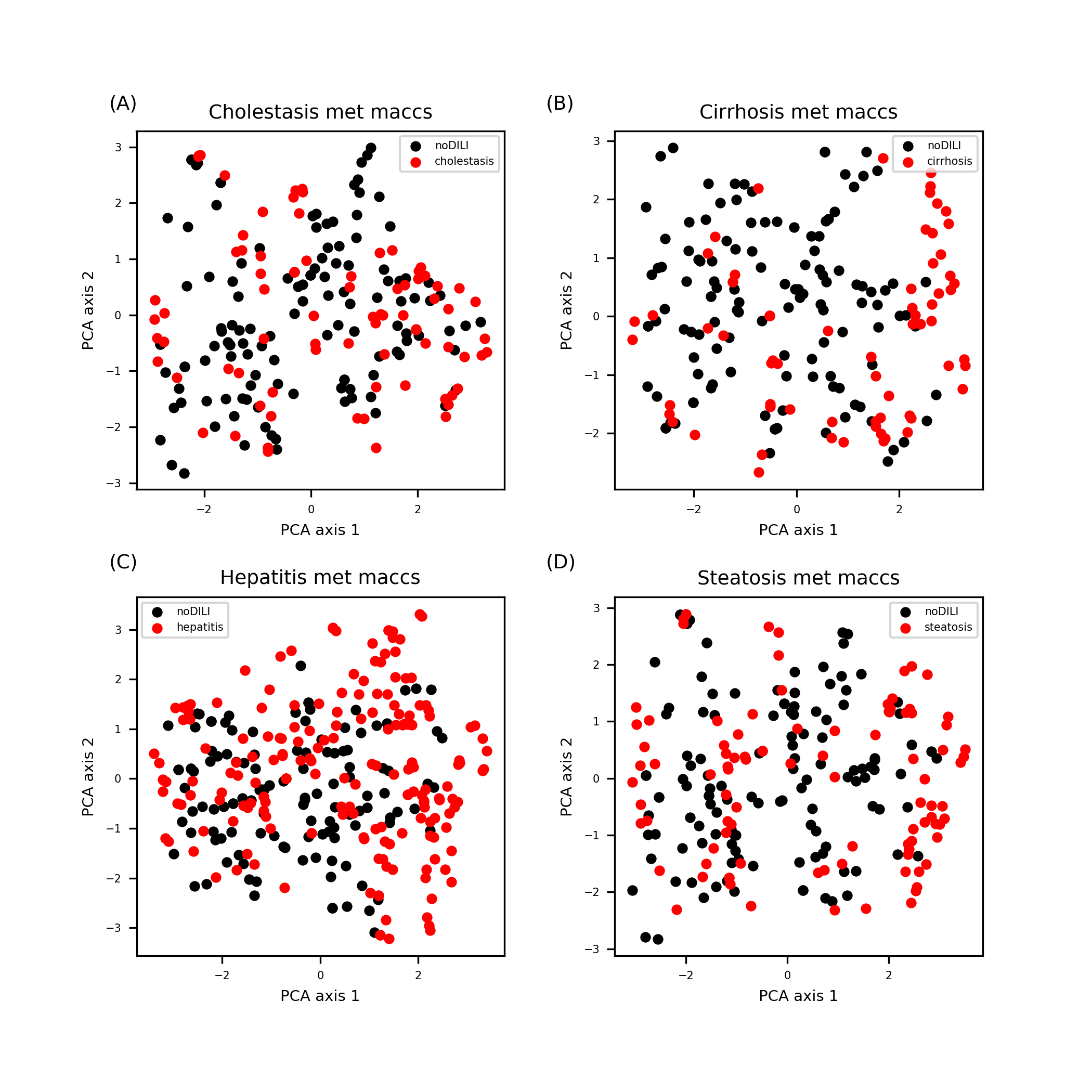


Figure S19. MACCS FP feature space for drug metabolite data sets. Data points were evenly distributed throughout the feature space.


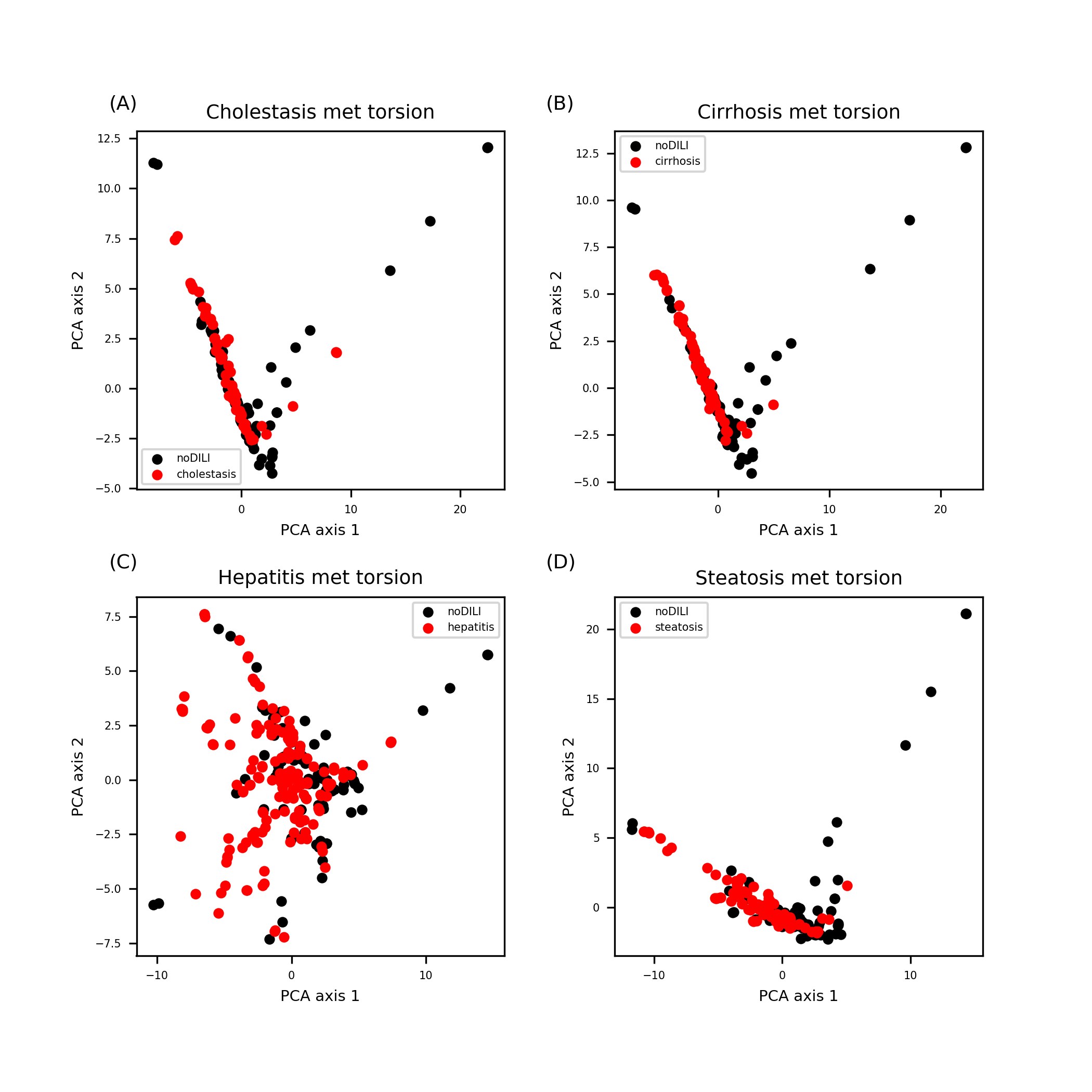


Figure S20. Topological-torsion FP feature space for drug metabolite data sets. Patterns are similar to distribution of topological-torsion FP drug data sets (Figure S10).

# Leverage analysis to define AD of drug structure-based models

Note: Blue dash-dot line is drawn for molecular weight (MW) of 100Da and 800Da. Green dotted line is the warning leverage. Any data points whose leverage is higher than the warning leverage is considered as being located outside of the model’s AD.


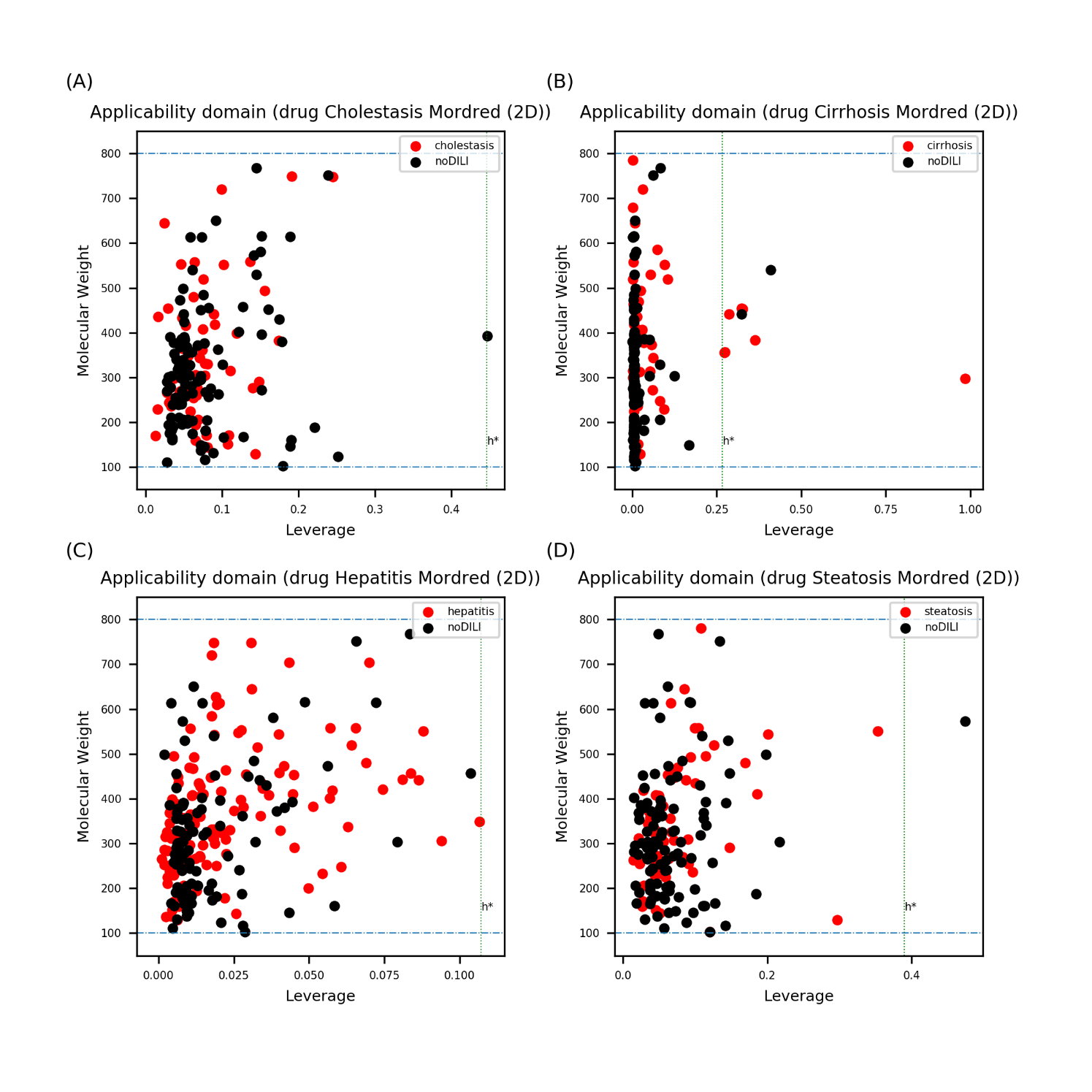


Figure S21. Leverage and MW distribution of drug data sets with 2D descriptors. Data points over the warning leverage were found in cirrhosis, and steatosis. Particularly, one DILI-positive data point had high leverage value (1.0), which is far above than the warning leverage for the cirrhosis 2D data set.


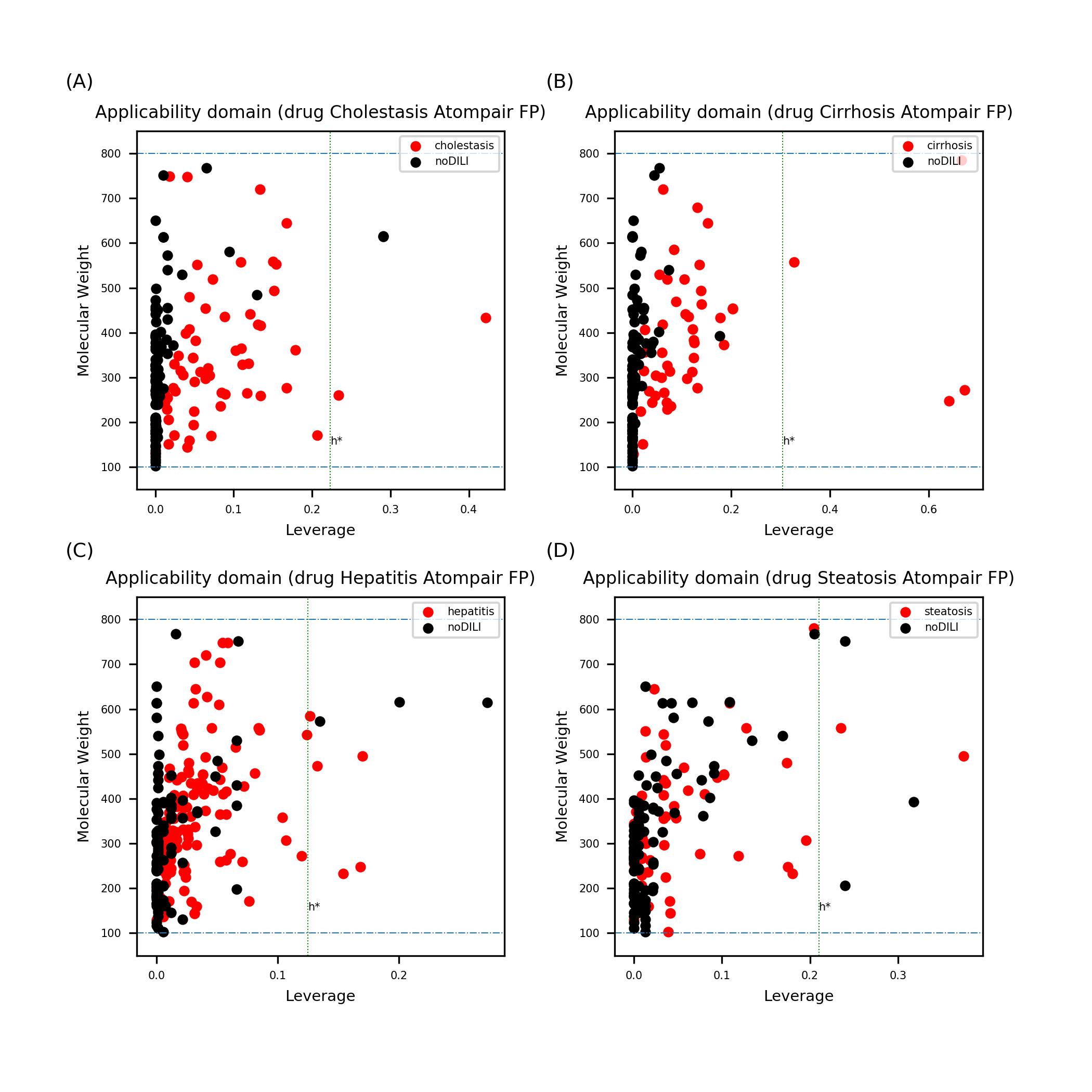


Figure S22. Leverage and MW of drug data sets with Atom-pair FP. Data points exceeding the warning leverage were found for all data sets. Highest leverages were found in the cirrhosis atom-pair FP data set.


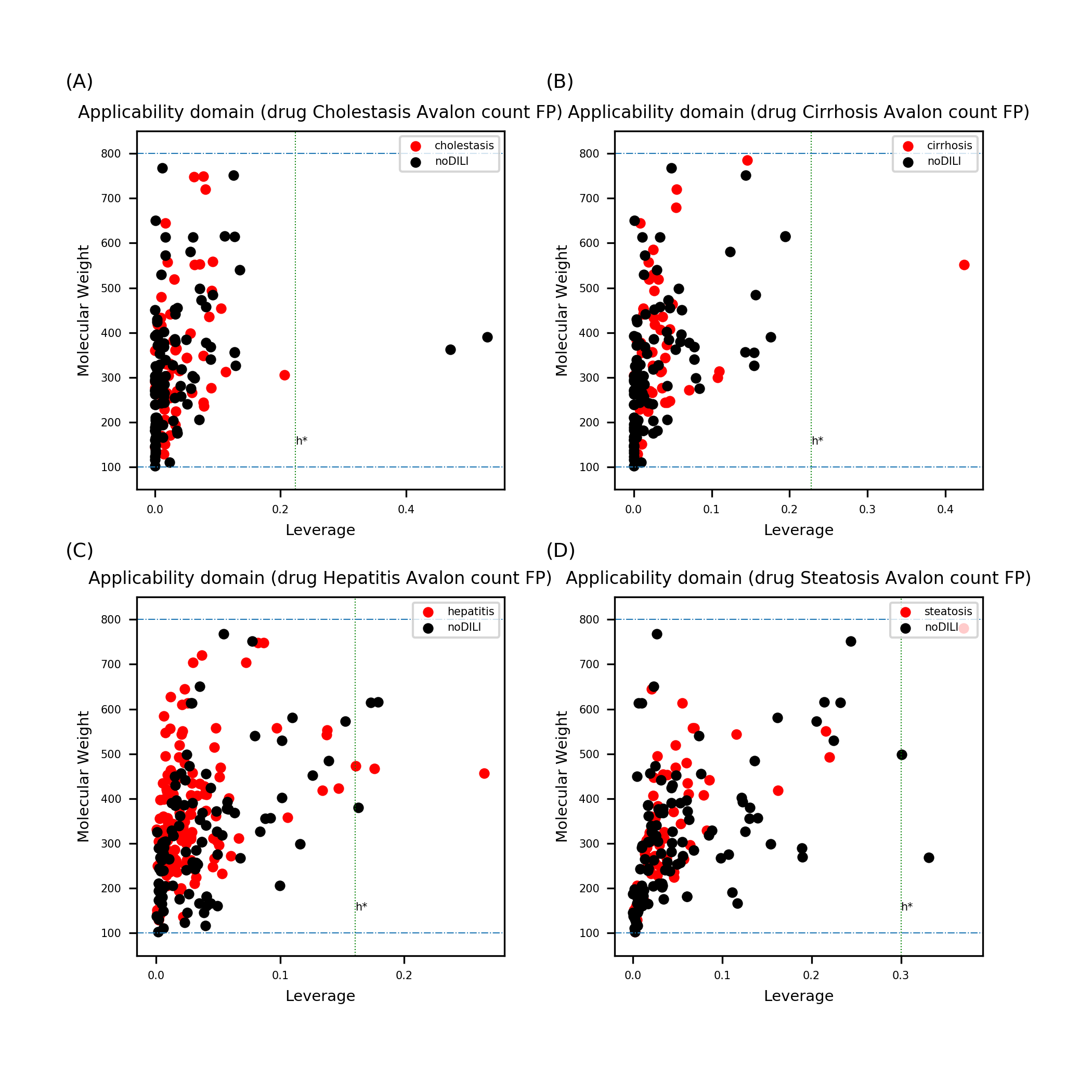


Figure S23. Leverage and MW of drug data sets with Avalon count FP. Data points exceeding the warning leverage were found for all data sets.


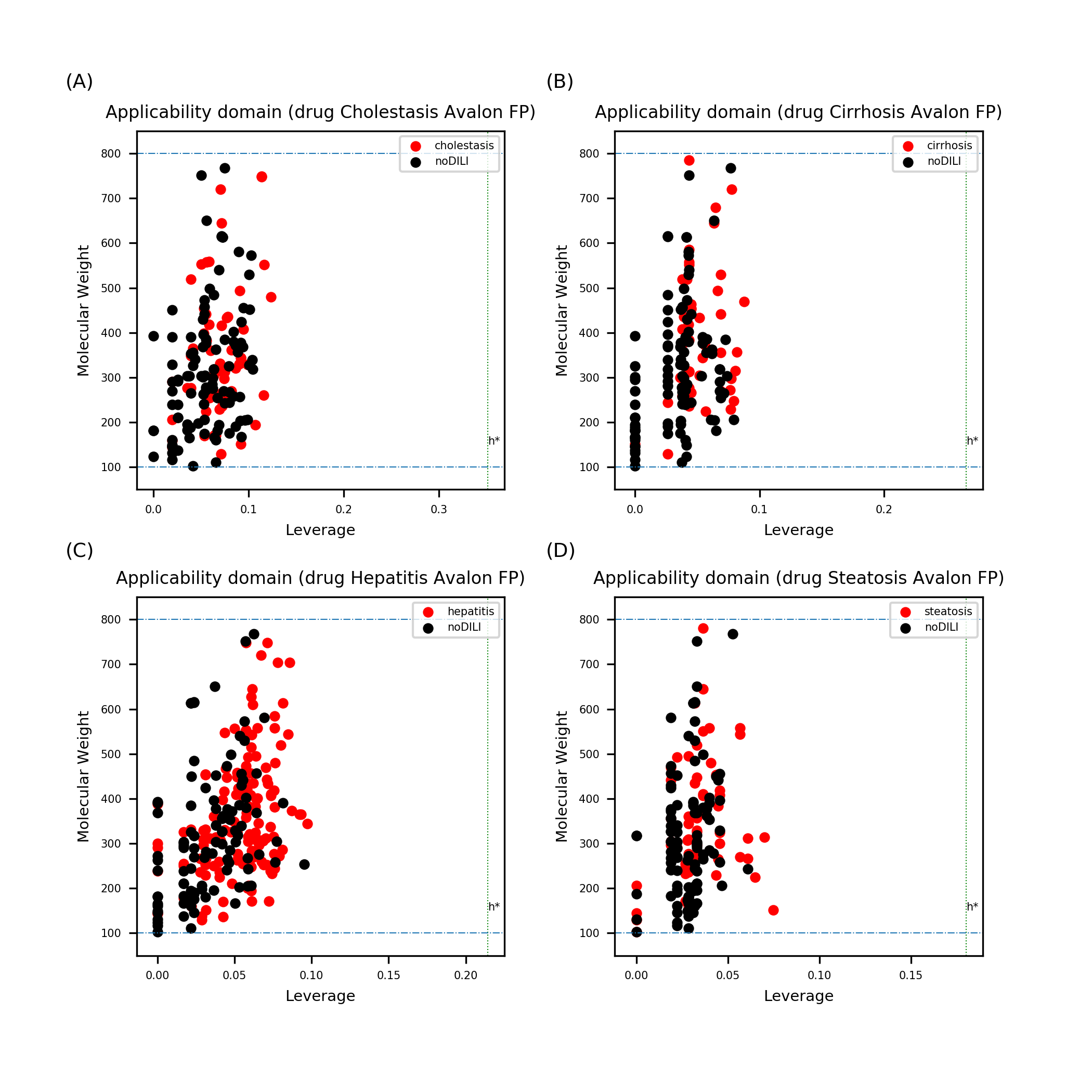


Figure S24. Leverage and MW of drug data sets with Avalon FP. No data points were found to exceed the warning leverage.


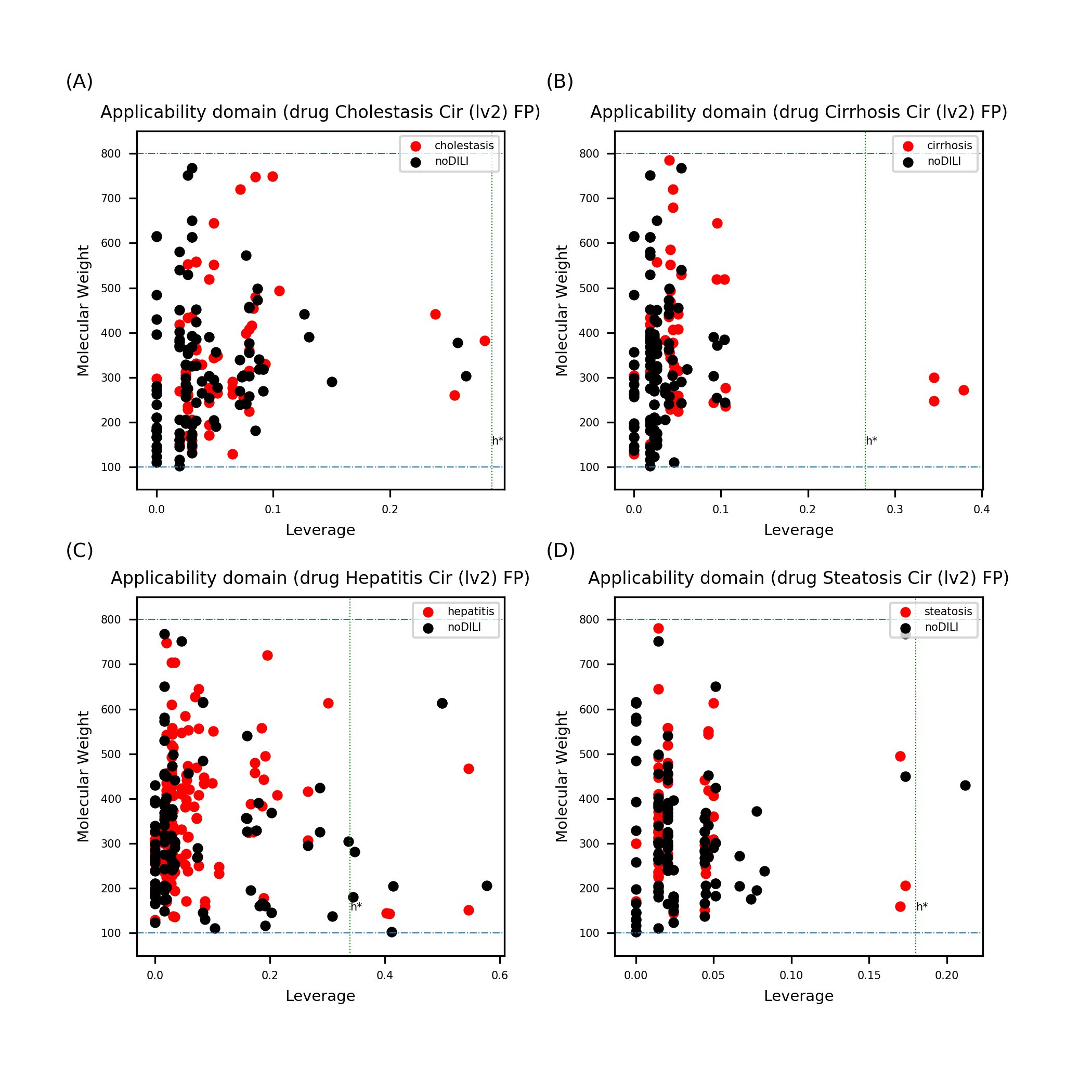


Figure S25. Leverage and MW of drug data sets with Morgan FP (level 2). Data points exceeding the warning leverage were found for all data sets except cholestasis.


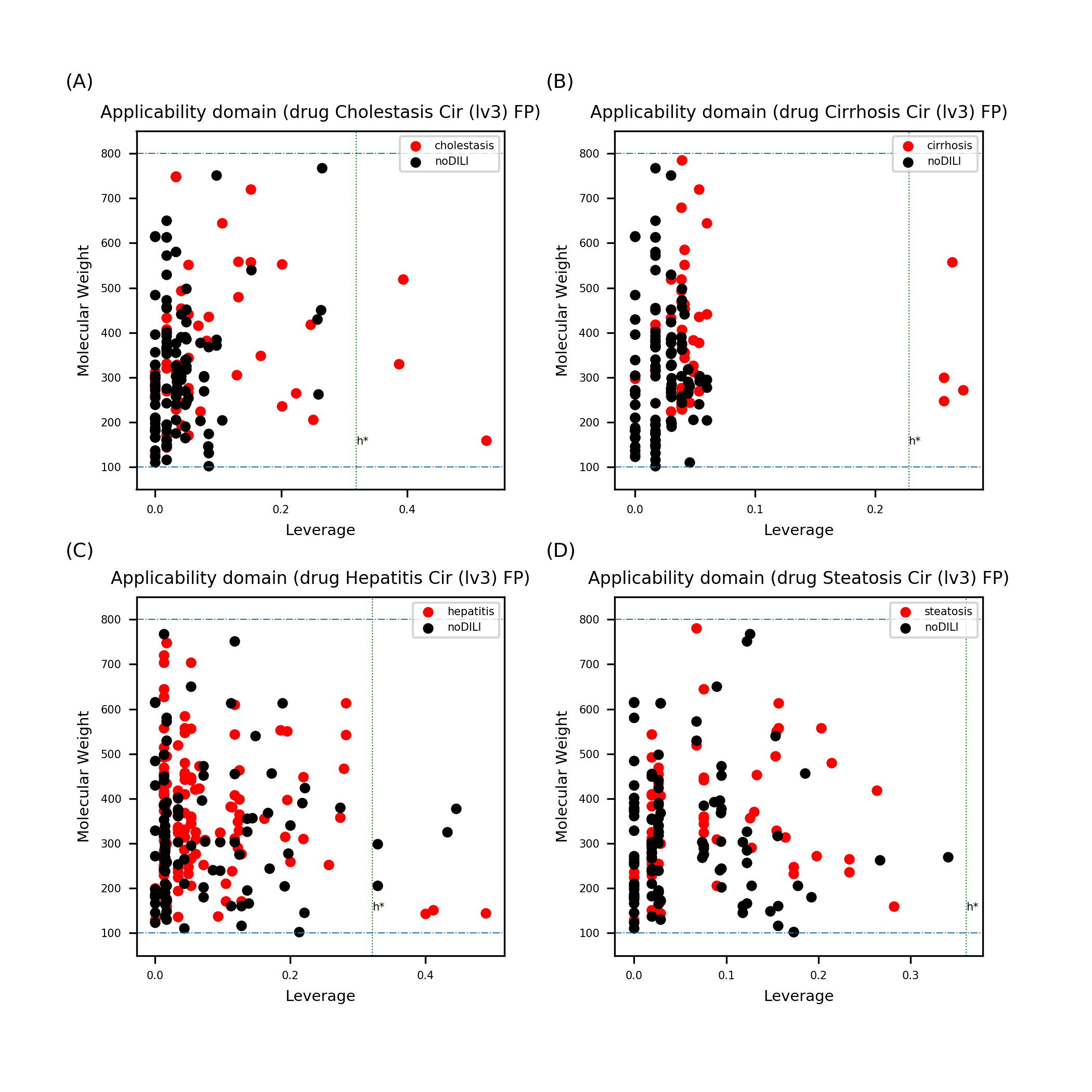


Figure S26. Leverage and MW of drug data sets with Morgan FP (level 3). Data points exceeding the warning leverage were found for all data sets except steatosis. Compared to result in Figure S25 (Morgan FP level 2), more data points were over the warning leverage for cholestasis and cirrhosis while less data points exceeded the warning leverage for hepatitis and steatosis.


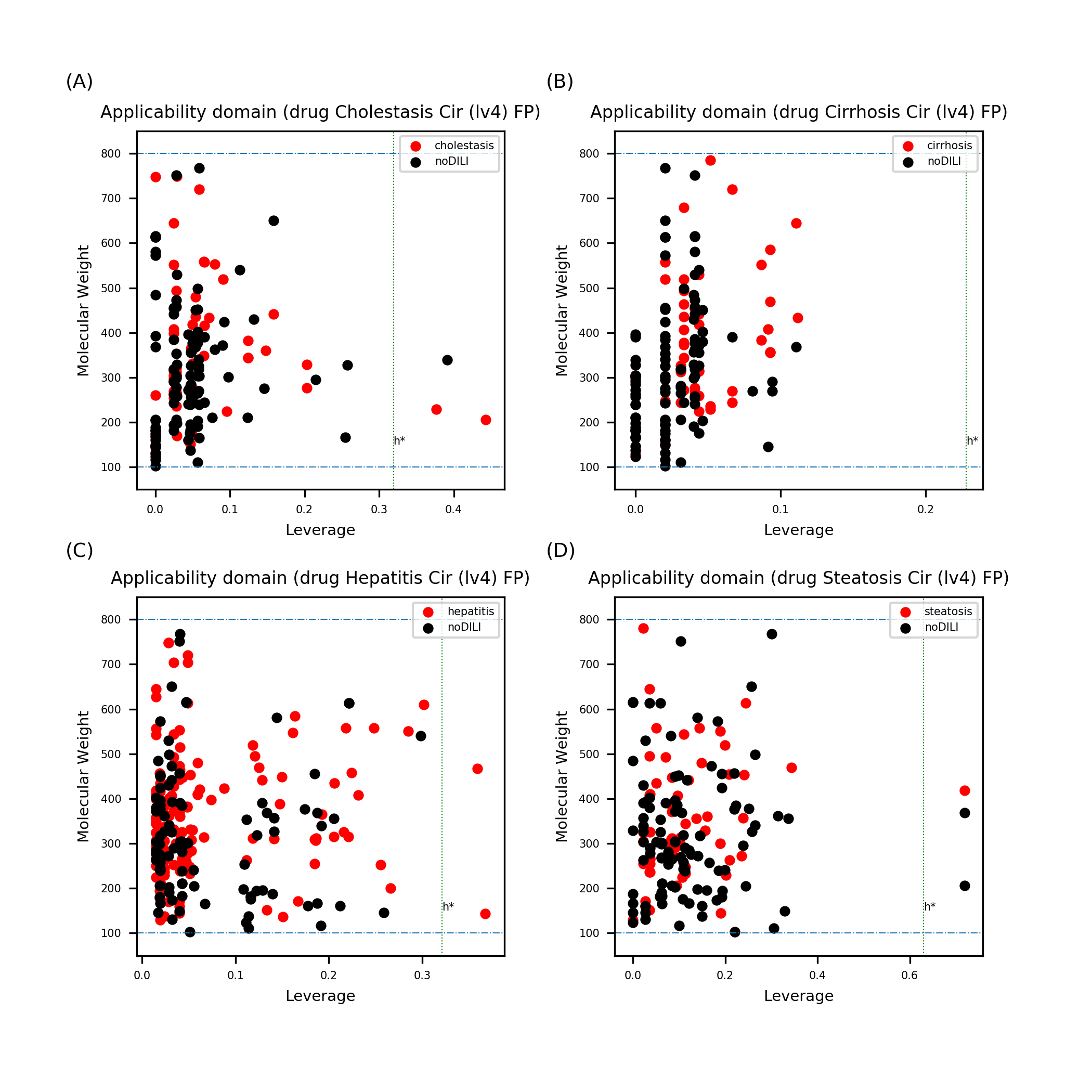


Figure S27. Leverage and MW of drug data sets with Morgan FP (level 4). Data points exceeding the warning leverage were found for all data sets except cirrhosis.


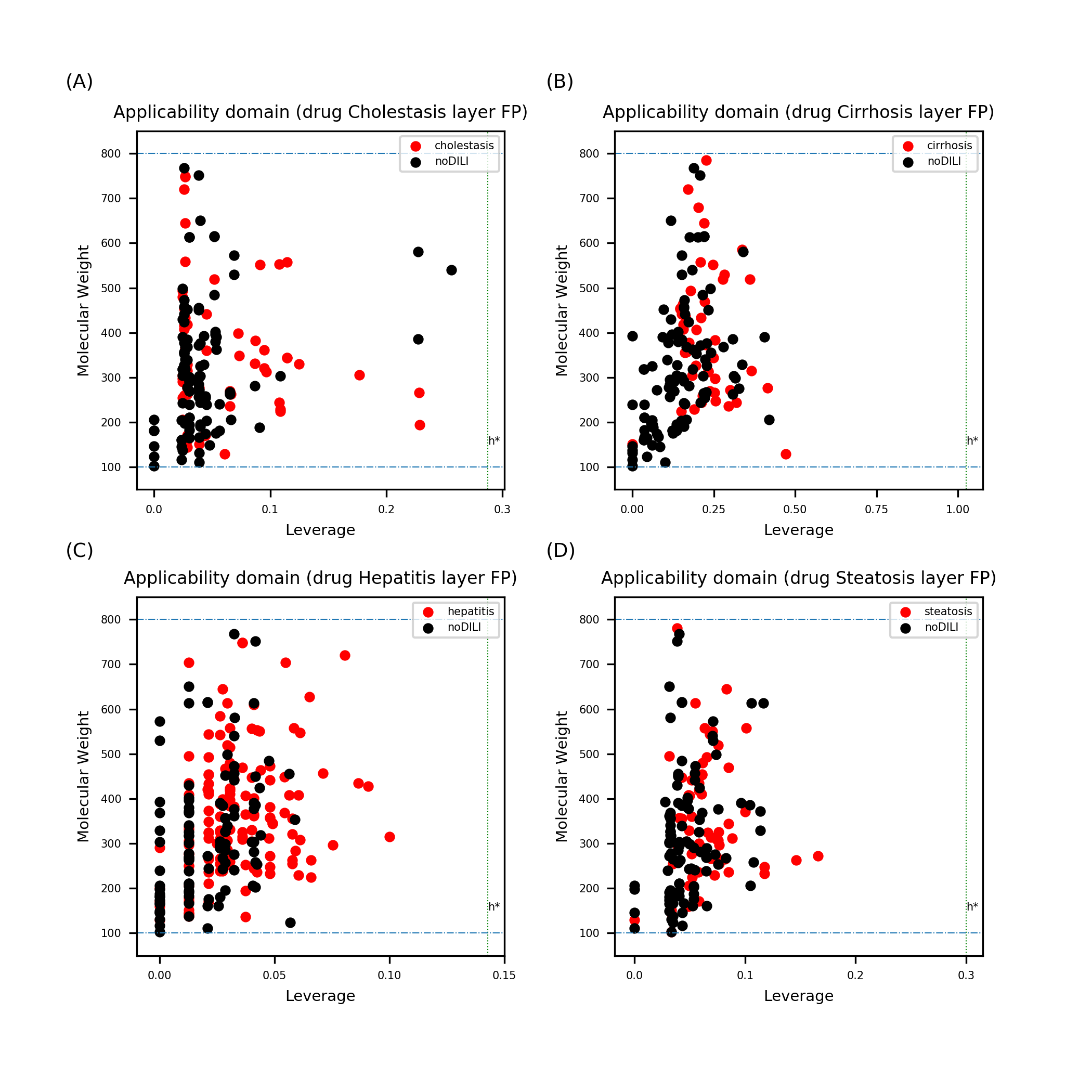


Figure S28. Leverage and MW of drug data sets with layer FP. No data points were found to exceed the warning leverage.


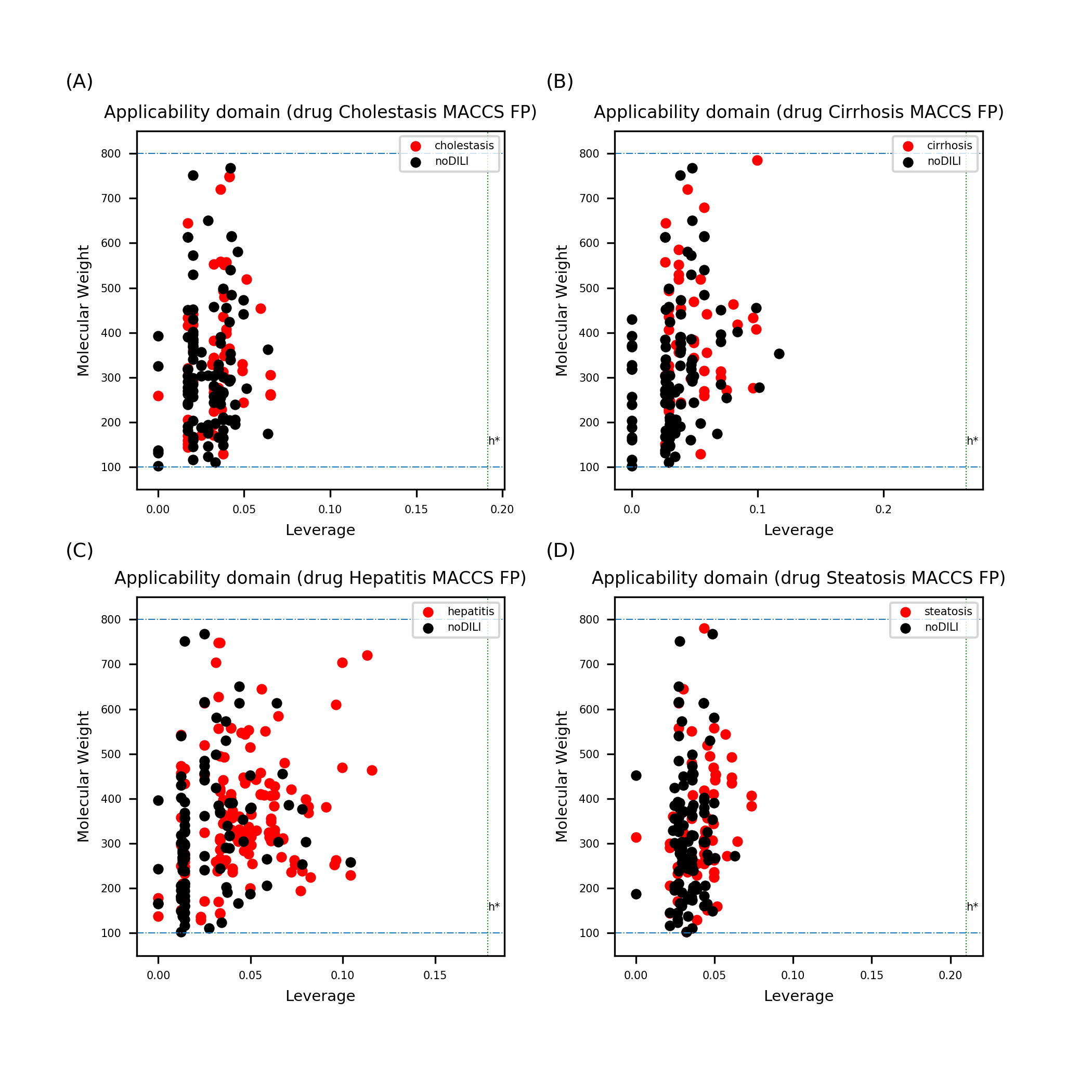


Figure S29. Leverage and MW of drug data sets with MACCS FP. No data points were found to exceed the warning leverage.


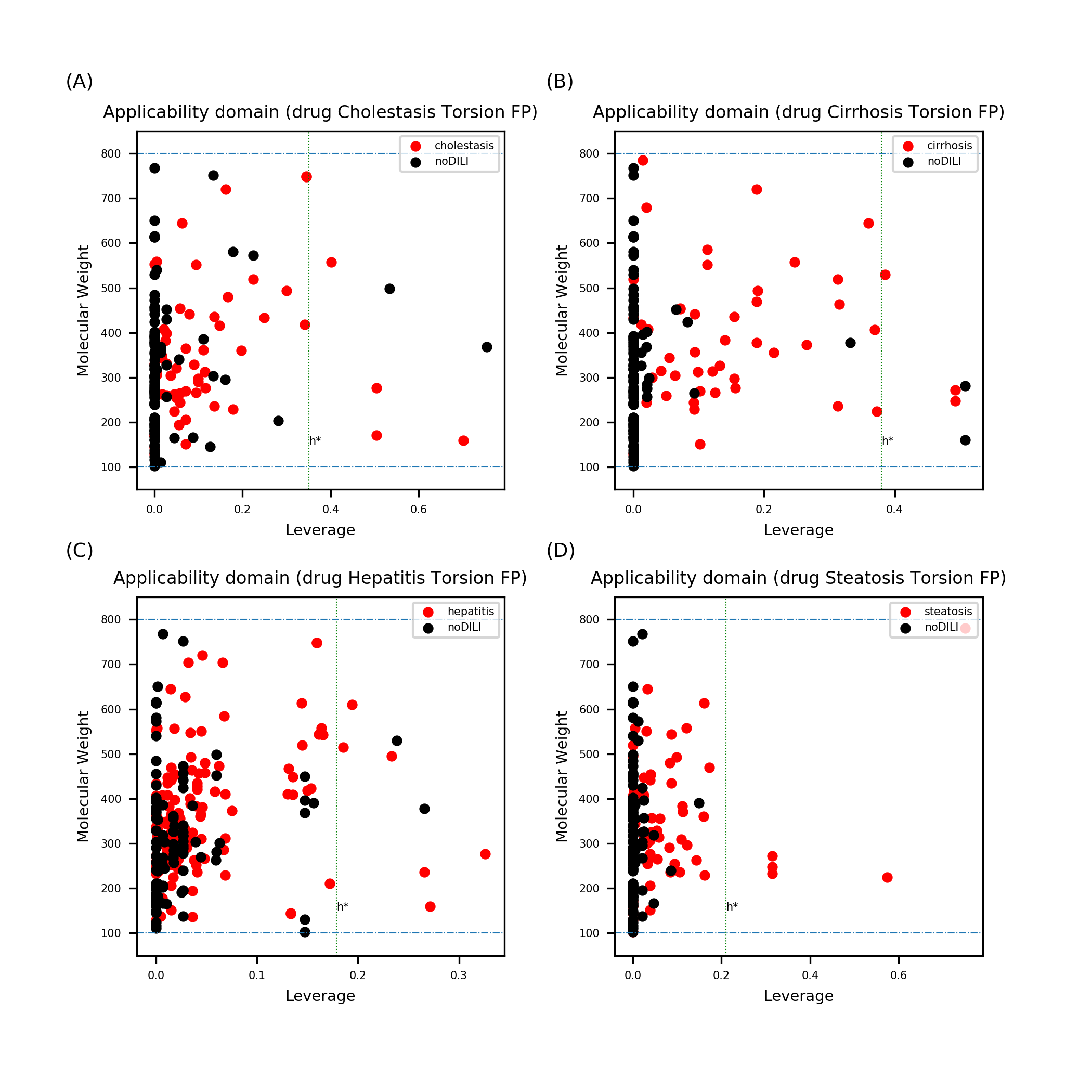


Figure S30. Leverage and MW of drug data sets with topological-torsion FP. Several data points were found to exceed the warning leverage in all data sets.

# Leverage analysis to define AD of drug metabolite structure-based models

*Note: In metabolite data sets, few DILI-negative drug metabolites were found to be less than 100 Da. In some cases of dealkylation, drug molecules were broken into two fragments. As small number of drug metabolites were found for DILI-negative drugs, both metabolism products were included in the data set. Therefore, drug metabolites smaller than 100Da were derived from small fragment produced from dealkylation of phase I drug metabolism.


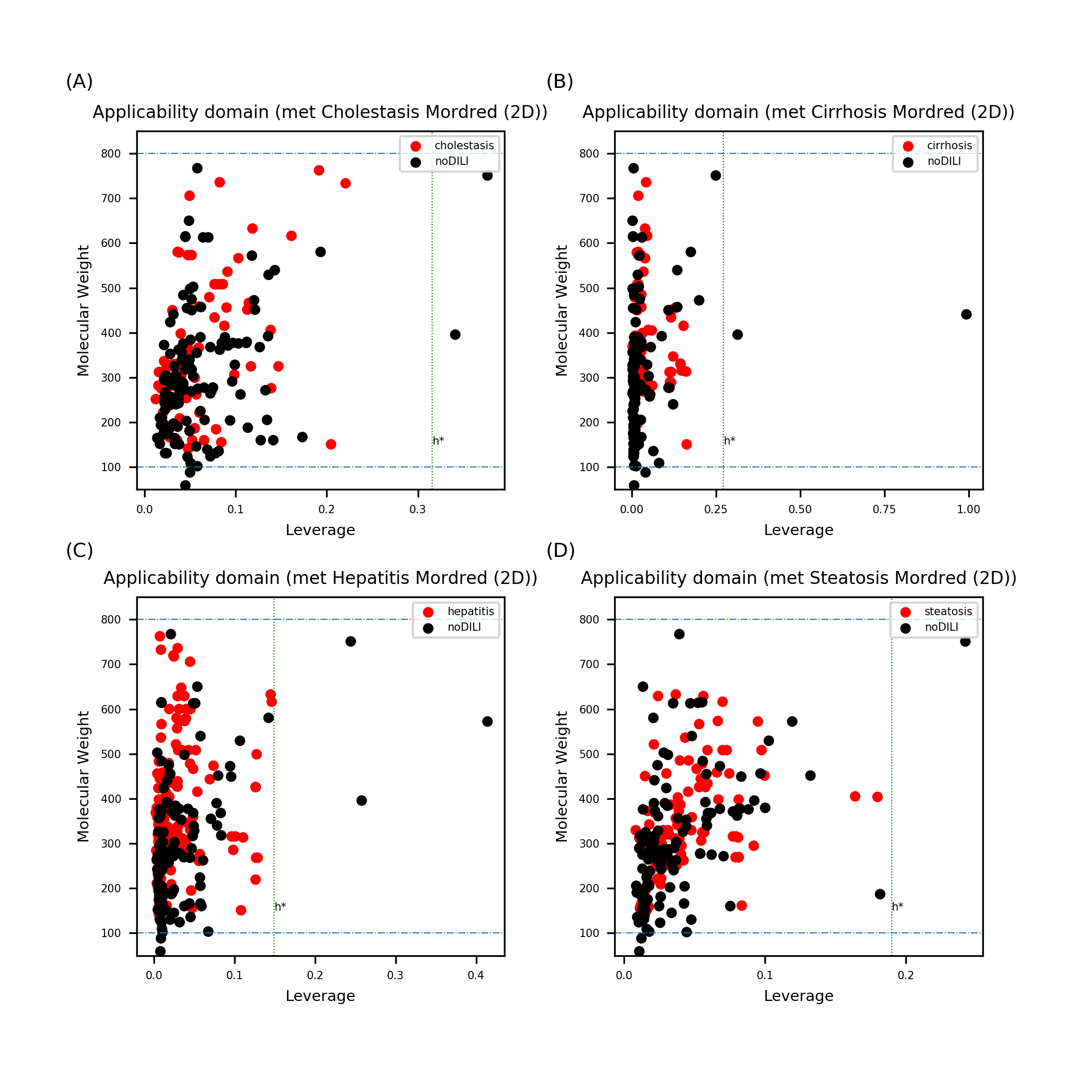


Figure S31. Leverage and MW of drug metabolite data sets with 2D descriptors. Data points exceeding the warning leverage were found in all data sets.


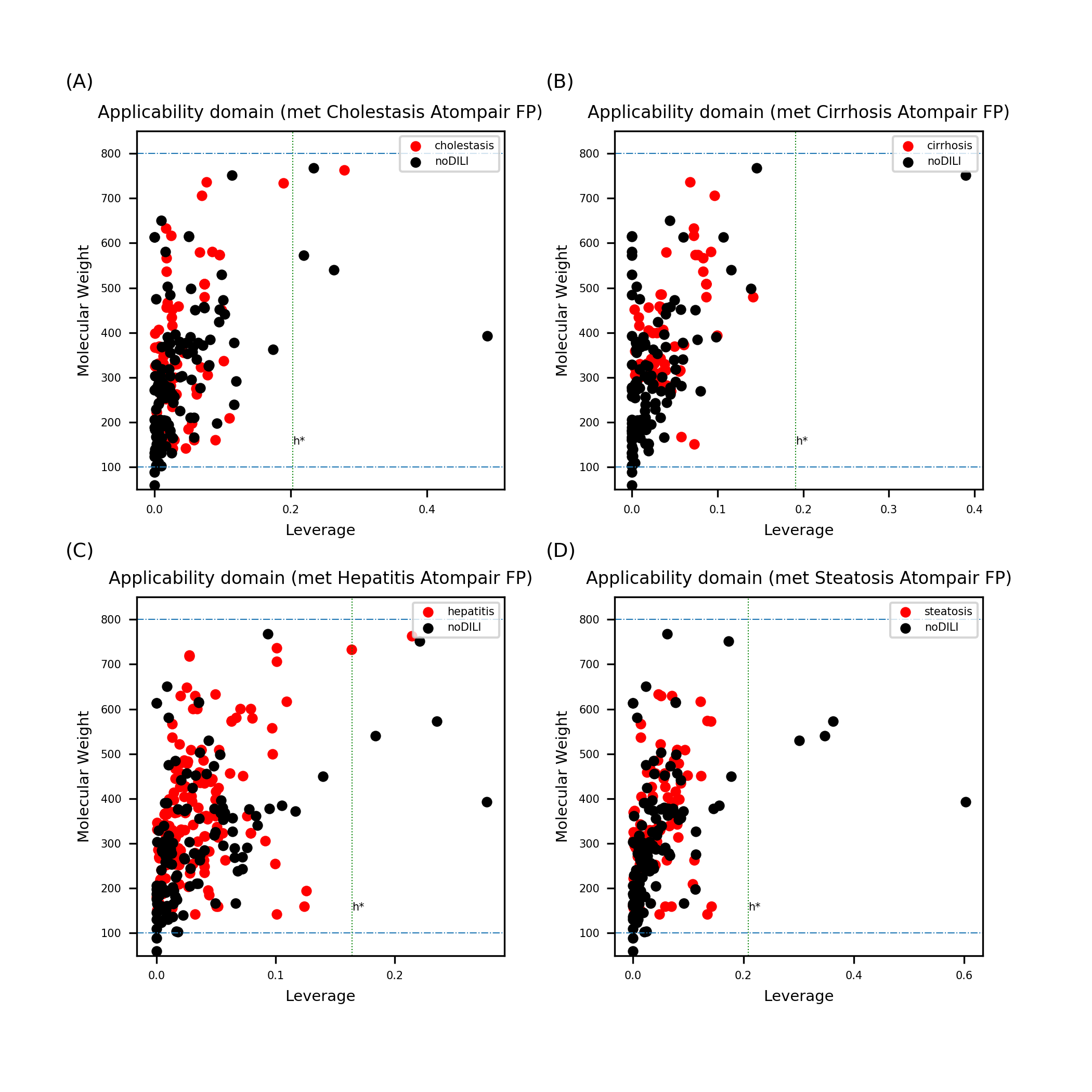


Figure S32. Leverage and MW of drug metabolite data sets with atom-pair FP. Data points exceeding the warning leverage were found in all data sets.


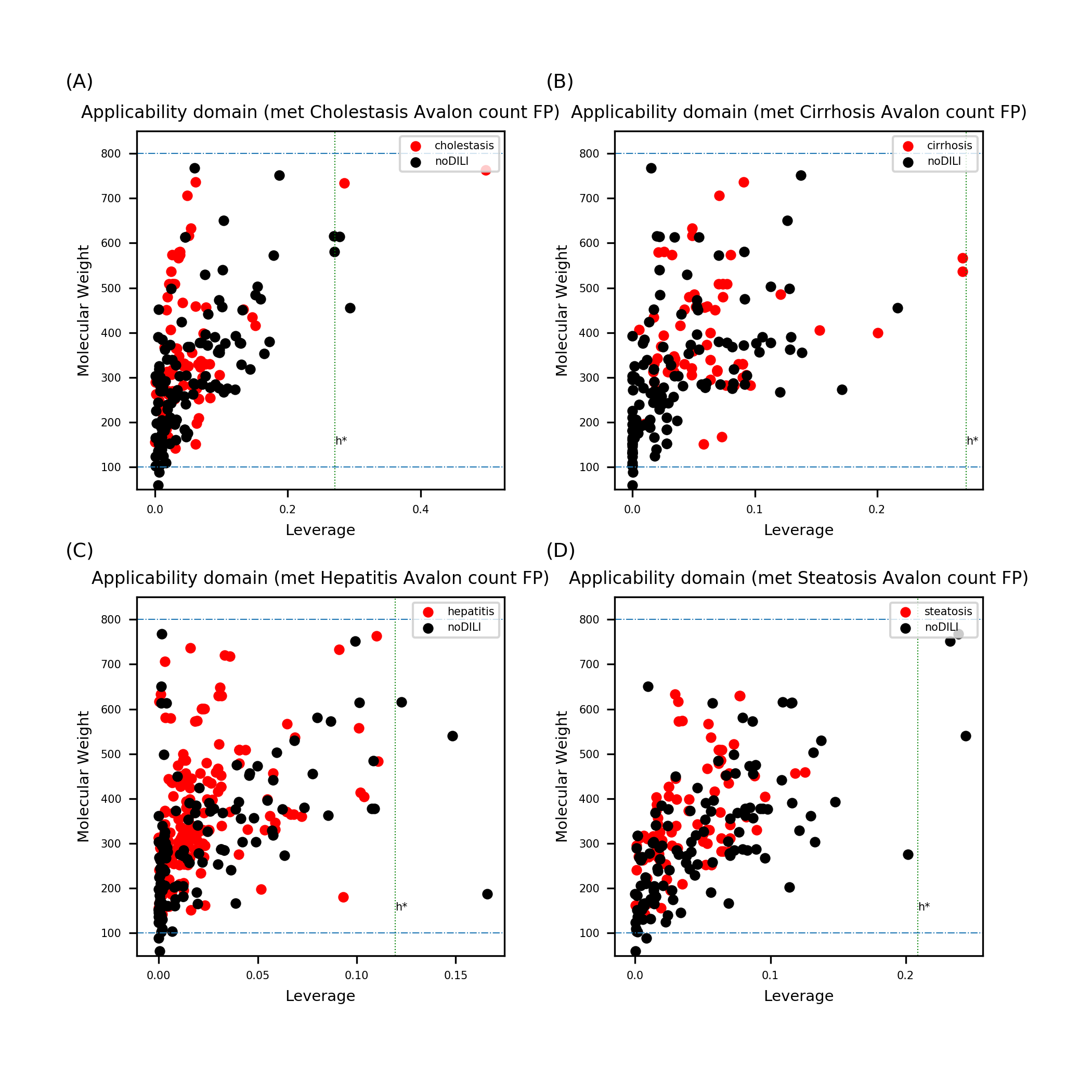


Figure S33. Leverage and MW of drug metabolite data sets with Avalon count FP. Data points exceeding the warning leverage were found in all data sets except cirrhosis.


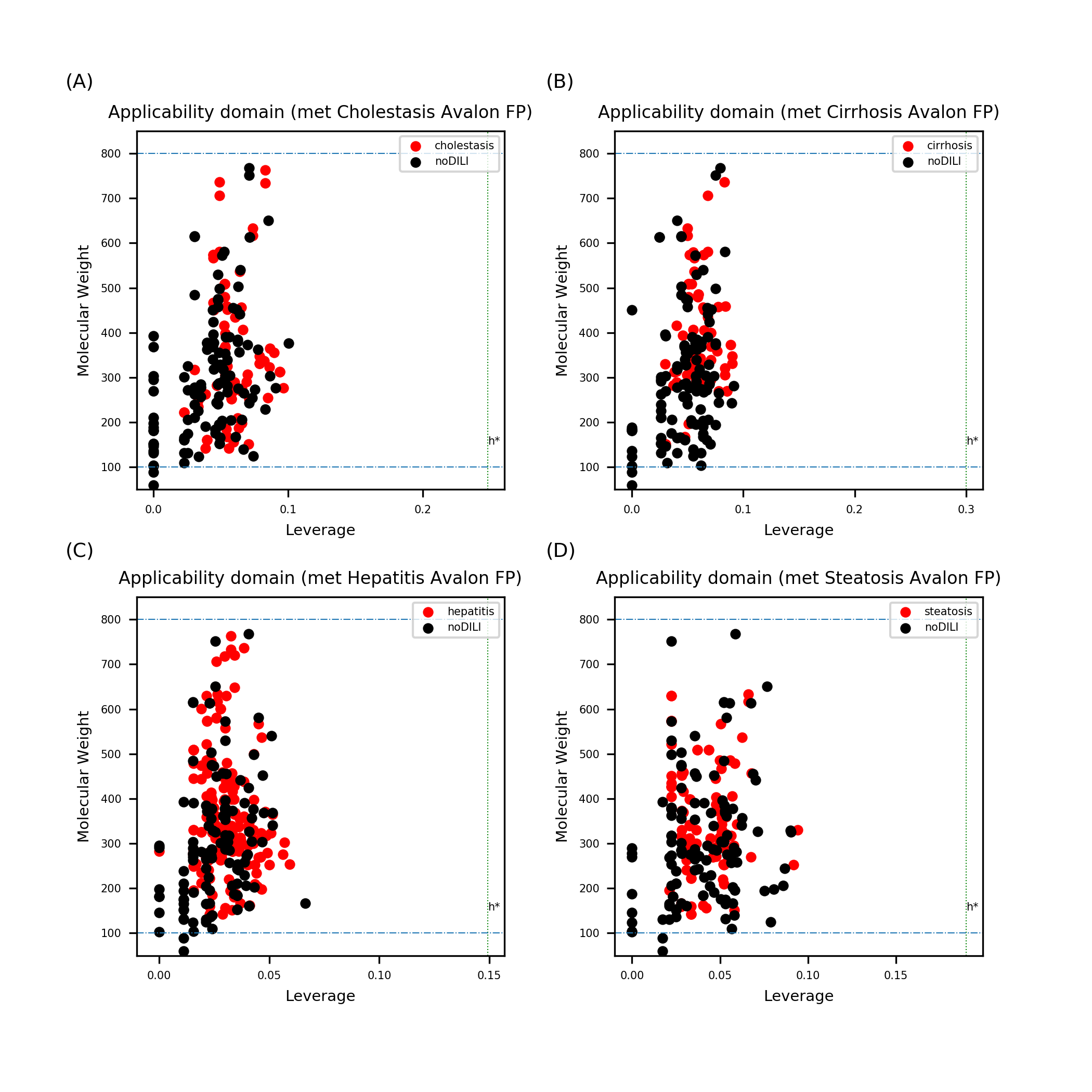


Figure S34. Leverage and MW of drug metabolite data sets with Avalon FP. No data points exceeded the warning leverage.


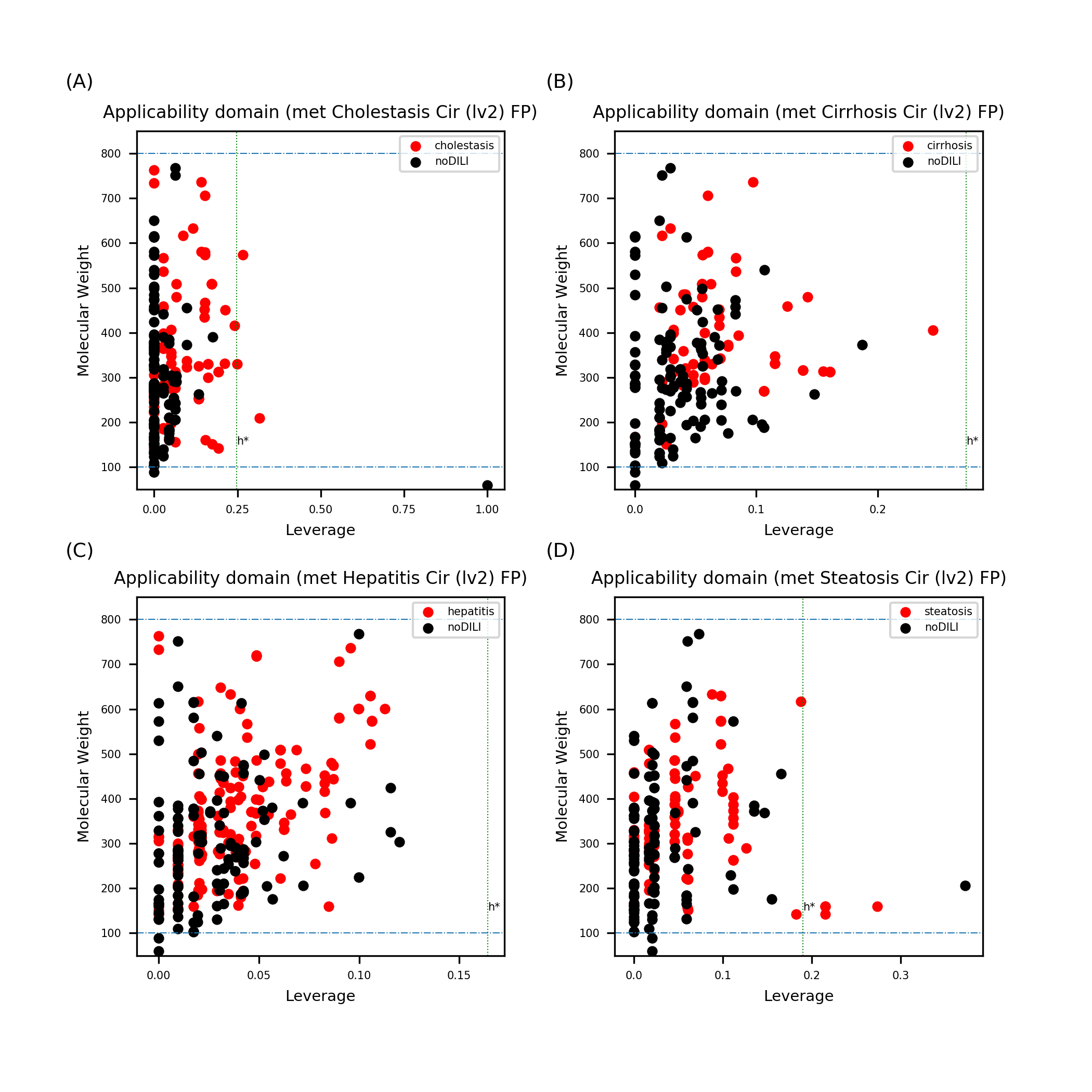


Figure S35. Leverage and MW of drug metabolite sets with Morgan FP (level 2). Data points exceeding the warning leverage were found in cholestasis and steatosis.


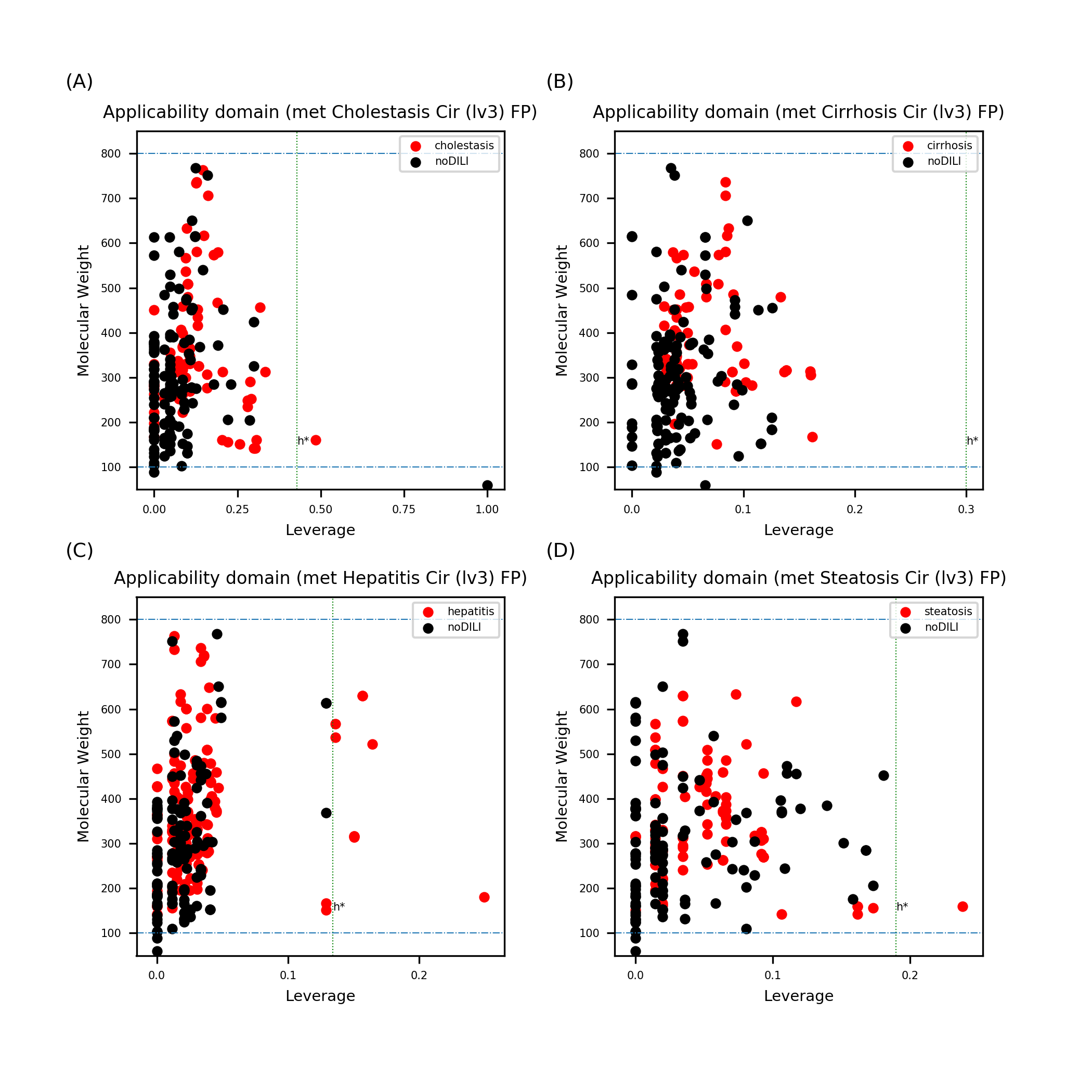


Figure S36. Leverage and MW of drug metabolite sets with Morgan FP (level 3). Data points exceeding the warning leverage were found in all data sets except cirrhosis.


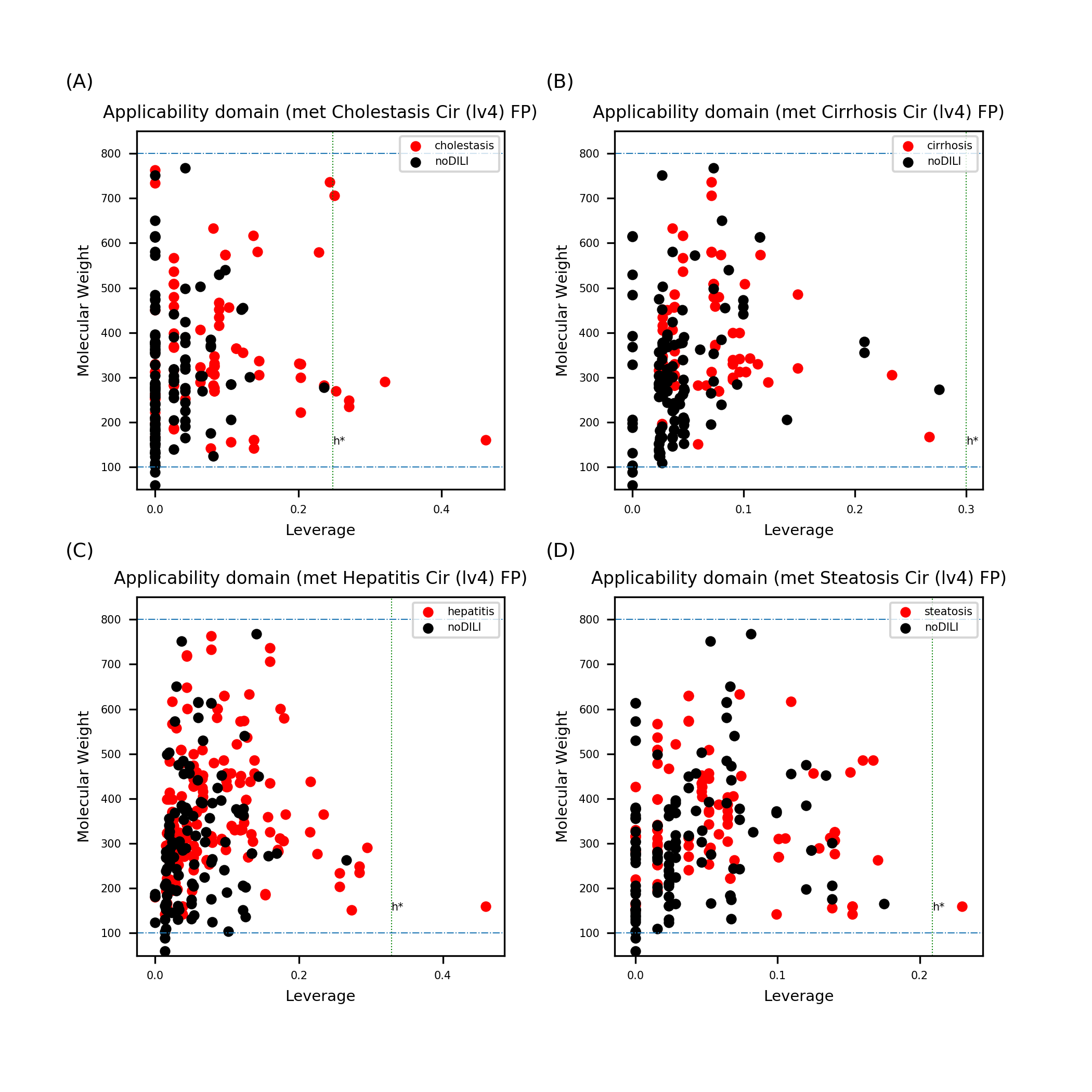


Figure S37. Leverage and MW of drug metabolite sets with Morgan FP (level 4). Data points exceeding the warning leverage were found in all data sets except cirrhosis.


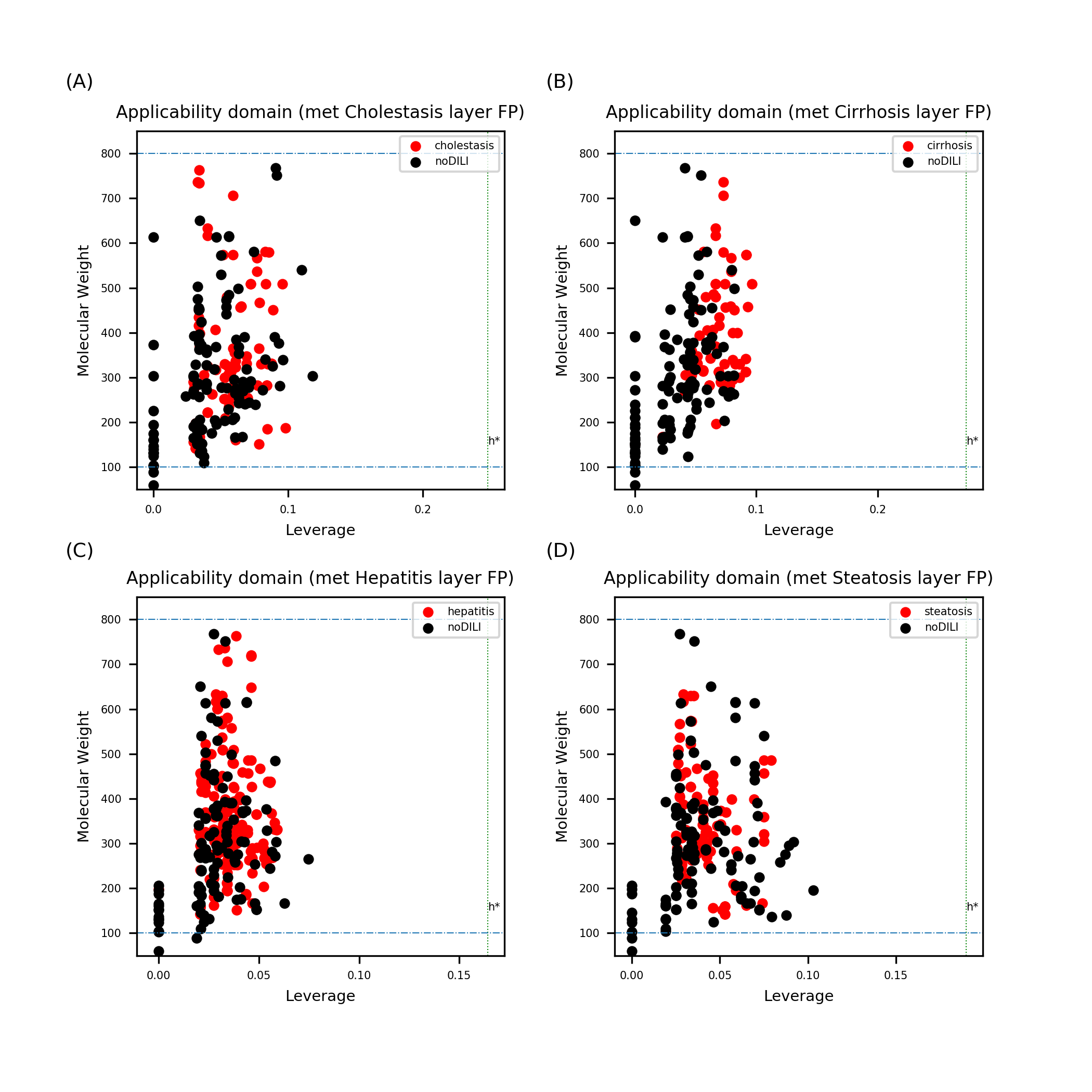


Figure S38. Leverage and MW of drug metabolite sets with Layer FP. No data points were found to exceed the warning leverage.


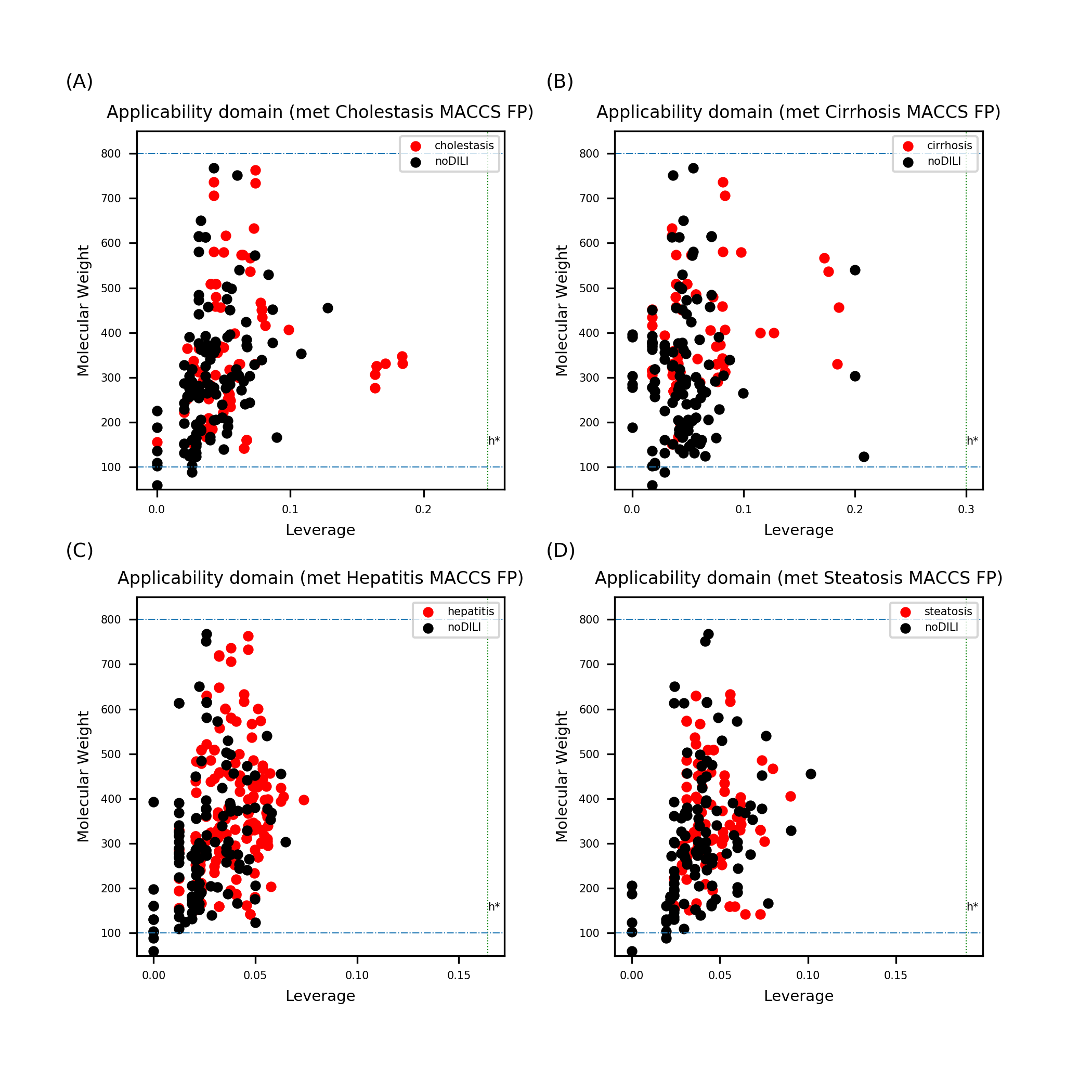


Figure S39. Leverage and MW of drug metabolite sets with MACCS FP. No data points were found to exceed the warning leverage.


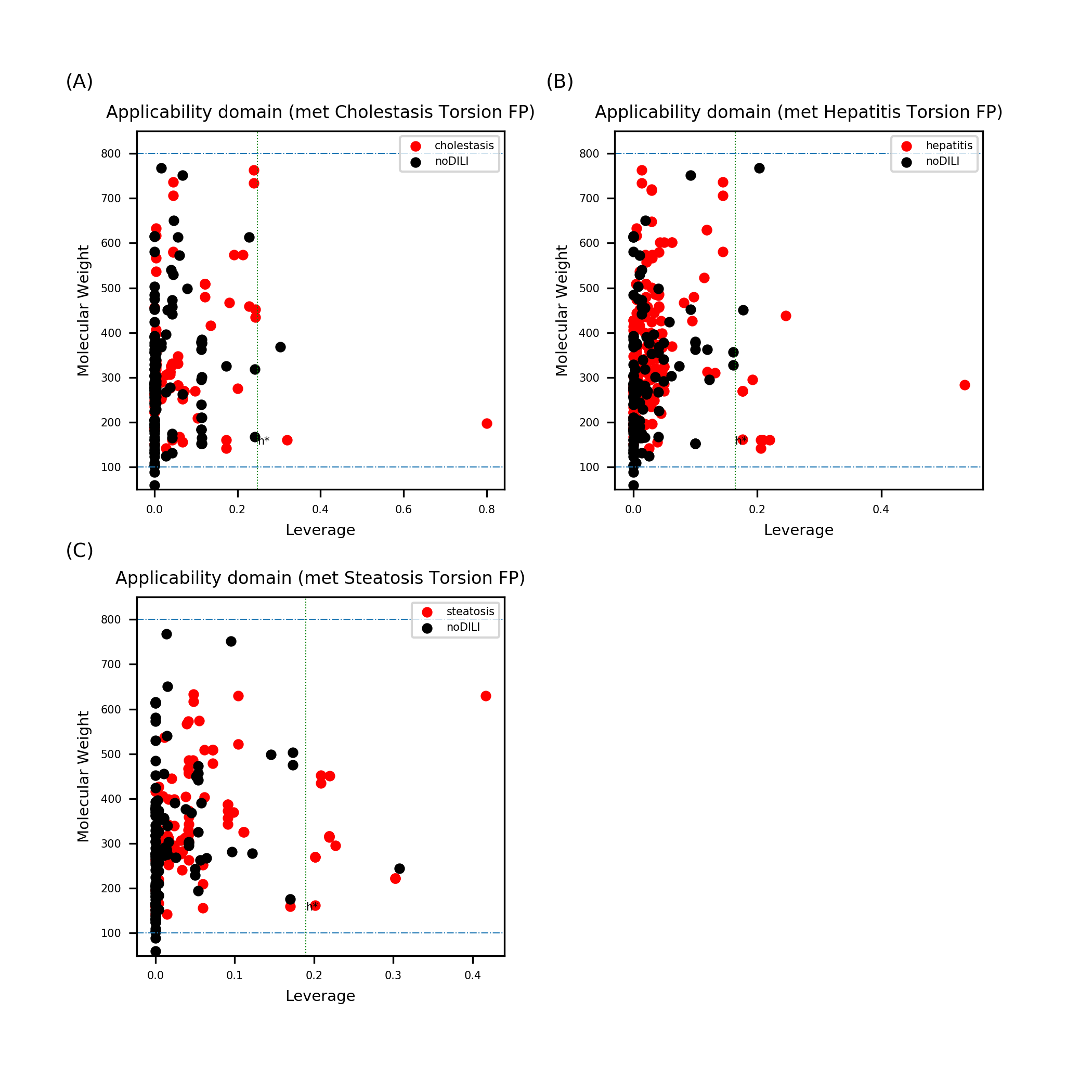


Figure S40. Leverage and MW of drug metabolite data sets with topological-torsion FP. Several data points were found to exceed the warning leverage in all data sets. Applicability domain analysis for cirrhosis is not presented since leverage wasn’t calculated from cirrhosis drug metabolite data set with topological-torsion FP. Selected feature of the data set was a singular matrix, which makes it impossible to calculate inverse matrix of the descriptor matrix.
